# Supplementary material for: A population modification gene drive targeting both Saglin and Lipophorin impairs Plasmodium transmission in Anopheles mosquitoes
Source: eLife. 2023 Dec 5;12:e93142. doi: 10.7554/eLife.93142 (PMC10786457; doi:10.7554/eLife.93142)

Figure 1A – original image with relevant labels

Double-stranded RNA injected in the mosquitoes
for RNAi:

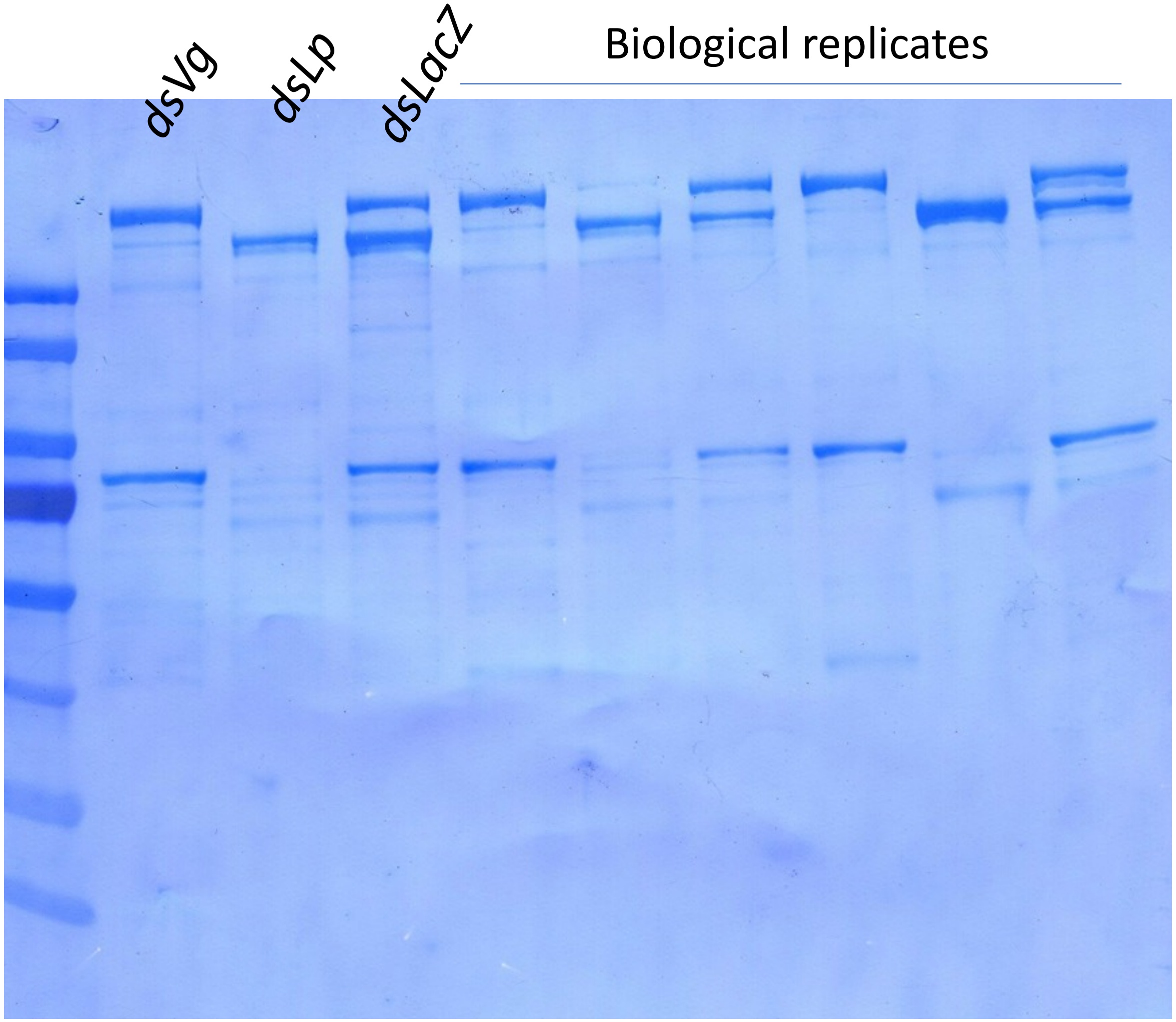

Supplement: Figure 1—source data 1. — Mosquitoes were injected with the indicated double-stranded RNA to silence either Vg or Lp and offered a blood meal after 2 days. Hemolymph was collected 42 hr post blood feeding. [file elife-93142-fig1-data1.pdf]
