# Supplementary figures and images for: A population modification gene drive targeting both Saglin and Lipophorin impairs Plasmodium transmission in Anopheles mosquitoes
Source: eLife. 2023 Dec 5;12:e93142. doi: 10.7554/eLife.93142 (PMC10786457; doi:10.7554/eLife.93142)

## Slide 1
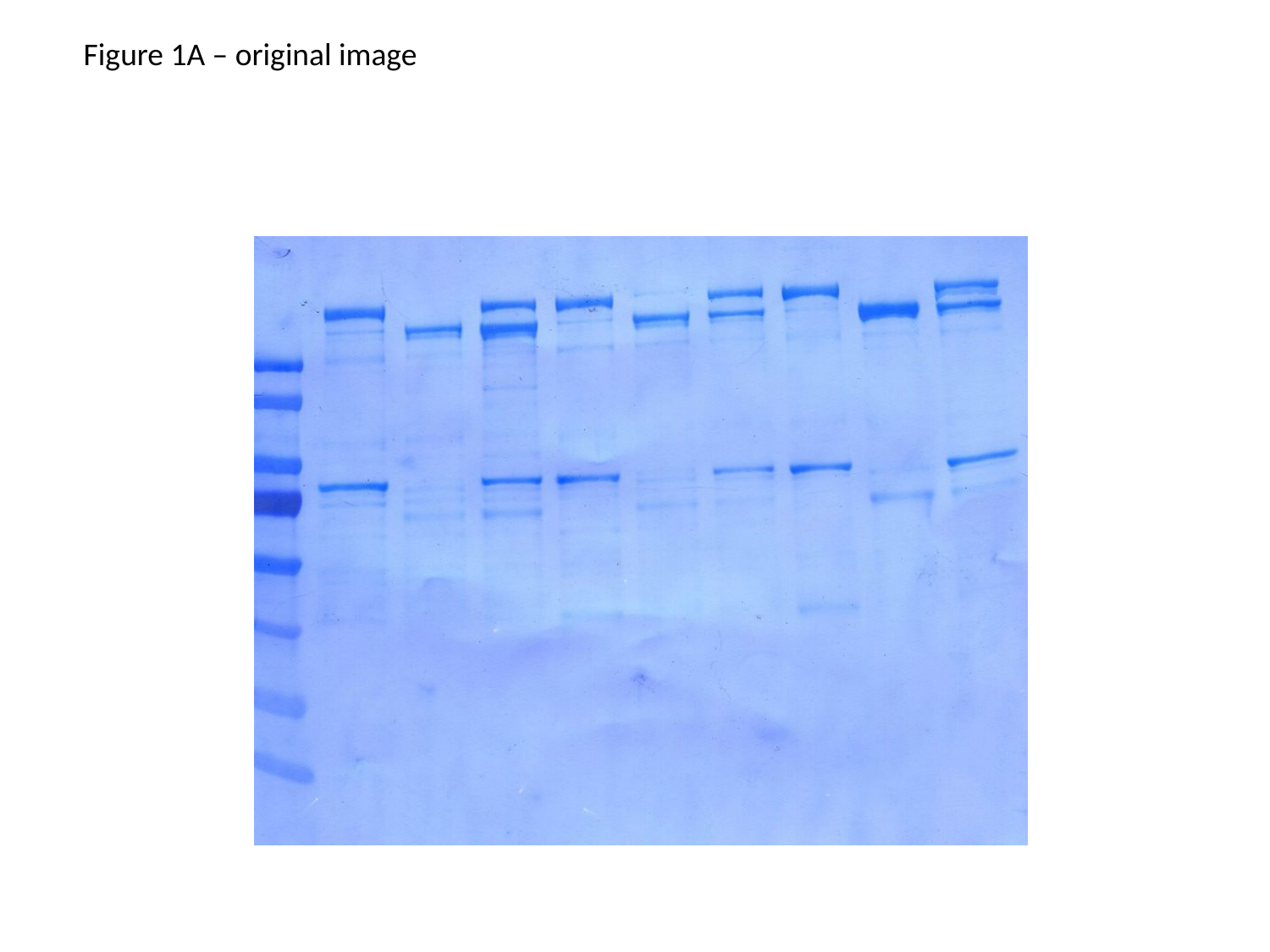

Figure 1A – original image

Supplement: Figure 1—source data 2. — Unlabelled version of Figure 1—source data 1. [file elife-93142-fig1-data2.pptx]

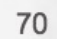

Supplement: Figure 1—source data 3. — First and 7th lanes: protein size ladder, second lane: hemolymph from WT mosquitoes. Lanes c, d: hemolymph from homozygous Lp::Sc2A10 mosquitoes. Lanes a, b: hemolymph from mosquitoes expressing a distinct Lp::ScFv fusion not further discussed in this work. [file elife-93142-fig1-data3.pdf]

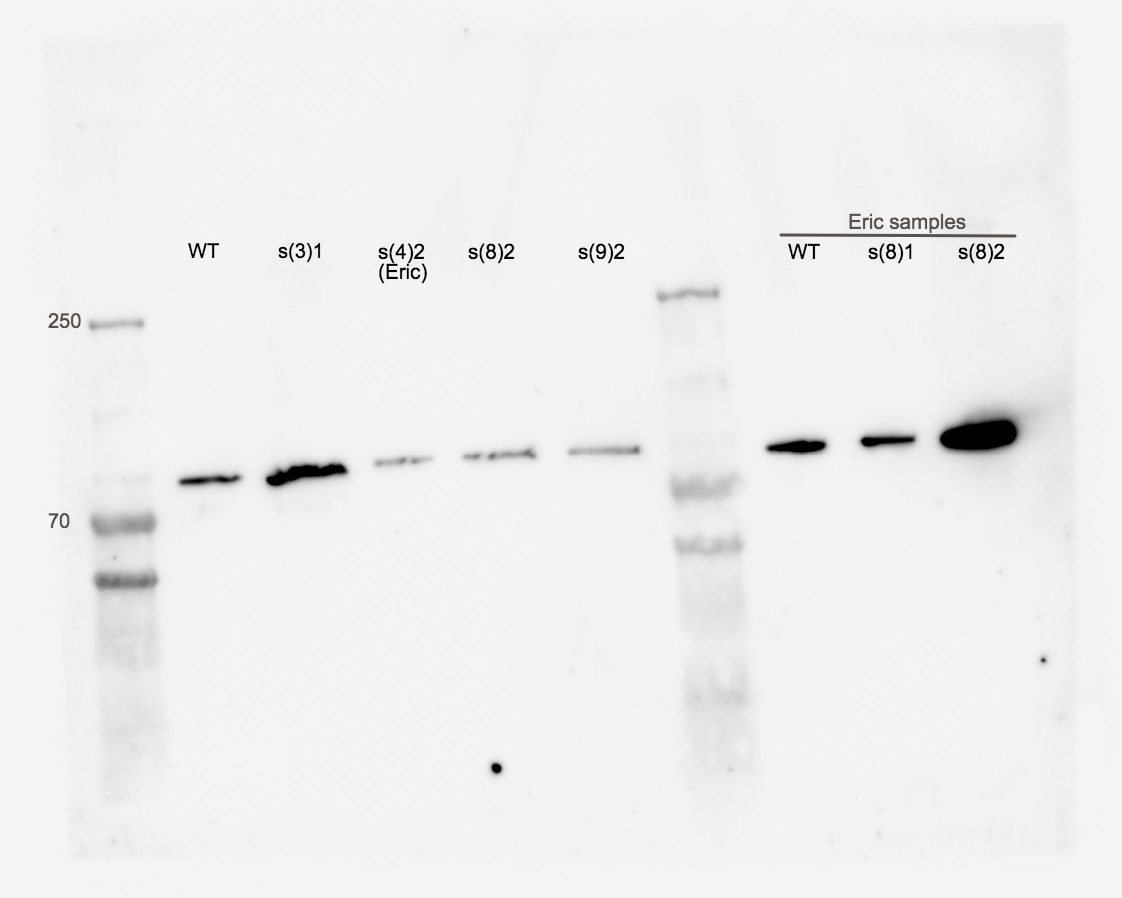

Supplement: Figure 1—source data 4. — Unlabelled version of Figure 1—source data 3. [file elife-93142-fig1-data4.zip › 27-09-2023-RA-RC-eLife-93142/Figure 1Dsource data original image.jpg]

Figure 3C

Membrane probed with Anti-Saglin antibody

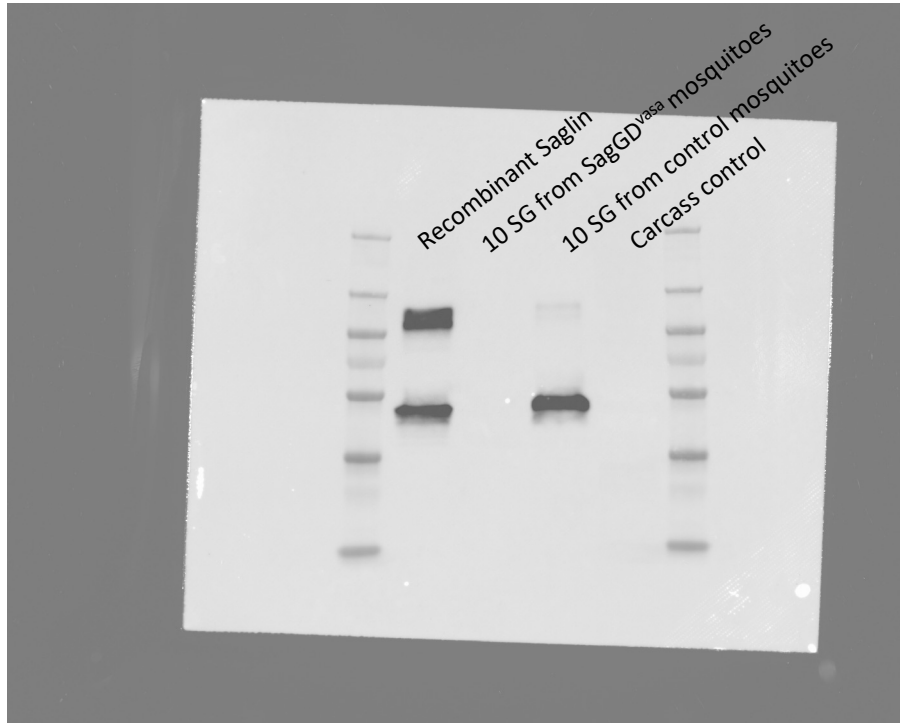

Membrane probed with control human serum

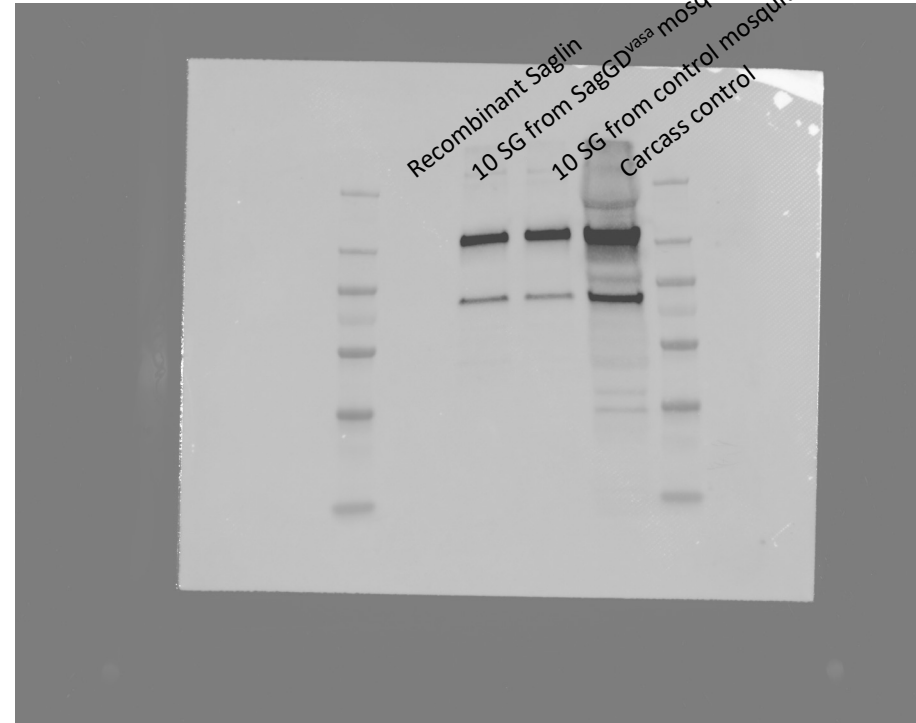

Supplement: Figure 5—source data 1. — The same membrane (right image) was re-probed with serum from a human volunteer regularly bitten by mosquitoes, providing a loading control with salivary and carcass protein signals. [file elife-93142-fig5-data1.pdf]

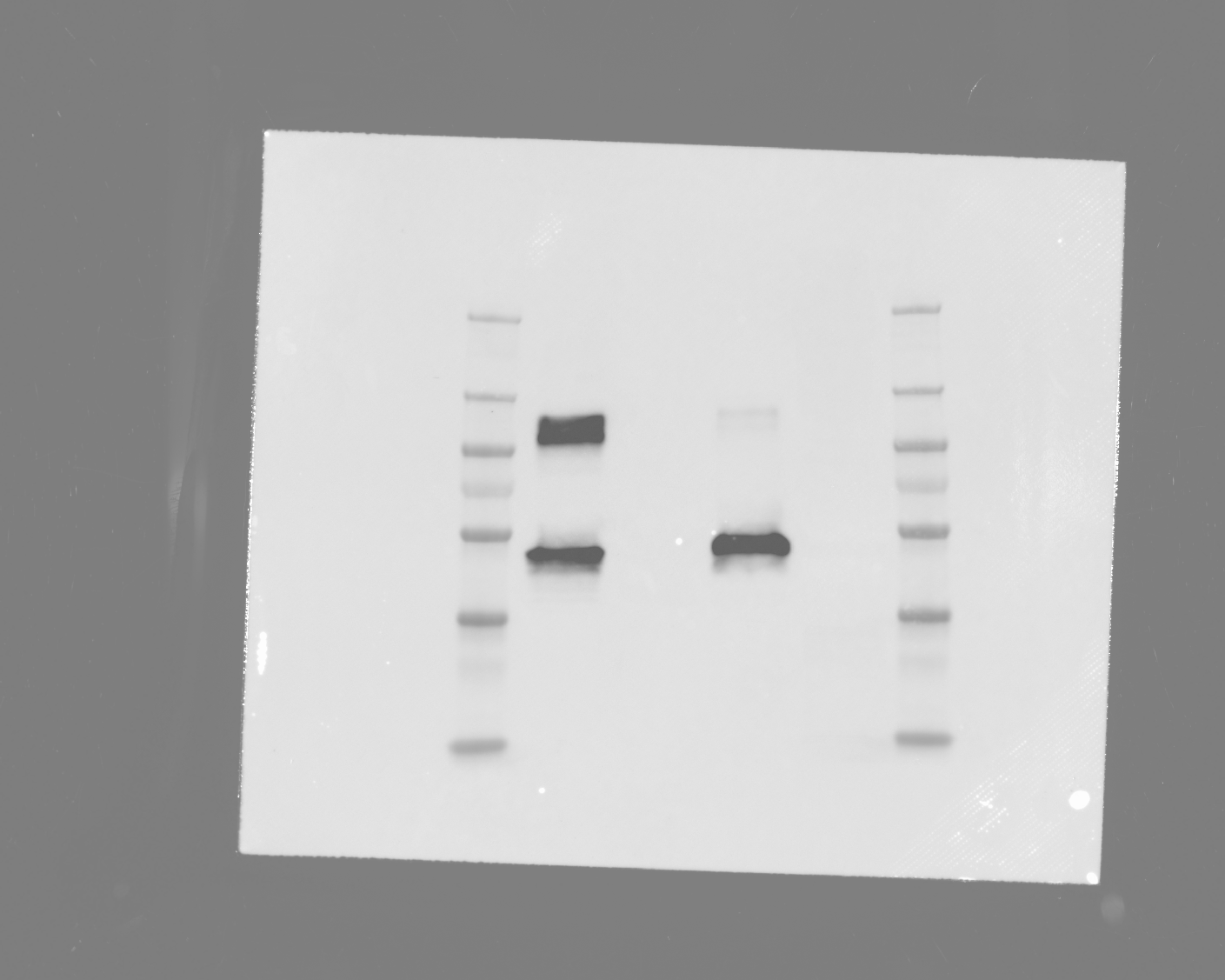

Supplement: Figure 5—source data 2. [file elife-93142-fig5-data2.zip › 27-09-2023-RA-RC-eLife-93142/Figure 5Csource data1 Saglin.tif]

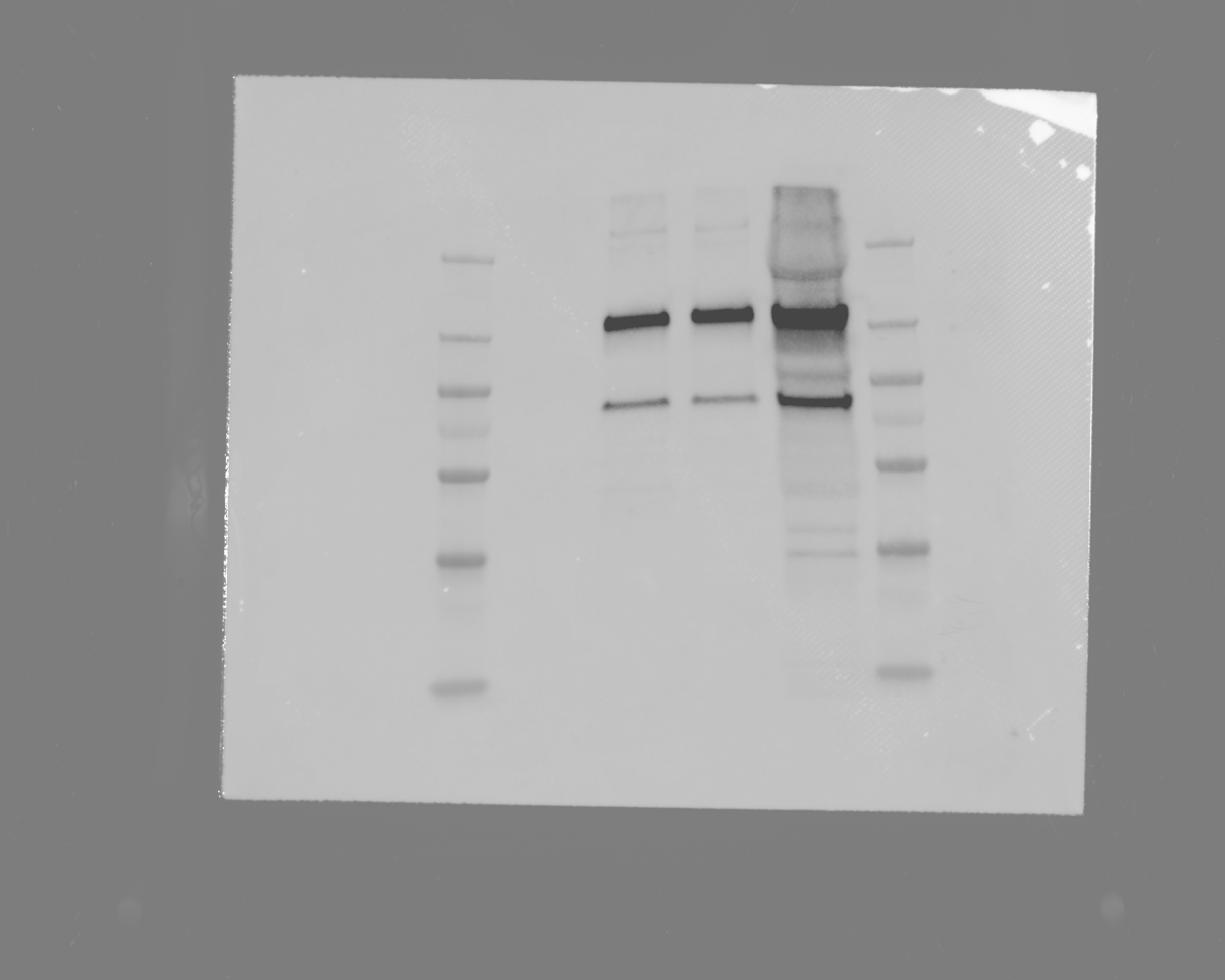

Supplement: Figure 5—source data 3. [file elife-93142-fig5-data3.zip › 27-09-2023-RA-RC-eLife-93142/Figure 5Csource data2Control.tif]

Figure 9A – source images - labeled

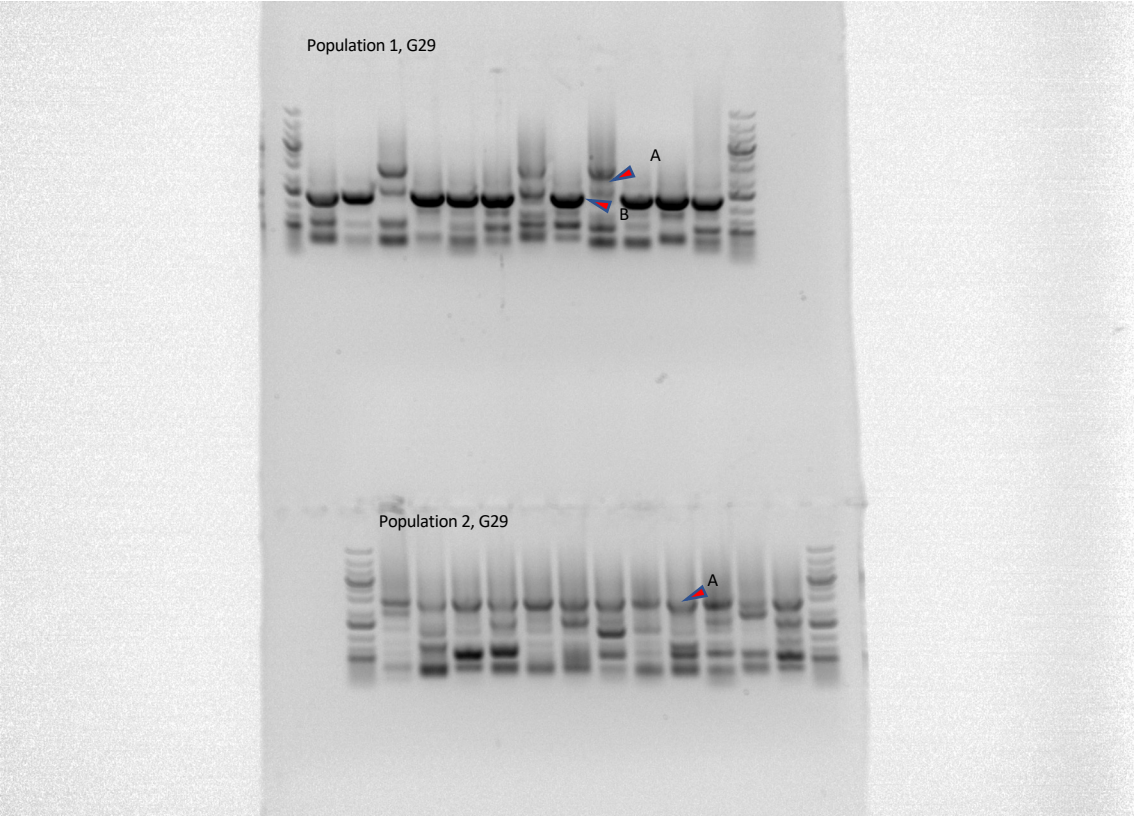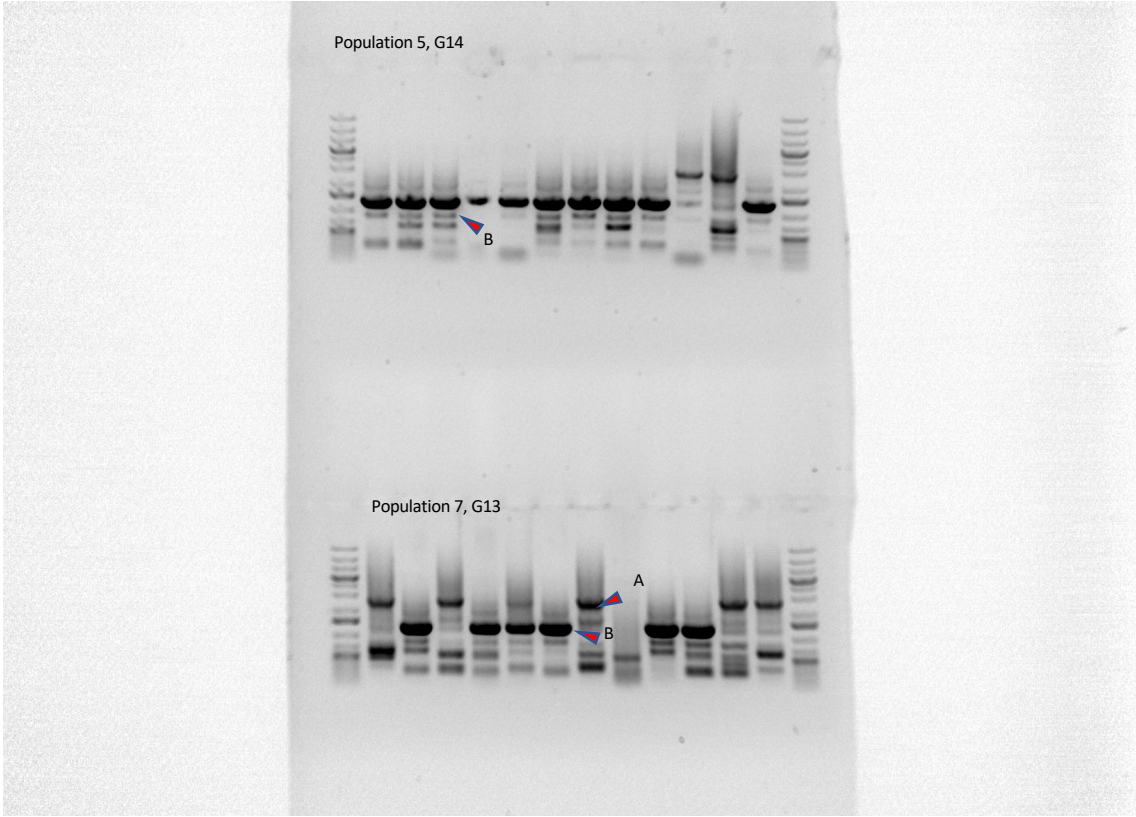

Supplement: Figure 9—source data 1. — For labels, see Figure 9A. [file elife-93142-fig9-data1.pdf]

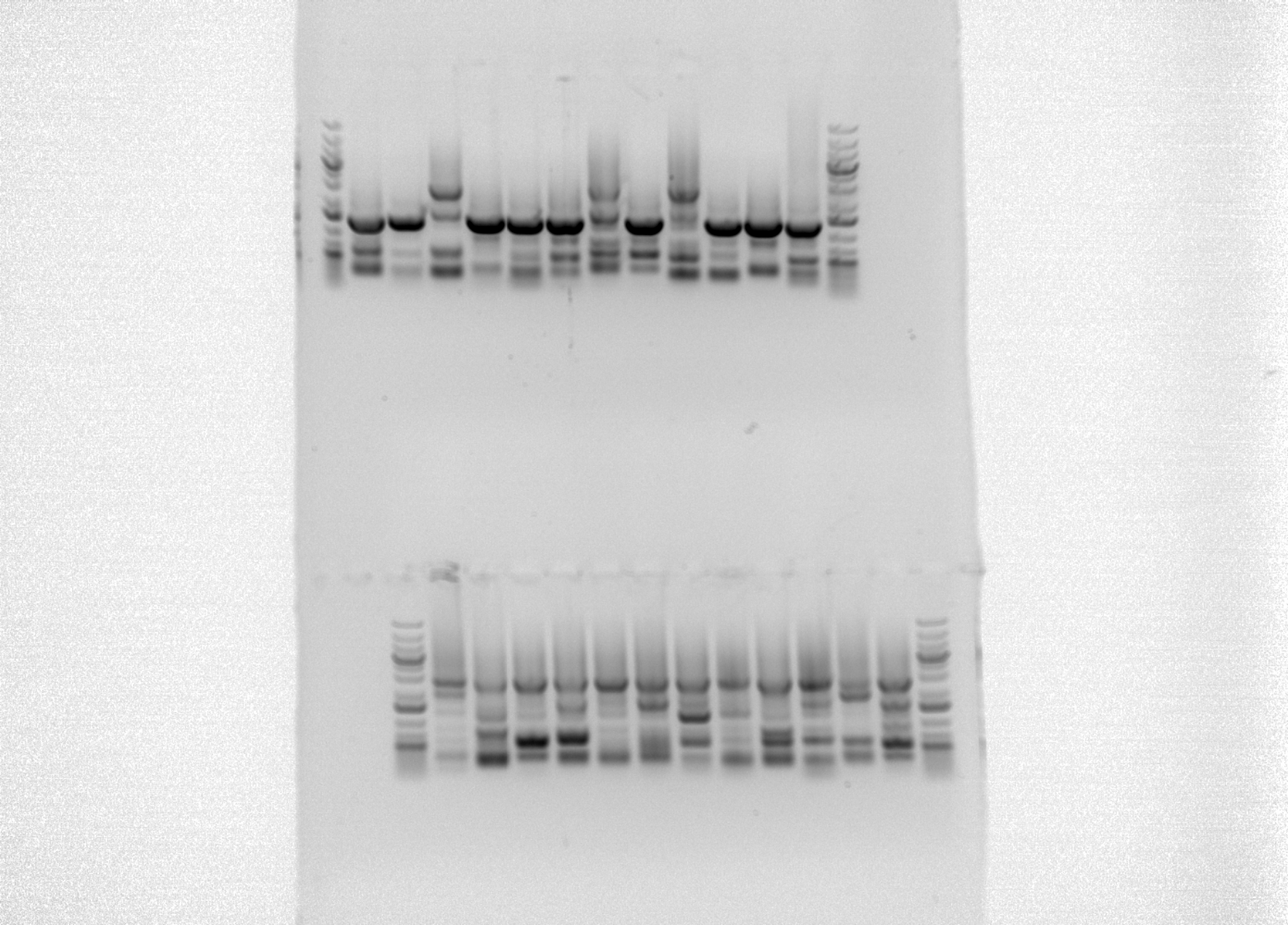

Supplement: Figure 9—source data 2. — For labels, see Figure 9A. [file elife-93142-fig9-data2.zip › 27-09-2023-RA-RC-eLife-93142/Figure 9A source data 1 .tif]

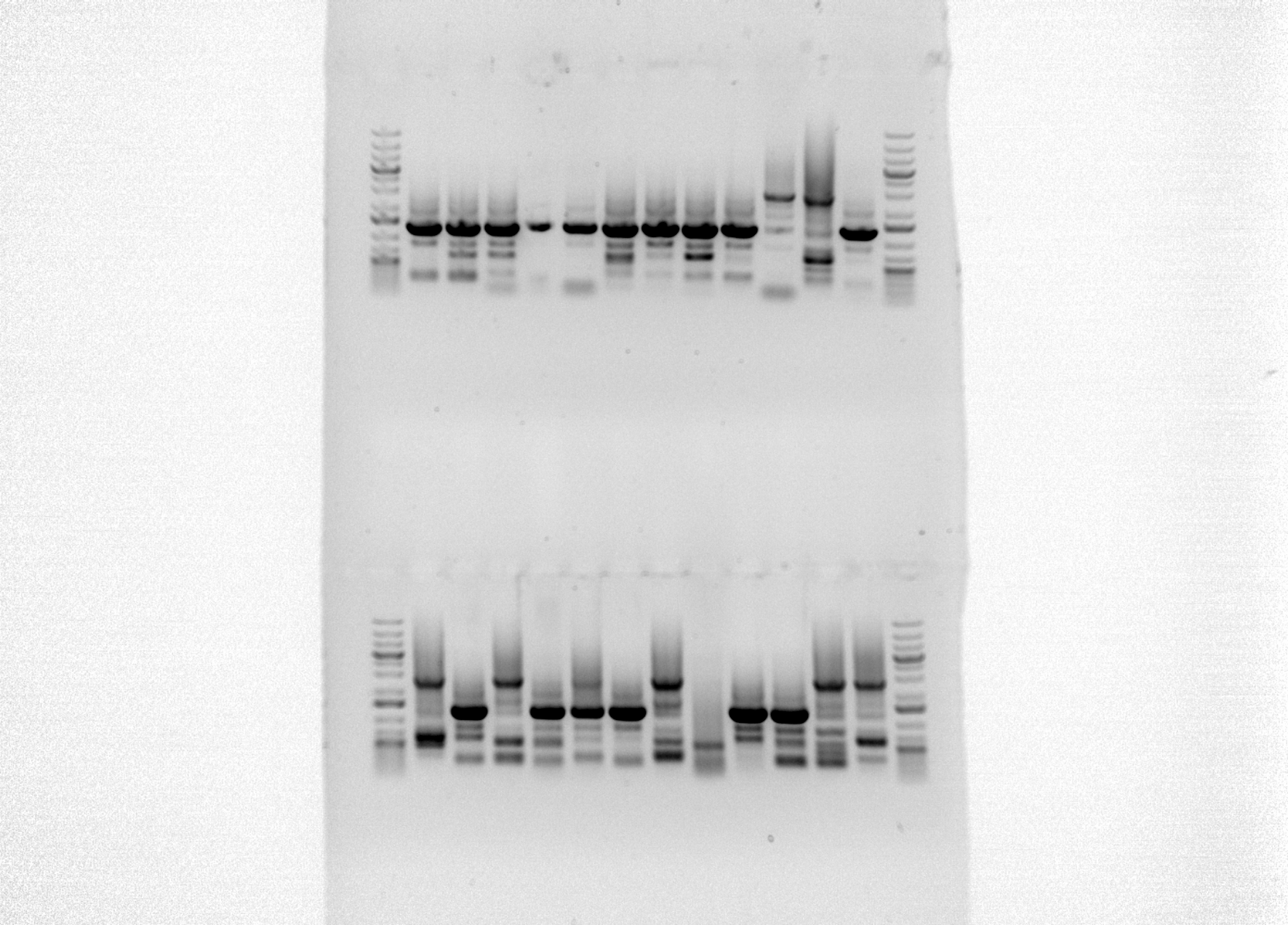

Supplement: Figure 9—source data 3. [file elife-93142-fig9-data3.zip › 27-09-2023-RA-RC-eLife-93142/Figure 9Asource data 2.tif]

Figure 9C – source images – with labels

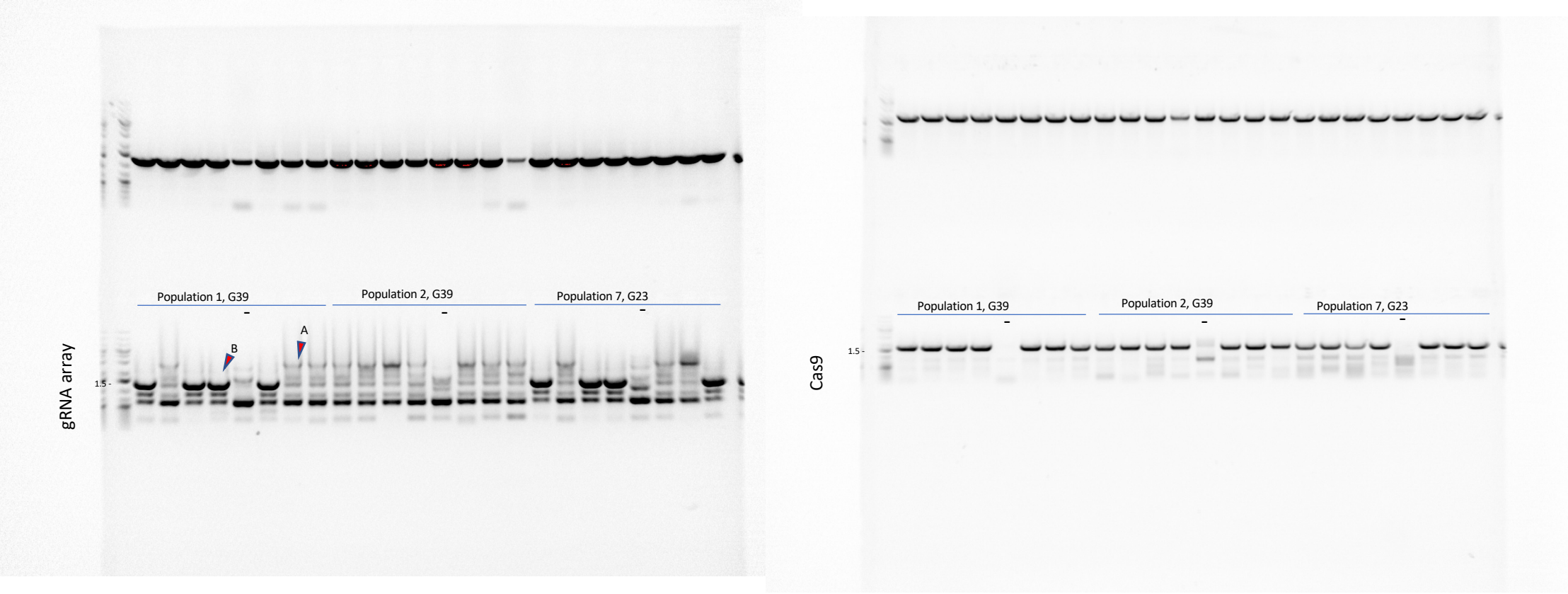

Supplement: Figure 9—source data 4. — For labels, see Figure 9C. [file elife-93142-fig9-data4.pdf]

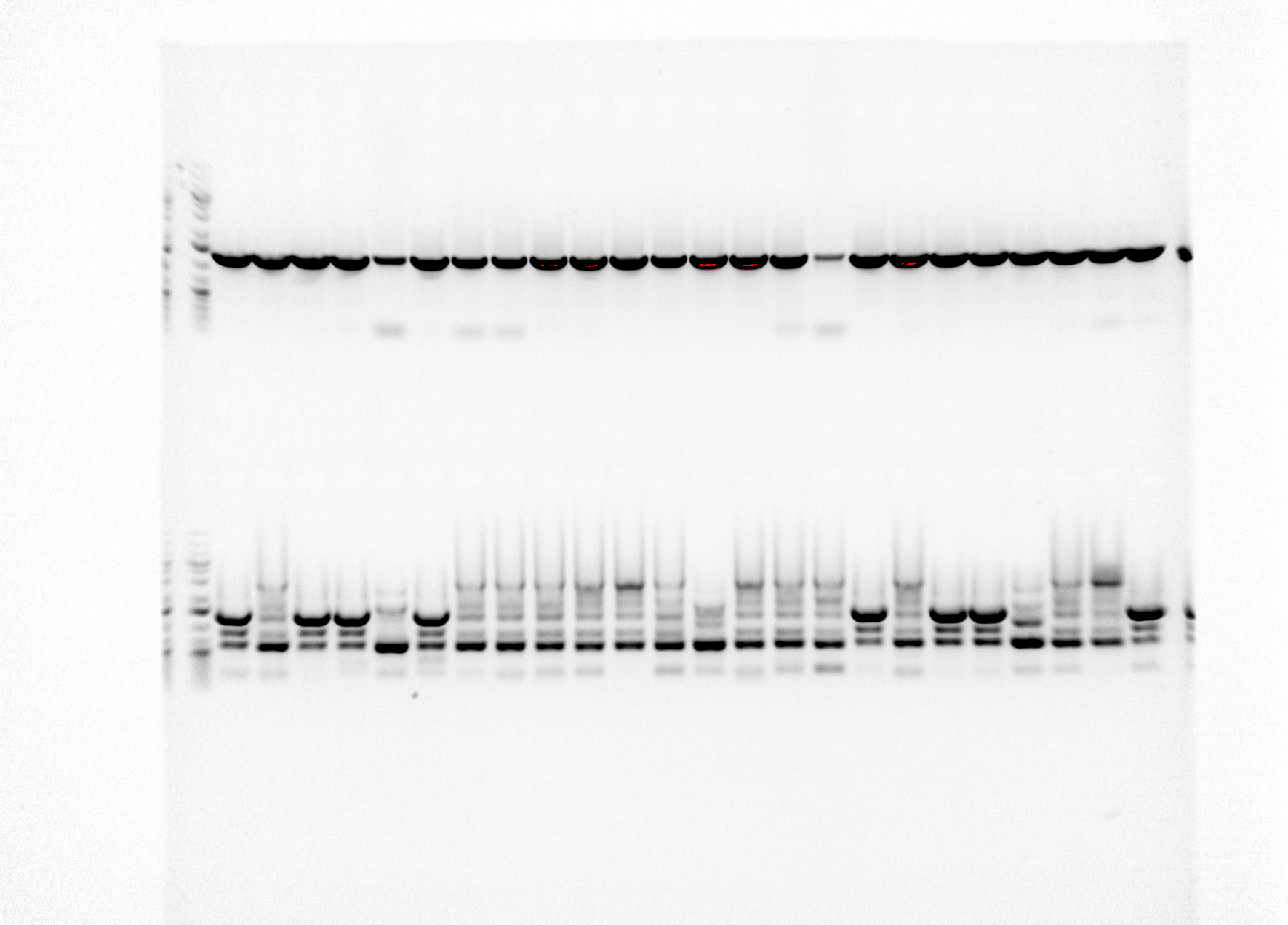

Supplement: Figure 9—source data 5. — For labels, see Figure 9C. [file elife-93142-fig9-data5.zip › 27-09-2023-RA-RC-eLife-93142/Figure 9C source data 1.tif]

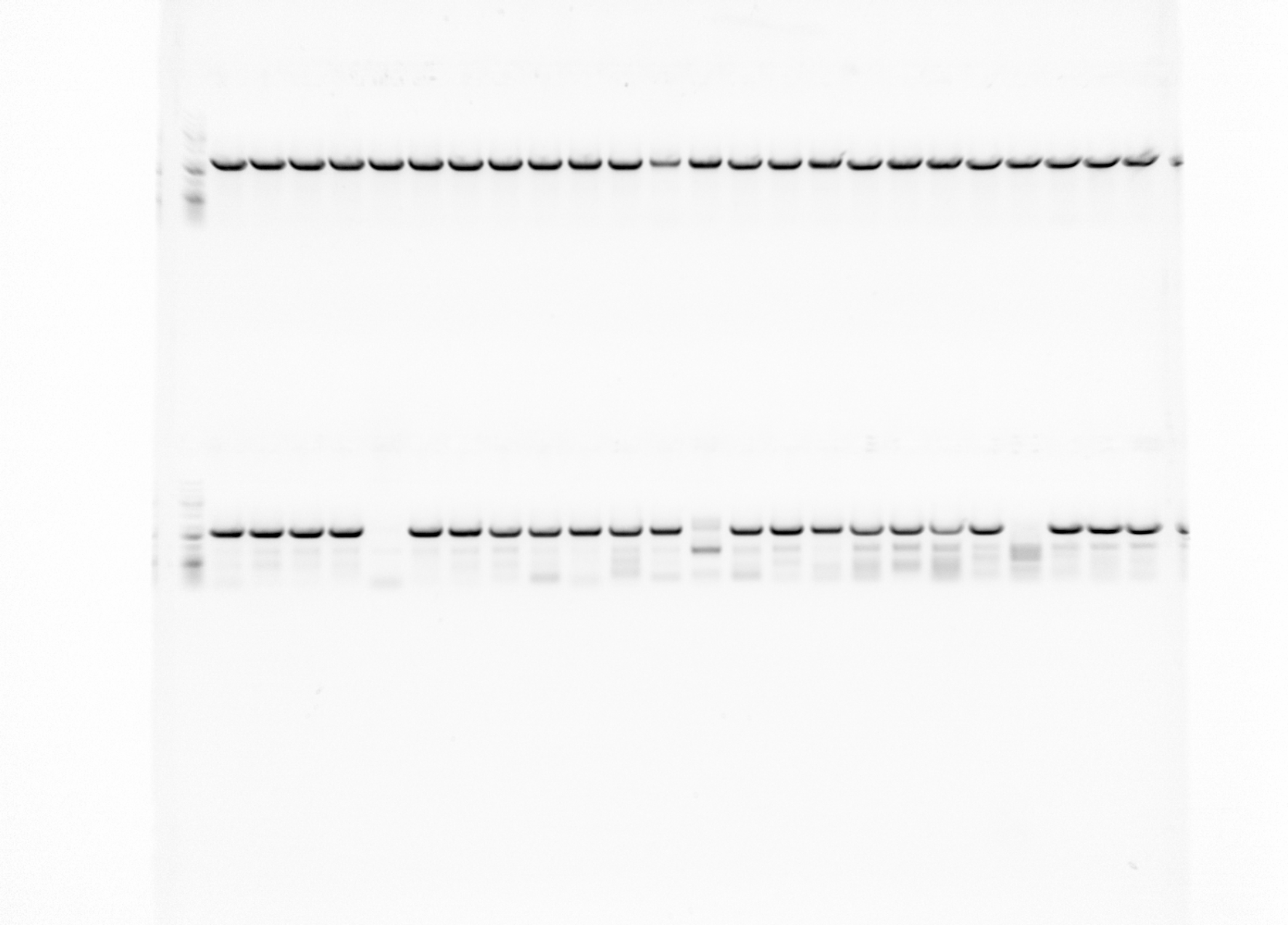

Supplement: Figure 9—source data 6. [file elife-93142-fig9-data6.zip › 27-09-2023-RA-RC-eLife-93142/Figure 9Csource data 2.tif]
