# Supplementary material for: A population modification gene drive targeting both Saglin and Lipophorin impairs Plasmodium transmission in Anopheles mosquitoes
Source: eLife. 2023 Dec 5;12:e93142. doi: 10.7554/eLife.93142 (PMC10786457; doi:10.7554/eLife.93142)
Supplement: Supplementary file 1. [file elife-93142-supp1.docx]

**Supplemental File 1: DNA sequences**

**Supplemental file 1A : *Lp::Sc2A10* knock-in plasmid**

LOCUS pENTR3xgRNALp-Sc2A10 8489 bp DNA circular FEATURES Location/Qualifiers

misc_feature 537..552

/note="M13F"

misc_feature complement(6750..6768)

/note="M13R"

misc_feature 3637..3645

/note=""

misc_feature 3659..3736

/note="re-coded signal peptide"

/note=""

misc_feature 3737..4090

/note="heavy chain_2A10%E2%80%9D misc_feature

3737..4465 /note=%222A10"

misc_feature 4502..4507

/note="perfect intron splice acceptor site"

misc_feature 4475..4486

/note="cleavage site"

misc_feature 4466..4498

/note="encompasses cleavage site"

misc_feature complement(4520..5668)

/note="3xP3-GFP-pAtub56D"

misc_feature 4499..4519

/note="PRESERVE"

misc_feature 4097..4141

/note="(G4S)3-linker"

misc_feature 4142..4465

/note="light chain_2A10%E2%80%9D misc_feature

4487..4507 /note=%22peptide added to ApoII"

misc_feature complement(4520..4688)

/note="Dm beta-Tub56D terminator"

misc_feature 4469..4687

/note="gBlock"

misc_feature 4689..5407

/note="GFP"

misc_feature complement(4575..4580)

/note="polyA site"

misc_feature 5619..5668

/note="3xPax6 binding sites"

misc_feature 5572..5579

/note="TATA box"

misc_feature 6688..6688

/note="mutated to T"

misc_feature 2133..3652

/note="Lp promoter 5%82%C4%F4flk HA"

misc_feature complement(906..1226)

/note="AGAP013557 U6 prom"

misc_feature complement(810..881)

/note="pX330 chimeric guide RNA scaffold"

misc_feature complement(875..881)

/note="CRISPR end"

misc_feature complement(807..870)

/note="tracer"

misc_feature 774..809

/note="from Ag U6"

misc_feature complement(1359..1679)

/note="AGAP013557 U6 prom"

misc_feature complement(1263..1334)

/note="pX330 chimeric guide RNA scaffold"

misc_feature complement(1328..1334)

/note="CRISPR end"

misc_feature complement(1260..1323)

/note="tracer"

misc_feature 1231..1262

/note="from Ag U6"

misc_feature complement(1812..2132)

/note="AGAP013557 U6 prom"

misc_feature complement(1716..1787)

/note="pX330 chimeric guide RNA scaffold"

misc_feature complement(1781..1787)

/note="CRISPR end"

misc_feature complement(1713..1776)

/note="tracer"

misc_feature 1684..1715

/note="from Ag U6"

misc_feature complement(882..905)

/note="sgRNA1 EM1096"

misc_feature 1335..1358

/note="sgRNA2 EM1098"

misc_feature 1788..1811

/note="sgRNA3 EM1100"

misc_feature complement(6701..6726)

/note="EM1103 gRNA2 target"

misc_feature 5671..6697

/note="Lp 3%82%C4%F4flk HA"

ORIGIN

1 CTTTCCTGCG TTATCCCCTG ATTCTGTGGA TAACCGTATT ACCGCCTTTG AGTGAGCTGA

61 TACCGCTCGC CGCAGCCGAA CGACCGAGCG CAGCGAGTCA GTGAGCGAGG AAGCGGAAGA

121 GCGCCCAATA CGCAAACCGC CTCTCCCCGC GCGTTGGCCG ATTCATTAAT GCAGCTGGCA

181 CGACAGGTTT CCCGACTGGA AAGCGGGCAG TGAGCGCAAC GCAATTAATA CGCGTACCGC

241 TAGCCAGGAA GAGTTTGTAG AAACGCAAAA AGGCCATCCG TCAGGATGGC CTTCTGCTTA

301 GTTTGATGCC TGGCAGTTTA TGGCGGGCGT CCTGCCCGCC ACCCTCCGGG CCGTTGCTTC

361 ACAACGTTCA AATCCGCTCC CGGCGGATTT GTCCTACTCA GGAGAGCGTT CACCGACAAA

421 CAACAGATAA AACGAAAGGC CCAGTCTTCC GACTGAGCCT TTCGTTTTAT TTGATGCCTG

481 GCAGTTCCCT ACTCTCGCGT TAACGCTAGC ATGGATGTTT TCCCAGTCAC GACGTTGTAA

541 AACGACGGCC AGTCTTAAGC TCGGGCCCCT ACAGGTCACT AATACCATCT AAGTAGTTGA

601 TTCATAGTGA CTGGATATGT TGTGTTTTAC AGTATTATGT AGTCTGTTTT TTATGCAAAA

661 TCTAATTTAA TATATTGATA TTTATATCAT TTTACGTTTC TCGTTCAACT TTTCTATACA

721 AAGTTGGTAC CGGGCCCCCC GCTAGCGTCG ACGGTATCGA TAAGCTTGAT cggatccGAT

781 GAAAATAAGA AAAACATTTG ACAAAAAAAg caccgactcg gtgccacttt ttcaagttga

841 taacggacta gccttatttt aacttgctaT TTCtagctct aaaacGGTGT CGCTAGTGCT

901 GATTCAAGGA CGAGGGGAAA AAAGGTTGTA TATATACTTT GCGCTTTCAA TCCTTGCTCT

961 AGCGATGCAT AAAGGATATT CAAGAAGGAT TTTTTGCCTG AGTGTGACTG TTATAAGGTT

1021 TTGATCCTGC CGTAACTCAC GCTAATGTGA TGTTTCATAA ACTTTTCAAC ATTTCTTGTA

1081 TTGCTTCATC TCTTTTTTAA TCAAAAGTTA CTATTCATTG TAATACGTTG AAATATCAAA

1141 AAAGAATAAA CAGTTTTCCT ATGATTAATT CAAAAACAGA GTTTTCTAGA ATAACCAAGA

1201 GCCAAACCGC TTCAGTGTAT ATGTGGggaa GATGAAAATA AGAAAAACAT TTGACAAAAA

1261 AAgcaccgac tcggtgccac tttttcaagt tgataacgga ctagccttat tttaacttgc

1321 taTTTCtagc tctaaaacTC CCTGACGATA TTTCACGCAA GGACGAGGGG AAAAAAGGTT

1381 GTATATATAC TTTGCGCTTT CAATCCTTGC TCTAGCGATG CATAAAGGAT ATTCAAGAAG

1441 GATTTTTTGC CTGAGTGTGA CTGTTATAAG GTTTTGATCC TGCCGTAACT CACGCTAATG

1501 TGATGTTTCA TAAACTTTTC AACATTTCTT GTATTGCTTC ATCTCTTTTT TAATCAAAAG

1561 TTACTATTCA TTGTAATACG TTGAAATATC AAAAAAGAAT AAACAGTTTT CCTATGATTA

1621 ATTCAAAAAC AGAGTTTTCT AGAATAACCA AGAGCCAAAC CGCTTCAGTG TATATGTGGa

1681 gagGATGAAA ATAAGAAAAA CATTTGACAA AAAAAgcacc gactcggtgc cactttttca

1741 agttgataac ggactagcct tattttaact tgctaTTTCt agctctaaaa cCCGAGGACC

1801 CACATCGTTC CAAGGACGAG GGGAAAAAAG GTTGTATATA TACTTTGCGC TTTCAATCCT

1861 TGCTCTAGCG ATGCATAAAG GATATTCAAG AAGGATTTTT TGCCTGAGTG TGACTGTTAT

1921 AAGGTTTTGA TCCTGCCGTA ACTCACGCTA ATGTGATGTT TCATAAACTT TTCAACATTT

1981 CTTGTATTGC TTCATCTCTT TTTTAATCAA AAGTTACTAT TCATTGTAAT ACGTTGAAAT

2041 ATCAAAAAAG AATAAACAGT TTTCCTATGA TTAATTCAAA AACAGAGTTT TCTAGAATAA

2101 CCAAGAGCCA AACCGCTTCA GTGTATATGT GGAACAGTTC ATTCCCGATT GAGGGATTTT

2161 ATTCCCCGGG GGCCTTTTCA AACGGCTTAA TATAAGCAAT TAATAGTATT TTTTCTTTCA

2221 GGTTAGTTTA CTGTAATGGT GTAATTGTCA TCTTACACCT CCGTCTGATA AGAGATTACG

2281 AAGCTCAGTA TGATGAAATA AATAAGATAA ATTTATTTAA AAAAGAACAA TTGCTATGAG

2341 AGTGAAATAC AACAGTGGCG TTCACAATAT TCGAAAAACA ATAAAATTAA AAAAAAAACA

2401 AGAAAACATT CACAAACATA TCAATCTGCT TTCATCGACA CCGAACTGCT AGCCTCCCCA

2461 GTCTAACCGC GGTGGGGACG TTTAATTGCC TTTGTTCTCG CACCCGGTCA AACATACACT

2521 TCGGACCTTG CTCCGAACCC CACTGTAATC CCTAGCTCGT CATCATCATT GCCGGCATCA

2581 TGCTAAGCGT GCATTATTTT CACAACTTAG CGTAATGCTA GCGTGCGCTA GCAACAAACT

2641 CGCCCGCAGA CTCGTCACAG CACCGGTACG ATCGATCGTT TACCGTTCCC TTTCCCGATC

2701 GGGTTGGCTG CGATATCCGT GTCCGGGTAG AAAACTTCCC CTTTTACACA CACACACTCA

2761 CATACACACA GAGCTGAATA GCAACTTACC TTATCTGTTC GTCATCGCTC GGCCGGATCT

2821 GGACGAATCT TCGCACCGAT AACCATGTGG ATCTACGACC TCCGCTTGGC TGTCTCTCTG

2881 CTCATGTGTA TGTCTGTGTG TGTGTGTATG TGAGCTTCTT CCCTCAAATC CCTCGATCTC

2941 GCTGTGCCAA CAATCAAACG TGCAAGTGCA AACATTGCAC CCCATTGATT ATACACCAAC

3001 ACCAACCAAT TCCCCTTGCG GAGGCATCTC TGTGCTCGGC AGCATGTTTA CCGCAGATCT

3061 ACAGAGAACT TCAATTGAGG TCCTTTCCAC CCCCAGCCCT CAACCGGCAA TCCGGCAGCC

3121 ACTGGATCAT CAGCGAAAGA GAGAGAGAGC AGAGCAGAAC AGAGGTGACC AACTGTGGTA

3181 TCGCTTCCCG CGCGCCGGTG TGTTGGTGTC CATTTCGGTG ATCGCGATCC CGGCCGCTTC

3241 CAGCACCGTC CACCGATCAG TCACAAAAAC GCTCTCCAAA CCCCTTATCA GCACCGTTCG

3301 CTGATGTGAA CCCCCGTTCA AACCCCAAAT GCAGTGTTTG TATTGCTGTG TGTATGTGTA

3361 CGTGCGTGTG TGTGGAAATT CTATAAAAGT AGGCACCCGT GGCCGGGATC CGTTATTCCC

3421 GTCCTGAGGC CCGCCCGGGA TCGCTGGTGA CGACAGACGA GCCGCTGTGT GACGTACGTA

3481 GTGCCCGATC GGTAAAGAGT GAACCGTCTT CTCTGCAGTG TAGGAGAGAA CGGTTTCATC

3541 TTTTTCGCCC ACACCCCCCC GGTTACATTC CATGTTGAAC TGTAAGGTCT AGTGAACATT

3601 TCCTGAGTGT GGAAAGTGTG GTTTAGTGCG TGAGAGTGCA CGGACACGAC ACGGAACGAT

3661 GTGGGTgCTg GGaGGcAGaA GaCTcCTgTG GtcgTTtCTc GTcagctTgG TcCTcATcCA

3721 gtccGTcagC gcagccCAGA TCCAGCTGGT GCAGTCGGGC CCCGAGCTGA AGAAGCCCGG

3781 CGAAACGGTG AAGATCAGCT GTAAGGCGAG CGGTTACACC TTCACCAACT ACGGCATCAA

3841 CTGGGTGAAG CAGGCGCCGG GCAAGGGTCT GAAGTGGATG GGATGGATCA ACACGATCAC

3901 CGAGGAGCCC ACCTTCGCCG AAGAGTTCAC GGGACGCTTC GCCTTCAGCC TGGAAACCTC

3961 GGCGAGCACC GCCTACCTCC AGATCAACAA CCTGAAGAAC GAGGACACGG CCACGTACTT

4021 CTGTGCGCGC GGCAGCGAGT TCGGACGCCT GGTCTACTGG GGCCAGGGAG CGAGCGTGAC

4081 CGTCTCGAGC TCGACCGGTG GTGGTGGCAG CGGCGGAGGA GGTAGCGGAG GTGGCGGCAG

4141 CGACATCCAG ATGACGCAGA CGACGAGCTC GCTGTCGGCC TCGCTGGGCG ATCGTGTGAC

4201 GATCTCGTGC AGCGCCTCGC AGGGCATCAG CAACTACCTG AACTGGTACC AGCAGAAGCC

4261 GGACGGTACC GTCAAGCTGC TGATCTTCTA CACCTCGACC CTGTACTCGG GTGTGCCGTC

4321 GCGTTTCTCG GGTTCGGGCT CGGGCACCGA TTACTCGCTG ACCATCTCGA ACCTGGAGCC

4381 CGAGGACATC GCGACGTACT ACTGCCAGCA GTACTCGCGT TTCCCGTACG TGTTCGGCGG

4441 CGGCACGAAG CTGGAGATCA AGCGCGCcAA GGAaCGtTTC CGtCGCGGaA TtCGtGAaTC

4501 CGCAGGTATG TTCCTATACg aaaccccaac aaaaaccata attgtttAGA CTTGTGAACA

4561 AAATTGGATC CGACTTTATT GATTACGTTG TTAAGAGAAC AAATCTTTTA CAACTGAATT

4621 CATTTGTTCT CGTTTCATTT TTTTTCGCAA AACATTGATC GAGAATTCGA TTGATTTCCG

4681 ATTCGAATTT ACTTGTACAG CTCGTCCATG CCGAGAGTGA TCCCGGCGGC GGTCACGAAC

4741 TCCAGCAGGA CCATGTGATC GCGCTTCTCG TTGGGGTCTT TGCTCAGGGC GGACTGGGTG

4801 CTCAGGTAGT GGTTGTCGGG CAGCAGCACG GGGCCGTCGC CGATGGGGGT GTTCTGCTGG

4861 TAGTGGTCGG CGAGCTGCAC GCTGCCGTCC TCGATGTTGT GGCGGATCTT GAAGTTCACC

4921 TTGATGCCGT TCTTCTGCTT GTCGGCCATG ATATAGACGT TGTGGCTGTT GTAGTTGTAC

4981 TCCAGCTTGT GCCCCAGGAT GTTGCCGTCC TCCTTGAAGT CGATGCCCTT CAGCTCGATG

5041 CGGTTCACCA GGGTGTCGCC CTCGAACTTC ACCTCGGCGC GGGTCTTGTA GTTGCCGTCG

5101 TCCTTGAAGA AGATGGTGCG CTCCTGGACG TAGCCTTCGG GCATGGCGGA CTTGAAGAAG

5161 TCGTGCTGCT TCATGTGGTC GGGGTAGCGG CTGAAGCACT GCACGCCGTA GGTCAGGGTG

5221 GTCACGAGGG TGGGCCAGGG CACGGGCAGC TTGCCGGTGG TGCAGATGAA CTTCAGGGTC

5281 AGCTTGCCGT AGGTGGCATC GCCCTCGCCC TCGCCGGACA CGCTGAACTT GTGGCCGTTT

5341 ACGTCGCCGT CCAGCTCGAC CAGGATGGGC ACCACCCCGG TGAACAGCTC CTCGCCCTTG

5401 CTCACCATGG TGGCGACCGG TGGATCCCGG GCCCGCGGTA CCGTCGACTC TAGCGGTACC

5461 CCGATTGTTT AGCTTGTTCA GCTGCGCTTG TTTATTTGCT TAGCTTTCGC TTAGCGACGT

5521 GTTCACTTTG CTTGTTTGAA TTGAATTGTC GCTCCGTAGA CGAAGCGCCT CTATTTATAC

5581 TCCGGCGGTC GAGGGTTCGA AATCGATAAG CTTGGATCCT AATTGAATTA GCTCTAATTG

5641 AATTAGTCTC TAATTGAATT AGATCCCCGA TGGAAGAGAT GGCGAAGGTT CTCATCCACA

5701 AGTTGTTGGT GATGCTAACA TTGCTTCCGA GCAATGTATT GCTGCCGAGG GTGTCGATTA

5761 GTACAGCAAA GCGCGAGAAA CAAGTGGCTG CTGTGGCCGG CGGTACCCAT AAACCAGTTG

5821 AGATAATTTA GACAATAAAT TGTAATAGGC AATTTGATGA CGTCCCGCAT GTACGGATCG

5881 TCAACGCTGC ACGCTCGGGA ATAGGGTGAG AAGTGCTGCC CGTTTGTTGG ACAGCGCAGG

5941 TGGTGATCGT GTTGTTTTGG TATCACGAAA ACAAGATAGA ATCTGAGAAA AAAGAACCTT

6001 TTTATCACTT TACCCGCACC GATACTGGTC TTTCTTAGAT CATTTCGGGG CGAAGCAAAC

6061 AAGTCAGCAC GGCATACAAA TTTTGGGATA ATCCCTTAGT CAGTGCCTGT GTCTTTCTAA

6121 TGAAGATTGA CAATGGCGGC GGCGGCGGCG GTGGTAGCAA CCAACGCACT GGCCTTTGTT

6181 TTCATACTTT TCAATGAATT CAATTAGCTT AACCAACGAG GTTGTTGTTG ATGTTCTCAC

6241 GGAAGTCAGG AGAAGCTCCG ACGGGCGTGT AGGGTAAGTC GCAGTTCGGC AGCATTCTGG

6301 CAGTGTACAC CCAGCACACC GAGCGTTCTA GAACGCTGAA TCGTTGAAAC AGAGCAAATG

6361 ACTGAAGGGC TGTGTCTATT GCTGGGCTGT ACAGAATGAC GCATTTCGTG TGGCAATGTG

6421 TTTTCGAAAG ATTGAAGCAT TCTGATAGAG CCGTGTGTCA GGAATGTAAA TATTTAGATA

6481 CTGCAGCTAA CTACAAACTG AGAGAGCTTT CAGCTGGCAG GTGTCCCTAC TTGGTGGTCT

6541 TTTCTAGAAA GAATGTAGAA TTGACATGTT GTGATGATTT ACCAAAAACA ATTTGAGAAG

6601 CTAGAGAGTG AATCGTTCTA GCCACCGGAA CCATCTGGAA TGTTGAAGAT TTTTCTGGAA

6661 TATTAGTAGA CAAAAAGAAG GAGGTCTCAA CTCCGAGctt GCGTGAAATA TCGTCAGGGA

6721 CGgcttATCC CCTATAGTGA GTCGTATTAC ATGGTCATAG CTGTTTCCTG GCAGCTCTGG

6781 CCCGTGTCTC AAAATCTCTG ATGTTACATT GCACAAGATA AAAATATATC ATCATGAACA

6841 ATAAAACTGT CTGCTTACAT AAACAGTAAT ACAAGGGGTG TTATGAGCCA TATTCAACGG

6901 GAAACGTCGA GGCCGCGATT AAATTCCAAC ATGGATGCTG ATTTATATGG GTATAAATGG

6961 GCTCGCGATA ATGTCGGGCA ATCAGGTGCG ACAATCTATC GCTTGTATGG GAAGCCCGAT

7021 GCGCCAGAGT TGTTTCTGAA ACATGGCAAA GGTAGCGTTG CCAATGATGT TACAGATGAG

7081 ATGGTCAGAC TAAACTGGCT GACGGAATTT ATGCCTCTTC CGACCATCAA GCATTTTATC

7141 CGTACTCCTG ATGATGCATG GTTACTCACC ACTGCGATCC CCGGAAAAAC AGCATTCCAG

7201 GTATTAGAAG AATATCCTGA TTCAGGTGAA AATATTGTTG ATGCGCTGGC AGTGTTCCTG

7261 CGCCGGTTGC ATTCGATTCC TGTTTGTAAT TGTCCTTTTA ACAGCGATCG CGTATTTCGT

7321 CTCGCTCAGG CGCAATCACG AATGAATAAC GGTTTGGTTG ATGCGAGTGA TTTTGATGAC

7381 GAGCGTAATG GCTGGCCTGT TGAACAAGTC TGGAAAGAAA TGCATAAACT TTTGCCATTC

7441 TCACCGGATT CAGTCGTCAC TCATGGTGAT TTCTCACTTG ATAACCTTAT TTTTGACGAG

7501 GGGAAATTAA TAGGTTGTAT TGATGTTGGA CGAGTCGGAA TCGCAGACCG ATACCAGGAT

7561 CTTGCCATCC TATGGAACTG CCTCGGTGAG TTTTCTCCTT CATTACAGAA ACGGCTTTTT

7621 CAAAAATATG GTATTGATAA TCCTGATATG AATAAATTGC AGTTTCATTT GATGCTCGAT

7681 GAGTTTTTCT AATCAGAATT GGTTAATTGG TTGTAACACT GGCAGAGCAT TACGCTGACT

7741 TGACGGGACG GCGCAAGCTC ATGACCAAAA TCCCTTAACG TGAGTTACGC GTCGTTCCAC

7801 TGAGCGTCAG ACCCCGTAGA AAAGATCAAA GGATCTTCTT GAGATCCTTT TTTTCTGCGC

7861 GTAATCTGCT GCTTGCAAAC AAAAAAACCA CCGCTACCAG CGGTGGTTTG TTTGCCGGAT

7921 CAAGAGCTAC CAACTCTTTT TCCGAAGGTA ACTGGCTTCA GCAGAGCGCA GATACCAAAT

7981 ACTGTTCTTC TAGTGTAGCC GTAGTTAGGC CACCACTTCA AGAACTCTGT AGCACCGCCT

8041 ACATACCTCG CTCTGCTAAT CCTGTTACCA GTGGCTGCTG CCAGTGGCGA TAAGTCGTGT

8101 CTTACCGGGT TGGACTCAAG ACGATAGTTA CCGGATAAGG CGCAGCGGTC GGGCTGAACG

8161 GGGGGTTCGT GCACACAGCC CAGCTTGGAG CGAACGACCT ACACCGAACT GAGATACCTA

8221 CAGCGTGAGC TATGAGAAAG CGCCACGCTT CCCGAAGGGA GAAAGGCGGA CAGGTATCCG

8281 GTAAGCGGCA GGGTCGGAAC AGGAGAGCGC ACGAGGGAGC TTCCAGGGGG AAACGCCTGG

8341 TATCTTTATA GTCCTGTCGG GTTTCGCCAC CTCTGACTTG AGCGTCGATT TTTGTGATGC

8401 TCGTCAGGGG GGCGGAGCCT ATGGAAAAAC GCCAGCAACG CGGCCTTTTT ACGGTTCCTG

8461 GCCTTTTGCT GGCCTTTTGC TCACATGTT

//

**Supplemental File 1B: *Lp::2A10* knockin in genomic context**

LOCUS Lp-2A10 14919 bp DNA linear

FEATURES Location/Qualifiers

misc_feature 1..1622

/note="Lp promoter"

exon 5369..9341

/note="exon_id=AGAP001826-RA-E2.1"

misc_feature 7396..7407

/note="cleavage site"

exon 9406..9535

/note="exon_id=AGAP001826-RA-E3.1"

exon 9610..14919

/note="exon_id=AGAP001826-RA-E4.1"

misc_feature 2472..2477

/note="perfect intron splice acceptor site"

misc_feature 2445..2456

/note="cleavage site"

misc_feature 2436..2468

/note="encompasses cleavage site"

misc_feature complement(2490..3638)

/note="3xP3-GFP-pAtub deletes 3 cas9 target sites in

intron"

misc_feature 1623..1642

/note="disabled gRNA target site”

misc_feature 1630..1649

/note="disabled gRNA target site"

misc_feature 1639..1658

/note="disabled gRNA target site"

misc_feature 1650..1668

/note="disabled gRNA target site"

misc_feature 1641..1659

/note="disabled gRNA target site"

misc_feature 1670..1689

/note="disabled gRNA target site"

misc_feature 1629..1703

/note="re-coded signal peptide"

misc_feature 1683..1703

/note="disabled gRNA target site"

misc_feature 2469..2489

/note="PRESERVED around splice junction”

misc_feature 1707..2060

/note=“Sc2A10 heavy chain"

misc_feature 2067..2111

/note="(G4S)3-linker"

misc_feature 2112..2435

/note=“Sc2A10 light chain"

misc_feature 1707..2435

/note=“Sc2A10"

misc_feature 2457..2477

/note="peptide added to ApoII"

misc_feature 3589..3638

/note="3xPax6 binding sites"

misc_feature 3542..3549

/note="TATA box"

misc_feature 2659..3377

/note="GFP"

misc_feature complement(2490..2658)

/note="Dm beta-Tub56D terminator"

misc_feature complement(2545..2550)

/note="polyA site"

misc_feature complement(4639..4667)

/note="EM894 GGTCTC-> GGTCTT"

misc_feature 4658..4658

/note="mutated to T"

source 1..14919

/dnas_title=“Sc2A10 inserted in Lp genomic fg"

ORIGIN

1 TTGCGGGGAA GACACATTCG AGATACGCTA AGTGATTGAG CGATTACGAT CTAGCAAGAC

61 ATACGTTCAG CTGTGAGAAT AATCATCCAT CTTTCTGCAA TGAACAGTTC ATTCCCGATT

121 GAGGGATTTT ATTCCCCGGG GGCCTTTTCA AACGGCTTAA TATAAGCAAT TAATAGTATT

181 TTTTCTTTCA GGTTAGTTTA CTGTAATGGT GTAATTGTCA TCTTACACCT CCGTCTGATA

241 AGAGATTACG AAGCTCAGTA TGATGAAATA AATAAGATAA ATTTATTTAA AAAAGAACAA

301 TTGCTATGAG AGTGAAATAC AACAGTGGCG TTCACAATAT TCGAAAAACA ATAAAATTAA

361 AAAAAAAACA AGAAAACATT CACAAACATA TCAATCTGCT TTCATCGACA CCGAACTGCT

421 AGCCTCCCCA GTCTAACCGC GGTGGGGACG TTTAATTGCC TTTGTTCTCG CACCCGGTCA

481 AACATACACT TCGGACCTTG CTCCGAACCC CACTGTAATC CCTAGCTCGT CATCATCATT

541 GCCGGCATCA TGCTAAGCGT GCATTATTTT CACAACTTAG CGTAATGCTA GCGTGCGCTA

601 GCAACAAACT CGCCCGCAGA CTCGTCACAG CACCGGTACG ATCGATCGTT TACCGTTCCC

661 TTTCCCGATC GGGTTGGCTG CGATATCCGT GTCCGGGTAG AAAACTTCCC CTTTTACACA

721 CACACACTCA CATACACACA GAGCTGAATA GCAACTTACC TTATCTGTTC GTCATCGCTC

781 GGCCGGATCT GGACGAATCT TCGCACCGAT AACCATGTGG ATCTACGACC TCCGCTTGGC

841 TGTCTCTCTG CTCATGTGTA TGTCTGTGTG TGTGTGTATG TGAGCTTCTT CCCTCAAATC

901 CCTCGATCTC GCTGTGCCAA CAATCAAACG TGCAAGTGCA AACATTGCAC CCCATTGATT

961 ATACACCAAC ACCAACCAAT TCCCCTTGCG GAGGCATCTC TGTGCTCGGC AGCATGTTTA

1021 CCGCAGATCT ACAGAGAACT TCAATTGAGG TCCTTTCCAC CCCCAGCCCT CAACCGGCAA

1081 TCCGGCAGCC ACTGGATCAT CAGCGAAAGA GAGAGAGAGC AGAGCAGAAC AGAGGTGACC

1141 AACTGTGGTA TCGCTTCCCG CGCGCCGGTG TGTTGGTGTC CATTTCGGTG ATCGCGATCC

1201 CGGCCGCTTC CAGCACCGTC CACCGATCAG TCACAAAAAC GCTCTCCAAA CCCCTTATCA

1261 GCACCGTTCG CTGATGTGAA CCCCCGTTCA AACCCCAAAT GCAGTGTTTG TATTGCTGTG

1321 TGTATGTGTA CGTGCGTGTG TGTGGAAATT CTATAAAAGT AGGCACCCGT GGCCGGGATC

1381 CGTTATTCCC GTCCTGAGGC CCGCCCGGGA TCGCTGGTGA CGACAGACGA GCCGCTGTGT

1441 GACGTACGTA GTGCCCGATC GGTAAAGAGT GAACCGTCTT CTCTGCAGTG TAGGAGAGAA

1501 CGGTTTCATC TTTTTCGCCC ACACCCCCCC GGTTACATTC CATGTTGAAC TGTAAGGTCT

1561 AGTGAACATT TCCTGAGTGT GGAAAGTGTG GTTTAGTGCG TGAGAGTGCA CGGACACGAC

1621 ACGGAACGAT GTGGGTgCTg GGaGGcAGaA GaCTcCTgTG GtcgTTtCTc GTcagctTgG

1681 TcCTcATcCA gtccGTcagC gcagccCAGA TCCAGCTGGT GCAGTCGGGC CCCGAGCTGA

1741 AGAAGCCCGG CGAAACGGTG AAGATCAGCT GTAAGGCGAG CGGTTACACC TTCACCAACT

1801 ACGGCATCAA CTGGGTGAAG CAGGCGCCGG GCAAGGGTCT GAAGTGGATG GGATGGATCA

1861 ACACGATCAC CGAGGAGCCC ACCTTCGCCG AAGAGTTCAC GGGACGCTTC GCCTTCAGCC

1921 TGGAAACCTC GGCGAGCACC GCCTACCTCC AGATCAACAA CCTGAAGAAC GAGGACACGG

1981 CCACGTACTT CTGTGCGCGC GGCAGCGAGT TCGGACGCCT GGTCTACTGG GGCCAGGGAG

2041 CGAGCGTGAC CGTCTCGAGC TCGACCGGTG GTGGTGGCAG CGGCGGAGGA GGTAGCGGAG

2101 GTGGCGGCAG CGACATCCAG ATGACGCAGA CGACGAGCTC GCTGTCGGCC TCGCTGGGCG

2161 ATCGTGTGAC GATCTCGTGC AGCGCCTCGC AGGGCATCAG CAACTACCTG AACTGGTACC

2221 AGCAGAAGCC GGACGGTACC GTCAAGCTGC TGATCTTCTA CACCTCGACC CTGTACTCGG

2281 GTGTGCCGTC GCGTTTCTCG GGTTCGGGCT CGGGCACCGA TTACTCGCTG ACCATCTCGA

2341 ACCTGGAGCC CGAGGACATC GCGACGTACT ACTGCCAGCA GTACTCGCGT TTCCCGTACG

2401 TGTTCGGCGG CGGCACGAAG CTGGAGATCA AGCGCGCcAA GGAaCGtTTC CGtCGCGGaA

2461 TtCGtGAaTC CGCAGGTATG TTCCTATACg aaaccccaac aaaaaccata attgtttAGA

2521 CTTGTGAACA AAATTGGATC CGACTTTATT GATTACGTTG TTAAGAGAAC AAATCTTTTA

2581 CAACTGAATT CATTTGTTCT CGTTTCATTT TTTTTCGCAA AACATTGATC GAGAATTCGA

2641 TTGATTTCCG ATTCGAATTT ACTTGTACAG CTCGTCCATG CCGAGAGTGA TCCCGGCGGC

2701 GGTCACGAAC TCCAGCAGGA CCATGTGATC GCGCTTCTCG TTGGGGTCTT TGCTCAGGGC

2761 GGACTGGGTG CTCAGGTAGT GGTTGTCGGG CAGCAGCACG GGGCCGTCGC CGATGGGGGT

2821 GTTCTGCTGG TAGTGGTCGG CGAGCTGCAC GCTGCCGTCC TCGATGTTGT GGCGGATCTT

2881 GAAGTTCACC TTGATGCCGT TCTTCTGCTT GTCGGCCATG ATATAGACGT TGTGGCTGTT

2941 GTAGTTGTAC TCCAGCTTGT GCCCCAGGAT GTTGCCGTCC TCCTTGAAGT CGATGCCCTT

3001 CAGCTCGATG CGGTTCACCA GGGTGTCGCC CTCGAACTTC ACCTCGGCGC GGGTCTTGTA

3061 GTTGCCGTCG TCCTTGAAGA AGATGGTGCG CTCCTGGACG TAGCCTTCGG GCATGGCGGA

3121 CTTGAAGAAG TCGTGCTGCT TCATGTGGTC GGGGTAGCGG CTGAAGCACT GCACGCCGTA

3181 GGTCAGGGTG GTCACGAGGG TGGGCCAGGG CACGGGCAGC TTGCCGGTGG TGCAGATGAA

3241 CTTCAGGGTC AGCTTGCCGT AGGTGGCATC GCCCTCGCCC TCGCCGGACA CGCTGAACTT

3301 GTGGCCGTTT ACGTCGCCGT CCAGCTCGAC CAGGATGGGC ACCACCCCGG TGAACAGCTC

3361 CTCGCCCTTG CTCACCATGG TGGCGACCGG TGGATCCCGG GCCCGCGGTA CCGTCGACTC

3421 TAGCGGTACC CCGATTGTTT AGCTTGTTCA GCTGCGCTTG TTTATTTGCT TAGCTTTCGC

3481 TTAGCGACGT GTTCACTTTG CTTGTTTGAA TTGAATTGTC GCTCCGTAGA CGAAGCGCCT

3541 CTATTTATAC TCCGGCGGTC GAGGGTTCGA AATCGATAAG CTTGGATCCT AATTGAATTA

3601 GCTCTAATTG AATTAGTCTC TAATTGAATT AGATCCCCGA TGGAAGAGAT GGCGAAGGTT

3661 CTCATCCACA AGTTGTTGGT GATGCTAACA TTGCTTCCGA GCAATGTATT GCTGCCGAGG

3721 GTGTCGATTA GTACAGCAAA GCGCGAGAAA CAAGTGGCTG CTGTGGCCGG CGGTACCCAT

3781 AAACCAGTTG AGATAATTTA GACAATAAAT TGTAATAGGC AATTTGATGA CGTCCCGCAT

3841 GTACGGATCG TCAACGCTGC ACGCTCGGGA ATAGGGTGAG AAGTGCTGCC CGTTTGTTGG

3901 ACAGCGCAGG TGGTGATCGT GTTGTTTTGG TATCACGAAA ACAAGATAGA ATCTGAGAAA

3961 AAAGAACCTT TTTATCACTT TACCCGCACC GATACTGGTC TTTCTTAGAT CATTTCGGGG

4021 CGAAGCAAAC AAGTCAGCAC GGCATACAAA TTTTGGGATA ATCCCTTAGT CAGTGCCTGT

4081 GTCTTTCTAA TGAAGATTGA CAATGGCGGC GGCGGCGGCG GTGGTAGCAA CCAACGCACT

4141 GGCCTTTGTT TTCATACTTT TCAATGAATT CAATTAGCTT AACCAACGAG GTTGTTGTTG

4201 ATGTTCTCAC GGAAGTCAGG AGAAGCTCCG ACGGGCGTGT AGGGTAAGTC GCAGTTCGGC

4261 AGCATTCTGG CAGTGTACAC CCAGCACACC GAGCGTTCTA GAACGCTGAA TCGTTGAAAC

4321 AGAGCAAATG ACTGAAGGGC TGTGTCTATT GCTGGGCTGT ACAGAATGAC GCATTTCGTG

4381 TGGCAATGTG TTTTCGAAAG ATTGAAGCAT TCTGATAGAG CCGTGTGTCA GGAATGTAAA

4441 TATTTAGATA CTGCAGCTAA CTACAAACTG AGAGAGCTTT CAGCTGGCAG GTGTCCCTAC

4501 TTGGTGGTCT TTTCTAGAAA GAATGTAGAA TTGACATGTT GTGATGATTT ACCAAAAACA

4561 ATTTGAGAAG CTAGAGAGTG AATCGTTCTA GCCACCGGAA CCATCTGGAA TGTTGAAGAT

4621 TTTTCTGGAA TATTAGTAGA CAAAAAGAAG GAGGTCTCAA CTCCGAGTAA ATTTGCAAAA

4681 TAGTTTTGAC TAAGTACAAA AAGGAAAGTT CCAAGATGTC ATACTCTACT AATGCTTCAA

4741 CTCACACATG ATCGCACACG ATTCTAGAAA CTCGTGGATG CATTAAAGCC AATGCCTTTT

4801 CACCGGTGAA TGACTGATAA GCAGATGCCT AGTTGGCAGT CTTGGTAGGC CACCATTCTC

4861 GATCAATGTC AAGTTCAGGA GCAAGGCTTC AAAGCCGACG TACAATGTGT GCAGCGGGCA

4921 TGGGCGGTTG TAGTGTCCGC GGTGCCGCGT TTAGCATGCG GCTTTGCCAC TTCCCTTATC

4981 AACAACACTG AGTGTGCACT TTCACGATAA TCGAAGCTGT CGTTTGTAGA TGCCCTCCCG

5041 ACACCCCGGG GGTCACGTGG GTCAGTACGA AAAATAAAAC TGATAGTAAG CAGCCGCAAG

5101 TGCTCCGTCA CTCGCTATCA GCCGGAATAG GTCAACAGAC TGCCAGCAGA TTATGCAACA

5161 TGTGGACGGT TACGCTCGTA CTAATCATTT GCTTCTATGC TTGCTTCTTT TCTACGCCTA

5221 CCCCCTCGGA CAACAGCATC GTGCAAGTCG GGATGCCCAG CACGTAAGTA TTTAGCACGC

5281 CGCGGACGAT ACCGATCTAT GGCTGACTGG GTTACGGGCA ATTACTAACG GCAATGTTTC

5341 TCCTGCTTCC TGCTCCTGCT CACTACAGCT AACAAATCCA GCAAGTACAC GTTCAAGCCC

5401 GGTTTCGTAT ATCAGTATGA TGTGGACAGC TATGTGCAGC TTCAGCAGTC GGACAAGGAG

5461 AACAAGCAGA CCACGCTGAA GGTCGACGGC AAGGTGGAGG TGTACGCCGG CGACAACTGC

5521 CAGTACACGC TGAAGGTGGT CTCGCTGACC TCGTACGCAC CGGACGGCAA GAAGACGGCG

5581 TTCGGGGCCG ACATCAGCAA GCCGGTCCAG TTCACGCTGT CGAACGATGA GCTGCTGCCC

5641 GAGATCTGTA CCGAAGCGGA CGATACGGAC TTTTCGCTGA ACGTGAAGCG TGGTTTGATT

5701 TCGCTGTTCC AGGTCGCGCA GGAGAAGAGC ACCGAGACGG ACGTGTTCGG CGTGTGCCAG

5761 ACGTCCTTCT CGAGCTACCC GTCCGGCGAT GCGACCGTCG TGGAGAAGGT GCGCGACCTG

5821 GGCAACTGTG CCTACCGCGA ATCGCTCTCG AACAGCTTCG TGACCCGCAT CGTCAACAGC

5881 AAGGCCGGCA TCAAGTCGAC GCCGCTGCTG CAAAGCTCGT ACAATGCCCA GCAAACGATC

5941 AAGGGTGGTC TGCTGTCGGC GGTCAAGCTG TCGGAGGAGT ACCAGTATCT GCCCTACCTG

6001 AAGGACAAGG TTGGCGTGAC CGCGAAGGTA ACGACGAAGC TGACGCTCAC CGGCAACAAG

6061 GCGGGTGCTG CGCCGGCCCT CGGCGCTGCG AGTGAGCCGC GCACGATCAT CTTCGAAAAT

6121 CCCGACCAGC AGCCGGCCGG CAATCTGGCC GTCATCAAGC AGGAGCTGAA ATCGACCGTC

6181 GAATCGTACA CGAACGGCAA CGTGGGCAAG AAGACGGCCA ACCTGTTCGT GGAGCTGATC

6241 CAGCTGATGC GCTACTCGAA GAAGGAGGAC CTGCTGACGC TGTACAACCA GGTGAAGGCG

6301 GGCAGCGTAC ACTCGAACAA GTCGCTCGCG CGCAAGGTGT ACCTGGACGC GCTGTTCCGC

6361 GTGGGCACGG GCGATGCGGT CGAAGCGATC ACGCAGCTGT ACAAGAACAA GGAGCTGACC

6421 GGTGCGCAGG AGCAGAAGCT CGCCTTCGTT TCGCTCACGC TGGTACAGTC GATGACGCAG

6481 GATGCGCTGA AGGCGGTCAA CAAGCTGCTG GACGGCAATC CGCCCCGCGA GGCGTACCTG

6541 AGCGTGGGTT CGCTGGTGAG CAAGTACTGC CAGAAGCACG GCTGCCAGTC GTCGGATGTG

6601 AAGGAAATCT CGAACAAGTT CGGCGCCAAG CTGGGCAAAT GCCAGTCGAC GTCGCGCGCC

6661 CAGGAGGACG TGATCGTGGC GGTGCTGAAG GGTGTCCGCA ACTCGGACAA TCTGGTCGCC

6721 CCGCTGCTCG ATAAGGTGAT CCAGTGCGCC GGTCCCGATG CTTCGTCGCG TGTGCGCGTG

6781 GCTGCCCTGC AAGCCTACCC GGCCGCTTCC TGCAACAAGA AGATCGTCAA CGCGGCGCTC

6841 AGCACGCTGA AGGACACGAA CGAAGACTCG GAAATTCGCA TCCACGCGTA CCTGTCGCTG

6901 GTCGAGTGCC CGTCGGCGAA CGTTGCCAAC GAGCTGAAGG CACTGCTGGA CGCGGAGAAG

6961 GTTTACCAGG TCGGTTCGTT CATCACGTCC CATCTGGCCA GCCTGCGGGC GTCGGTCGAT

7021 CCGACGCGGG ACGCTGCCCG GCAGCACTTT GGCAAGATCC GCACCTCCAA CAAGTTCCCG

7081 TTCGATGTGC GCCGCTACTC GTTCAACCGG GAGTTTTCGT ACGCGGTCGA GTCGCTCGGT

7141 GTCGGCGCCA GTGCCGAGAC GAGCGTGATC TACTCGCAGA AGAGCTTCCT GCCGAAGTCG

7201 GTTGGACTGA ACTTTACGGC CGAGCTGTTT GGCAATGGGC TGAACGTGTT CGAGCTCGAG

7261 GGACGCCAGG ACAATCTGGA ACGGCTGGTG GAGCATTACT TCGGACCGAA GGGCTTCTTC

7321 TCCGGCATGG ACATGCAGGC GGCGTACGAT CTCCTGGCCG AACAGTACCA GAAGCTGTCC

7381 GGCAAGGCGA AGGAGCGCTT CCGCCGCGGC ATCCGCGAGG ACATCCGTGC GCTGGCCCGC

7441 ACCGTCGATC TGCACAATGA CGCGCTGAAG GACTTCAACC TGGACGTTAC GATGAAGGTG

7501 TTCGGCTCGG AGCTGTTCTT CCTGAGCACG GGCGAGAACG TGCCGACCGA TCCGGAGCAG

7561 TTCCTGGACA AAGCGCTTGA ATGCTTCGAC AAGATGATCG AAGGGGCCAA GAAGTTCGAG

7621 CACACGTTCG AGCACCATGC CCTGTTCCTG GACACGGATC TGGTGTACAC TACCGCTCTC

7681 GGTCTGCCAC TGAAGCTTTC GGCGCAGGGT GCCGGTGTGG CGCGTGTCGA TGCCGCTCTC

7741 GAGCTGGACG TCAAGAGCCT GGTGAAGGAC TACAGCAACG CCAAGTTCAA CACGAAGTTC

7801 CAGCCAAGCG GCAGCTTCGA GGTGACCGGA ACGATGAGCG TGGACGCGTT CAACGTGATG

7861 ACGGGCATGC AGGTGGCCGT GTCGGGCCAC TCTTCGGGCG GTGCGGCCGT CAAGTTTGCG

7921 CTGCACGATG CCACCGCCTA CGATCTGACG GTCGACGCGC TCCAGGGCAA GCAGGAGCTC

7981 GTGTCGGTCA ACTTCCGCGA GGTGATCATT ACGCGCGAAC GGGGCAACCA GCTGATTACG

8041 CTGCCCGCCA AACGCCAGGA CTTTGGCTCG AAGTTCAGCG AGTGCTTCGA CAATCTGTAC

8101 AGCGTGATCG GTGTGACGGT GTGCGCCGCA CACAAGGTCG ACCAGGAGGA GCTGTTCGAG

8161 GTGTACCTGG AGGTGGAGCC GAAGTTCCAC TTCTCGGGCA AGTTCGACAA CTCGAACCCG

8221 CAGCACCTGC AGCTGTCCCT CAGCTTCGAT ACCCCCGGGT CGCAGACGAA GCGTCTGACG

8281 AACCTGCGAC TGGAGGGTGT GACCGCCGGC GAGCTGTACG TGAAGGCGAC GCTCGAGTCA

8341 CCGATCCGCA ACGTCGACTT CAAGCTGGGA GTAAACAACA ACGACAAGGA GGTGGCCCTG

8401 TACGCCGTTG CGCACAATGG CGTGGAGGAG TACCTGGCCA AGATTGGCTT CCAGAAGGGT

8461 GCGTCCGGTG GGCGCGATGA GTACGTGCCG ATCTTCACCA TCCGCTCGCC GAACGGCGAC

8521 GCCCAGGTGT CCAAGTTTGT GCAGACGACC GGCAAGATCG TGGTGGAGAG CCTGGATGGC

8581 GGCAAGCGCA AGTACAACCT GGAGAACATC GAGTGGACCA GCCCGTACGC CCCGAAGACG

8641 ACCATCAACG GGCACGTCGT GTCGAACGGC GAGCGTAGCT TCGATGCCAA CGTGGACGTG

8701 AACGTCGGTG ACGTGAAGAA CAACGTGGTG GGCCATCTGG ACTTTGATCT GAAGCACGTC

8761 AAGCTCGACC TGGAGAAGAA GACGCCGAGC GATGCGAACA AAAACTTCAA GGTCAACCTG

8821 GAGGTGGCGT ACACGGACAA CTCGTTCAAG AACCTGTTCT CGTTCGCCAG CGGCAAGGAC

8881 TTCAACAATC CCTCGAACAA GTACGAGCTG AGCCAGTACG CTGAGTTTGA GCTCAAGCCC

8941 GAGGCGCAGG GACTGGAGTC GCTGAAGCTG GAGAACAAGC TGCAGCTGCC GAAGCAGCTG

9001 ATCCGACTGG ACTTTGCCAC GAACAAGAAC AAGTTCTACC TGGACGGTGA GTACGGGTAC

9061 GACAAGTACA AGATTGCGGC GAACGTGGAT GCGAAGTACA ACGAGAAGAC GCCCGGGGAT

9121 TACGATGTGC AGCTGGGCGG TTCGCTCAAC AAGCACTTCT TCAAGTTCTT CTCCAAGCGC

9181 ATCGTGGAGG CCAACAAGAG CCGCTTCAGC AACAAGCTGA CGGCCAGCAC CGGTACCAAG

9241 TTCGAGCTGA ACGGTGCCGT CACGAACCGC TTCACCAGCC AGGATGGTGA GCTTAACCTC

9301 GAGGGATCTC TCATCGCTGT CGAGAAGGCT TCTCCTTACA AGTGAGTAGG AAACGCAATG

9361 ATTATTGGCA GGGCAATGAT ATCACTAATT GGTTGTGCTG TTCAGGCTTT CTTTGACAGT

9421 TCAGTTCTCG GCCGCGAACG TCCTCTCCAA CGCCAAGGTG CTGGTGGATA AGGAAGAGTT

9481 CGCTACGTAC GACTTCAAGC TAGAGCGGGG ACAGGATCCG AACGGCAAAT TCACGGTAAG

9541 TCTCCGCAAA CAACTCTCAA CTCGTTTCAG TTTTCCTTAC GCCACATGTT TCATTGACTC

9601 TCCCAACAGT TTGTGGTGAA GGACTTCTTC AACGGAAATG GTGAACTAAA GTCGGCCAAG

9661 GGTCAGGGTG AGCTGTTCGC CCTGGTCACG TTCGTCAAGC AGGACCGCAA GGTGAAGCTG

9721 GACAGCAAGT TCAAGGTGAA CGCACCGGTG TACGATGTGG CTGCCGATTT CTACTACGAC

9781 TTTGAGAAGG ACAACAGCAA GAAGGTACGC TTCGAAACCA AGAACAAGGT CACCCAGACC

9841 AGCTTCGACA GCAAGAACAA GGTGGAGGTG TTCTCGGAGA AGTACGAGCT GAACGTACAG

9901 AGCCAGGGCA ACCCGAAACC GGTCGATGGC AAGTTCAACG TCAAGGTCAG CCTGCTGCTC

9961 CCGACCGGAC GCCAGTTCGG CGGCGAGTTC CAGCGCGATG CCTCGACCAA GGACGAGAAG

10021 CGCTCCGGCA AGATGGCGGC CAGCGTGTAC GACAAGCAGC CCGGTGGCAA GAAGCGCTCG

10081 GTCGAGTGGG CCGGCGAGCT GAAGGATATG GACGTGAAGA CGAAGTTCTT CGACGCGGTG

10141 CACAATGTCA AGTACTCCGA TCTCGAAGGC AAGGATGTGG TGCTGGACGT GACGCTAAAG

10201 CATGCGCCGG CTGGTAGCTA CAAGTCGGCC GCCGGTAGCT TGAAGGTTTC GGGCAGCTTG

10261 CTTCCCCAGG TGACGGAACT GAGCGTGGTC GTCGATGAGT ACTGTGAGCA TCACGCCAAG

10321 TACCACGTCA ACGGCAAGTA CGGGGCGGAC TTTACGGCTG CACTGGTCGG TGGGTACCAC

10381 ACCGGAGGGC ACGGCAAGCC GGCCACCCAC GACCTGAAGG TTGACGTGGG TGCTCCATCG

10441 TACAAGGTGG GCGTATCGTC CAGCGGCAAG TACCTGCAGC CAGAGTCGGA CGACGGTGTG

10501 TACGAGCTCG ACTACAGCGG ATCGGTCGAC TTCAACGGCA AGAGTGCGTC GGTGTCGACG

10561 CAGGCCAAGG GCAACTACAA CCGTGGCAAT GGAAAGCTGA ACCTGAACCT CCCGAACGTG

10621 GACCCGATCG CGGCCGAAGG TTCGTACACG TACGACGCGA AGGAGGAGGG ACCGTTCCAG

10681 ACGAACGGCG CCCTGAAGGT GTCGTACGGT GCGGGCAAGA ACTTTGAGTT CACCGGCACC

10741 GCCAAGGCCC CGTCGATGGA CGATATCCAG GTGCACGCGA CGCTCAAGTC GGAGTTCGAG

10801 AACGTGCGCA GCGTCGACCT GACCTTCAAG CACGCCAAGT CGAGCGACAG TGCGTACAAC

10861 ACGAAGCTGC AGCTGACCGC CGACGACAAG AAGTTCAGCG TGGAGAACGC GGTGGTCGTG

10921 TCGGAGACGA ACCCGTCCGT TGACTTTACG CTCGGCTATC CGGGCAAGAC GGTGAAGGTG

10981 TCGGGCAGCT ACAAGGCGCT CGGCCACAGC GCATTCAAGG CGGACGCCAA GGTGCAGAAC

11041 CTGGCCAACT TCGACATGGA AGCGAACGTG GAGGCCAACT TTGACAGCTA CCAGACGTTC

11101 TACGTGAAGC TGTACGGCGA TGCGCCGATG CTGAACACGA ACAAGTTCTC GGTGGAGGTG

11161 AACGCGAAGC CGGGCTCGAA CGGCAAGGGC GTTAACTTCC GCGCGTCCGA GGGCGGTAAG

11221 GACATACTGA GCGGCTTTGC CGACTACTCG GTGAAGGAGC AGGGCAAGGC GATGGTCATC

11281 GAGGGCCAGG GCAACGTGAA GCTGTACGAC AAGCAGCAGA CCGCCACCTT CAAGCTGATC

11341 CGCGAGAAGC TGTCCGAGTC GGGCATGTCG GCCACGCTGA CGGCCTCGGT GGGCAAGTTC

11401 ACCGTGCTGC ACGAGTCCCG CGTCCAGCCG AACGATTTCC GCGTCAAGAC GAGCGTGTGC

11461 GACGAGAAGA AGAAGTGCAC CAAGCTGGAG CTGCTCTCGA AGCTGGAGCG CGCGGCCGGT

11521 GCGTTCAAGC ACGAGGCGCT GGTGTCGGTG GAGGTGCAGC AGATGGGCTA CGAGCACGAG

11581 TTCGGCCTGT CGGCCAAGAC GAGCGCCAAC GGGCTGAAGT TCGACCACAC CACGGACGTG

11641 CAGCTGAAGG AGAAGAACCA GCCGAAGTAC CAGTACCTGT TCTACGTGCA TCCGACCTCG

11701 GCCGGAGCGA GCCTAATACT GCCGACGCGC ACGGTCGCCG TGGAGGGTGT GCTGAACCTG

11761 CCCAAGGACA AGTTCGGACC GTTCGACGGC AGCGTGTCGT TCTATCTGGA CAAGAAGAAC

11821 GAGCCGGACA ACAAGGCCAC GTTTGCGGTG CGCGGCGAAA CGAAGATGCT CGGCTCGACC

11881 GGCGTCAGTG CGAACGGTGC GCTGACGTTC TCGCATCCGA CGATCAAGGC GCTGGCCATC

11941 CGGGCCAAGG GTACGCTCGA TGGGGACAAG CAGACGGCGG ACGGATCGGT TGAGTTTGAC

12001 GTGTTCAAGA AGGCCGGCGA CAAGATCGTT GCCAAGGTGC GCTACGCCAA CAGCGACCAG

12061 TCGTTCAAGG GCTTCAACAT CACGACCGAG GCAAGCGTGT CAAGCAAGGG CCTGGGGCTG

12121 AAGTGTGGCT TCAGCGGCCA CTCGGCCATG TCGCTCGCAA CCCGCCAGGC CAGTGCGGCC

12181 GGTTCGCTCA CGCTGCCGTT CGAGGGCTAC ACGTTTGGCT CGTACTTCTT CGGCAGTCCG

12241 GAATCGTTCG ACTTCCTGCT GACCCGCTTC GGGGACGACT TTGTGCGCGC GCACGGCACG

12301 TACGATGCGA AGAAGTACCG CGGCGACCTC ACCTCCACCT TCAAGTTCCT GCCCAACCGG

12361 CCGGTGGTGT TCGAGTCGCA GCTGAACGGT CTGTCGTCGG CCAAGTTTGC CTTCAAGCAG

12421 GACCAGTTCT TCAGCGCGGA CGGTACGTTC GGCGTGGACA AGGCGATGGT GCTGAAGGTG

12481 ATGGGCGAGG GTAAGCCACT GCTGAACGCG AAGGTAACGC TGGACGCCAG CCACTTCCTA

12541 TCGACCGAGT ACAATGTGGA CGAGGCGAAC GCGAAGGCGT TCCTGGTGTC GCTCAAGAAC

12601 CAGCTGAACG CCGACTTCGA GGTGACGCGG GCGGACGTCA GCCAACGCTA CGCCAAGCTG

12661 GCCGAGGAGC TGAACAAGCT GTCGACGAAC CTGGTGAGCG CGCTGCCCGA GTTCGGCAAG

12721 TTCCAGGAAA GCTACGCCAA GCAGCTGCAG AAGCTGCAGG AGGACATCAT GAGCGATCCG

12781 GCCCTGGCTG AGTTCGTGAA GGCTGCCACC AAGATCTTCC AGCAGGTGAG CGAAGTGTTC

12841 GGCCAGCTGT CGCAGGTGTA CGTGGAAAGC TTCCGCAAGA TGTCCGCCCT GGTGAACGAC

12901 GTCGTCGCCC AGCTGATGGA AACGTTCAAC ACGAAGGTAC TGCCGGCGCT GAAGGAGCTG

12961 TCCACCAAGG TGGAGGCGAT CTTCTTCAAC GTGTACGAGG AGACGGTCAA GCTGGTGGTG

13021 GCCGTGTTCG AGCGCACCGT CAAGGCGCTG AAGGTGTTCG AGGAGGACTT CAACAAGATC

13081 GCCACGAGCG TGTCGGAGCT GTTCCGCACG TTCGCGCAAA CGTTCAGCAA GGCCGTCCAG

13141 GTGCTGGAGA AGGAGCTGAA GGAGCTGTAC AAGCTGGTGC AGGAGTACTT CGACACGTTC

13201 GACGAGTTCA AGGCGGTGAA GGAAACGTTC AAGGAGTACT TCGACGGGTT CGACCGGTAC

13261 GCGTACCAGC TGCTGAAGGA GCTGCTGTCG CTGGTCGAGG CGGTCTACCC GATGCCGGAG

13321 GTGACCGAGC TGACCACCGC GATCAACAAG TACATCACCA GCAAGCTGGA CAACAAGCCG

13381 GTCAACGATG TGGAGGAGCT GAAGACGCTG TTCGTGAGCC TCGTCAAGGT GCTGAACCAG

13441 GCGGTGGAGC GCCTCATCGC GGGAGTGAAC GTGCAGCTGT CCGAGCCGAC GTTCGGCAGC

13501 GACAGCTTCA CCTCGTTCGT CACCTTCAAG TTCCTGCCGT ACGTGTCGAG CATCCAGTTC

13561 AGCCCGTGGA ACTTTGTGCG CAACGAGAAG TTCTACTCGG TGCGCGACCT GATCCACCAG

13621 CTGCGCCTGT ACGCGTTCAA TCCGTTTGCG CGCGTGCCGA TGTTCCACAT GCACGCCCAG

13681 CTCGGCGACG GTGGCCACTT CTTCACGTTC GACGACAAGC ACTTTACGTT CGCGGGCAGC

13741 TGCTCGTACC TGCTCGCGTC CGACCTGGTC GACGGTAACT TCAGCATCGT GGCGGACATG

13801 GACGGCGGAC GCCTGAAGTC GGTGACGCTC GTGGACAAGG ACTCGACGGT CGAGCTGACC

13861 GCCAAGGCGG TGGTGAAGTA CAACGGCAAG GAGACGGATC TGCCCATCCA CCAGAAGGAT

13921 GTGTACGTGT TCCGCAAGTA CTACACGGTC ACGGTCGGCA CCAAGTACGG TGCGCAGGTG

13981 ATGTGTACGA CCGACCTGAA GATCTGTCAC TTCTTCGTGT CCGGGTTCTA CTTCGGGCGG

14041 CTGCGCGGTC TGCTCGGCAA CGGCAATTAC GAGCCGTACG ACGATCTGGC GGTGCCGAAC

14101 GGCAAGATCA CCGAGGTGTC GACGGACTTT GCGAACAGCT ACAAGACGAG CCAGGCGTGT

14161 GCGGCAGTCG CCGACCACGG TCACGATTCG CACGACCACT CCAGCCCGAC CTGTGCCAAG

14221 TTCTTCGGCT CGGAGTCGTC CCTGAAGCTG TGCTCGTACC TGCGCGACCA GACCGGCTAC

14281 AAGGAGGCGT GCAACCATGC GGCGCACGAT GCGGGCGAGA AGGCGGACGA GGCCGCTTGC

14341 GGCATTGCCC GGCTGTACGT GTCCGCCTGC ACGCTGTACG GCATCCCGAC CGTGCTGCCC

14401 AGCCAGTGCG AGAAGTGCTC GACCGACGAG GGCCGCTCGG TCGATCTGGG CGACTGGTAC

14461 TCGGTGAAGG CGCCGCAGAA GAAGGCCGAC GTGGTGGTGG TGGTCGACAC GTCGCTCGGC

14521 ACGCTGCTCG GCGAGCTGGT GCAGTCCACG ATCAACGATC TGCGCAAGGA GCTGAAGGCG

14581 ACCGGCATCA GCGACGTGAA CGTTGCGGTG ATCGGCTACA GCAAGACGGA CAAGTACACG

14641 AGCCTGTTCT CGAACGGCGG CAAACTGGAC TACACGGGCA AGCTCGGCCA GGCCGACGTC

14701 AGCAGCGGAC CGAAGCAGTG CCGCGGGCTG GTGACGGGCA TCGAGTCGGT GGACGCGTTC

14761 CTGCAGCTGC TGCAGAAGAT GGGCGAACAG TCGCGCGAGA ACTTGGGCGC TACGACCGAG

14821 GTGTACGCGC TGCGCCGTGC CTTCAGCTAT CCGTTCCGTG CCTCCGCCAG CAAGTCGGTG

14881 CTGGTCTTCC GTTCCGATTC GTTCGAAATC GCTCAACCG

//

**Supplemental File 1C: *Lp* gene drive plasmid**

LOCUS Lp-GD 13956 bp ds-DNA circular

DEFINITION synthetic circular DNA

ACCESSION .

VERSION .

KEYWORDS .

SOURCE synthetic DNA construct

ORGANISM synthetic DNA construct

REFERENCE 1 (bases 1 to 13956)

AUTHORS Emily GREEN

TITLE Direct Submission

JOURNAL Exported Wednesday, Sep 18, 2019 from SnapGene 4.3.11

https://www.snapgene.com

FEATURES Location/Qualifiers

terminator 268..295

/label=rrnB T2 terminator

/note="transcription terminator T2 from the E. coli rrnB

gene"

terminator 387..473

/dnas_title="Escherichia coli rrnB"

/gene="Escherichia coli rrnB"

/label=rrnB T1 terminator

/note="transcription terminator T1 from the E. coli rrnB

gene"

primer_bind 537..553

/label=M13 fwd

/note="common sequencing primer, one of multiple similar

variants"

misc_feature 771..773

/label=lac promoter

/note="lac promoter"

misc_feature 774..2326

/label=Lp promoter

/note="Lp promoter"

misc_feature 2341..2665

/label=U6 promoter (ag)

/note="U6 promoter (ag)"

misc_feature 2671..2740

/label=tRNA-gly

/note="tRNA-gly (missing terminal A, which is added when

cloning guide linker)"

misc_feature 2741..2848

/label=gRNA template

/note="gRNA template"

misc_feature 2854..2923

/label=tRNA-gly

/note="tRNA-gly (missing terminal A, which is added when

cloning guide linker)"

misc_feature 2924..3031

/label=gRNA template

/note="gRNA template"

misc_feature 3037..3106

/label=tRNA-gly

/note="tRNA-gly (missing terminal A, which is added when

cloning guide linker)"

misc_feature 3107..3214

/label=gRNA template

/note="gRNA template"

misc_feature 3220..3289

/label=tRNA-gly

/note="tRNA-gly (missing terminal A, which is added when

cloning guide linker)"

misc_feature 3290..3397

/label=gRNA template

/note="gRNA template %22misc_feature 3403..3473

/label=tRNA-gly /note=%22tRNA-gly"

misc_feature 4556..4624

/label=3xFLAG

/note="3xFLAG"

misc_feature 4625..4675

/label=NLS

/note="NLS"

misc_feature 4676..8776

/label=Cas9

/note="Cas9"

misc_feature 9870..9916

/label=3x Pax6 binding sites

/note="3x Pax6 binding sites"

/label=TATA

/note="TATA"

CDS 10115..10848

/label=DsRed2nls

polyA_signal 10856..11093

/label=SV40

promoter complement(12198..12216)

/label=T7 promoter

/note="promoter for bacteriophage T7 RNA polymerase"

primer_bind complement(12221..12237)

/label=M13 rev

/note="common sequencing primer, one of multiple similar

variants"

CDS 12350..13159

/dnas_title="aph(3')-Ia"

/codon_start=1

/gene="aph(3')-Ia"

/product="aminoglycoside phosphotransferase"

/label=KanR

/note="confers resistance to kanamycin in bacteria or

G418 (Geneticin(R)) in eukaryotes"

rep_origin 13306..13894

/direction=RIGHT

/label=ori

/note="high-copy-number ColE1/pMB1/pBR322/pUC origin of

replication"

misc_feature complement(11094..12189)

/note="3%82%C4%F4flk homology arm"

misc_feature complement(8828..9869)

/note="zpg terminator"

misc_feature complement(3481..4554)

/note="zpg promoter"

misc_feature 11145..11164

/note="gRNA4target site present by mistake%E2%80%9C

misc_feature 2742..2761 /note=%22gRNA1"

misc_feature 2925..2944

/note="gRNA2"

misc_feature 3108..3127

/note="gRNA3"

misc_feature 3291..3310

/note="gRNA4"

misc_feature 2742..2761

/note="gRNA1"

source 1..13956

/dnas_title="pENTR-Lp5'-sgRNAx4(lp)

-ZpgCas9Zpgt-DsRed-Lp5'"

ORIGIN

1 ctttcctgcg ttatcccctg attctgtgga taaccgtatt accgcctttg agtgagctga

61 taccgctcgc cgcagccgaa cgaccgagcg cagcgagtca gtgagcgagg aagcggaaga

121 gcgcccaata cgcaaaccgc ctctccccgc gcgttggccg attcattaat gcagctggca

181 cgacaggttt cccgactgga aagcgggcag tgagcgcaac gcaattaata cgcgtaccgc

241 tagccaggaa gagtttgtag aaacgcaaaa aggccatccg tcaggatggc cttctgctta

301 gtttgatgcc tggcagttta tggcgggcgt cctgcccgcc accctccggg ccgttgcttc

361 acaacgttca aatccgctcc cggcggattt gtcctactca ggagagcgtt caccgacaaa

421 caacagataa aacgaaaggc ccagtcttcc gactgagcct ttcgttttat ttgatgcctg

481 gcagttccct actctcgcgt taacgctagc atggatgttt tcccagtcac gacgttgtaa

541 aacgacggcc agtcttaagc tcgggcccct acaggtcact aataccatct aagtagttga

601 ttcatagtga ctggatatgt tgtgttttac agtattatgt agtctgtttt ttatgcaaaa

661 tctaatttaa tatattgata tttatatcat tttacgtttc tcgttcaact tttctataca

721 aagttggtac cgggcccccc gctagcgtcg acggtatcga taagcttgat cggatccaac

781 agttcattcc cgattgaggg attttattcc ccgggggcct tttcaaacgg cttaatataa

841 gcaattaata gtattttttc tttcaggtta gtttactgta atggtgtaat tgtcatctta

901 cacctccgtc tgataagaga ttacgaagct cagtatgatg aaataaataa gataaattta

961 tttaaaaaag aacaattgct atgagagtga aatacaacag tggcgttcac aatattcgaa

1021 aaacaataaa attaaaaaaa aaaaacaaga aaacattcac aaacatatca atctgctttc

1081 atcgacacca aactgctagc ctccccagtc taaccgcggt ggggacgttt aattgccttt

1141 gttctcgcac ccggtcaaac atacacttcg gaccttgctc cgaaccccac tgtaatccct

1201 agctcgtcat catcattgcc ggcatcatgc taagcgtgca ttattttcac aacttagcgt

1261 aatgctagcg tgcgctagca acaaactcgc ccgcagactc gtcacagcac cggtacgatc

1321 gatcgtttac cgttcccttt cccgatcggg ttggctgcga tatccgtgtc cgggtagaaa

1381 acttcccctt ttacacatac acatacactc acatacacac agagctgaat agcaacttac

1441 cttatctgtt cgtcatcgct cggccggatc tggatgaatc ttcgcaccga taaccatgtg

1501 gatctacgac ctccgcttgg ctgtctctct gctcatgtgt atgtctgtgt gtgtgtgtgt

1561 gtgtgtgtgt gtgtgtgtgt atgtgagctt cttccctcaa atccctcgat ctcgctgtgc

1621 caacaatcaa acgtgcaagt gcaaacattg caccccattg attatacacc aacaccaacc

1681 aattcccctt gcggaggcat cactgtgctc ggcagcatgt ttaccgcaga tctacagaga

1741 acttcaattg aggtcctttc cacccccagc cctcaaccgg caatccggca gccactggat

1801 catcagcgaa agagagagag agcagagcag aacagaggtg accaactgtg gtatcgcttc

1861 ccgcgcgccg gtgtgttggt gtccatttcg gtgatcgcga tcccggccgc ttccagcacc

1921 gtccaccgat cagtcaccaa aacgctctcc aaacccctta tcagcaccgt tcgctgatgt

1981 gaacccccgt tcaaacccca aatgcagtgt ttgtattgct gtgtgtatgt gtgcgtgcgt

2041 gtgtgtggaa attctataaa agtaggcaac cgtggccggg atccgttatt cccgtcctga

2101 ggcccgcccg ggatcgctgg tgacgacaga cgagccgctg tgtgacgtac gtagtgcccg

2161 atcggtaaag agtgaaccgt cttctctgca gtgttggaga gaacggtttc atctttttcg

2221 cccacacccc ccccggttac attccatgtt gagctgtaag gtctagtgaa catttcctga

2281 gtgtggaaag tgtggtttag tgcgtgagag tgcacggaca cgacacggaa cgatgtgggt

2341 ttatccacat atacactgaa gcggtttggc tcttggttat tctagaaaac tctgtttttg

2401 aattaatcat aggaaaactg tttattcttt tttgatattt caacgtatta caatgaatag

2461 taacttttga ttaaaaaaga gatgaagcaa tacaagaaat gttgaaaagt ttatgaaaca

2521 tcacattagc gtgagttacg gcaggatcaa aaccttataa cagtcacact caggcaaaaa

2581 atccttcttg aatatccttt atgcatcgct agagcaagga ttgaaagcgc aaagtatata

2641 tacaaccttt tttcccctcg tccttgtaaa gcatcggtgg ttcagtggta gaatgctcgc

2701 ctgccacgcg ggcggcccgg gttcgattcc cggccgatgc acacggaacg atgtgggtcc

2761 tgtttcagag ctatgctgga aacagcatag caagttgaaa taaggctagt ccgttatcaa

2821 cttgaaaaag tggcaccgag tcggtgctag agagcatcgg tggttcagtg gtagaatgct

2881 cgcctgccac gcgggcggcc cgggttcgat tcccggccga tgcagaggct gctctggagc

2941 ttccgtttca gagctatgct ggaaacagca tagcaagttg aaataaggct agtccgttat

3001 caacttgaaa aagtggcacc gagtcggtgc tccatagcat cggtggttca gtggtagaat

3061 gctcgcctgc cacgcgggcg gcccgggttc gattcccggc cgatgcactg attcaaagtg

3121 tgtccgcgtt tcagagctat gctggaaaca gcatagcaag ttgaaataag gctagtccgt

3181 tatcaacttg aaaaagtggc accgagtcgg tgctggaaag catcggtggt tcagtggtag

3241 aatgctcgcc tgccacgcgg gcggcccggg ttcgattccc ggccgatgca gcgtgaaata

3301 tcgtcaggga gtttcagagc tatgctggaa acagcatagc aagttgaaat aaggctagtc

3361 cgttatcaac ttgaaaaagt ggcaccgagt cggtgctacg aagcatcggt ggttcagtgg

3421 tagaatgctc gcctgccacg cgggcggccc gggttcgatt cccggccgat gcattttttt

3481 attcgctggc ggtggggaca gctccggctg tggctgttct tgcgagtcct cttcctgcgg

3541 cacatccctc tcgtcgacca gttcagtttg ctgagcgtaa gcctgctgct gttcgtcctg

3601 catcatcggg accatttgta tgggccatcc gccaccacca ccatcaccac cgccgtccat

3661 ttctaggggc atacccatca gcatctccgc gggcgccatt ggcggtggtg ccaaggtgcc

3721 attcgtttgt tgctgaaagc aaaagaaagc aaattagtgt tgtttctgct gcacacgata

3781 gttttcgttt cttgccgcta gacacaaaca acactgcatc tggagggaga aatttgacgc

3841 ctagctgtat aacttacctc aaagttattg tccatcgtgg tataatggat ctatcgagcc

3901 cggttacact acacaaagca agattatgcg acaaaatcac agcgaaaact agtaattttc

3961 atctatcgaa agcggccgag cagagagttg tttggtattg caacttgaca ttctgctgcg

4021 ggataaaccg cgacgggcta ccatggcgca cctgtcagat ggctgtcaaa tttggcccgg

4081 tttgcgatat ggagtgggtg aaattatatc ccactcgctg atcgtgaaaa tagacacctg

4141 aaaacaataa ttgttgtgtt aattttacat tttgaagaac agcacaagtt ttgctgacaa

4201 tatttaatta cgtttcgtta tcaacggcac ggaaagatta tctcgctgat tatccctctc

4261 gctctctctg tctatcatgt cctggtcgtt ctcgcgtcac cccggataat cgagagacgc

4321 catttttaat ttgaactact acaccgacaa gcatgccgtg agctctttca agttcttctg

4381 tccgaccaaa gaaacagaga ataccacccg gacagtgccc ggagtgatcg atccatagaa

4441 aatcgcccat catgtgccac tgaggcgaac cggcgtagct tgttccgaat ttccaagtgc

4501 ttccccgtaa catccgcata taacaaacag cccaacaaca aatacagcat cgagaatgga

4561 ctataaggac cacgacggag actacaagga tcatgatatt gattacaaag acgatgacga

4621 taagatggcc ccaaagaaga agcggaaggt cggtatccac ggagtcccag cagccgacaa

4681 gaagtacagc atcggcctgg acatcggcac caactctgtg ggctgggccg tgatcaccga

4741 cgagtacaag gtgcccagca agaaattcaa ggtgctgggc aacaccgacc ggcacagcat

4801 caagaagaac ctgatcggag ccctgctgtt cgacagcggc gaaacagccg aggccacccg

4861 gctgaagaga accgccagaa gaagatacac cagacggaag aaccggatct gctatctgca

4921 agagatcttc agcaacgaga tggccaaggt ggacgacagc ttcttccaca gactggaaga

4981 gtccttcctg gtggaagagg ataagaagca cgagcggcac cccatcttcg gcaacatcgt

5041 ggacgaggtg gcctaccacg agaagtaccc caccatctac cacctgagaa agaaactggt

5101 ggacagcacc gacaaggccg acctgcggct gatctatctg gccctggccc acatgatcaa

5161 gttccggggc cacttcctga tcgagggcga cctgaacccc gacaacagcg acgtggacaa

5221 gctgttcatc cagctggtgc agacctacaa ccagctgttc gaggaaaacc ccatcaacgc

5281 cagcggcgtg gacgccaagg ccatcctgtc tgccagactg agcaagagca gacggctgga

5341 aaatctgatc gcccagctgc ccggcgagaa gaagaatggc ctgttcggaa acctgattgc

5401 cctgagcctg ggcctgaccc ccaacttcaa gagcaacttc gacctggccg aggatgccaa

5461 actgcagctg agcaaggaca cctacgacga cgacctggac aacctgctgg cccagatcgg

5521 cgaccagtac gccgacctgt ttctggccgc caagaacctg tccgacgcca tcctgctgag

5581 cgacatcctg agagtgaaca ccgagatcac caaggccccc ctgagcgcct ctatgatcaa

5641 gagatacgac gagcaccacc aggacctgac cctgctgaaa gctctcgtgc ggcagcagct

5701 gcctgagaag tacaaagaga ttttcttcga ccagagcaag aacggctacg ccggctacat

5761 tgacggcgga gccagccagg aagagttcta caagttcatc aagcccatcc tggaaaagat

5821 ggacggcacc gaggaactgc tcgtgaagct gaacagagag gacctgctgc ggaagcagcg

5881 gaccttcgac aacggcagca tcccccacca gatccacctg ggagagctgc acgccattct

5941 gcggcggcag gaagattttt acccattcct gaaggacaac cgggaaaaga tcgagaagat

6001 cctgaccttc cgcatcccct actacgtggg ccctctggcc aggggaaaca gcagattcgc

6061 ctggatgacc agaaagagcg aggaaaccat caccccctgg aacttcgagg aagtggtgga

6121 caagggcgct tccgcccaga gcttcatcga gcggatgacc aacttcgata agaacctgcc

6181 caacgagaag gtgctgccca agcacagcct gctgtacgag tacttcaccg tgtataacga

6241 gctgaccaaa gtgaaatacg tgaccgaggg aatgagaaag cccgccttcc tgagcggcga

6301 gcagaaaaag gccatcgtgg acctgctgtt caagaccaac cggaaagtga ccgtgaagca

6361 gctgaaagag gactacttca agaaaatcga gtgcttcgac tccgtggaaa tctccggcgt

6421 ggaagatcgg ttcaacgcct ccctgggcac ataccacgat ctgctgaaaa ttatcaagga

6481 caaggacttc ctggacaatg aggaaaacga ggacattctg gaagatatcg tgctgaccct

6541 gacactgttt gaggacagag agatgatcga ggaacggctg aaaacctatg cccacctgtt

6601 cgacgacaaa gtgatgaagc agctgaagcg gcggagatac accggctggg gcaggctgag

6661 ccggaagctg atcaacggca tccgggacaa gcagtccggc aagacaatcc tggatttcct

6721 gaagtccgac ggcttcgcca acagaaactt catgcagctg atccacgacg acagcctgac

6781 ctttaaagag gacatccaga aagcccaggt gtccggccag ggcgatagcc tgcacgagca

6841 cattgccaat ctggccggca gccccgccat taagaagggc atcctgcaga cagtgaaggt

6901 ggtggacgag ctcgtgaaag tgatgggccg gcacaagccc gagaacatcg tgatcgaaat

6961 ggccagagag aaccagacca cccagaaggg acagaagaac agccgcgaga gaatgaagcg

7021 gatcgaagag ggcatcaaag agctgggcag ccagatcctg aaagaacacc ccgtggaaaa

7081 cacccagctg cagaacgaga agctgtacct gtactacctg cagaatgggc gggatatgta

7141 cgtggaccag gaactggaca tcaaccggct gtccgactac gatgtggacc atatcgtgcc

7201 tcagagcttt ctggccgacg actccatcga caacaaggtg ctgaccagaa gcgacaagaa

7261 ccggggcaag agcgacaacg tgccctccga agaggtcgtg aagaagatga agaactactg

7321 gcggcagctg ctgaacgcca agctgattac ccagagaaag ttcgacaatc tgaccaaggc

7381 cgagagaggc ggcctgagcg aactggataa ggccggcttc atcaagagac agctggtgga

7441 aacccggcag atcacaaagc acgtggcaca gatcctggac tcccggatga acactaagta

7501 cgacgagaat gacaagctga tccgggaagt gaaagtgatc accctgaagt ccaagctggt

7561 gtccgatttc cggaaggatt tccagtttta caaagtgcgc gagatcaaca actaccacca

7621 cgcccacgac gcctacctga acgccgtcgt gggaaccgcc ctgatcaaaa agtaccctgc

7681 gctggaaagc gagttcgtgt acggcgacta caaggtgtac gacgtgcgga agatgatcgc

7741 caagagcgag caggaaatcg gcaaggctac cgccaagtac ttcttctaca gcaacatcat

7801 gaactttttc aagaccgaga ttaccctggc caacggcgag atccggaagg cgcctctgat

7861 cgagacaaac ggcgaaaccg gggagatcgt gtgggataag ggccgggatt ttgccaccgt

7921 gcggaaagtg ctgagcatgc cccaagtgaa tatcgtgaaa aagaccgagg tgcagacagg

7981 cggcttcagc aaagagtcta tcctgcccaa gaggaacagc gataagctga tcgccagaaa

8041 gaaggactgg gaccctaaga agtacggcgg cttcgacagc cccaccgtgg cctattctgt

8101 gctggtggtg gccaaagtgg aaaagggcaa gtccaagaaa ctgaagagtg tgaaagagct

8161 gctggggatc accatcatgg aaagaagcag cttcgagaag aatcccatcg actttctgga

8221 agccaagggc tacaaagaag tgaaaaagga cctgatcatc aagctgccta agtactccct

8281 gttcgagctg gaaaacggcc ggaagagaat gctggcctct gccggcgaac tgcagaaggg

8341 aaacgaactg gccctgccct ccaaatatgt gaacttcctg tacctggcca gccactatga

8401 gaagctgaag ggctcccccg aggataatga gcagaaacag ctgtttgtgg aacagcacaa

8461 gcactacctg gacgagatca tcgagcagat cagcgagttc tccaagagag tgatcctggc

8521 cgacgctaat ctggacaaag tgctgtccgc ctacaacaag caccgggata agcccatcag

8581 agagcaggcc gagaatatca tccacctgtt taccctgacc aatctgggag cccctgccgc

8641 cttcaagtac tttgacacca ccatcgaccg gaagaggtac accagcacca aagaggtgct

8701 ggacgccacc ctgatccacc agagcatcac cggcctgtac gagacacgga tcgacctgtc

8761 tcagctggga ggcgacaaaa ggccggcggc cacgaaaaag gccggccagg caaaaagaaa

8821 aagtaagaat tgaggacggc gagaagtaat catatgtccg cattttgcgc aaaccaggcg

8881 cttagacaat ttgcgcgtaa gcacattcga aatgtgaaaa gctgaaagca gtggtttcgc

8941 cagcccgagt tcagcgaaac ggattccttc caagtgtttg cattcctggc ggagtgttcc

9001 tcccaaaatg cactcaccct gcgtgcagtg ccaaatcgtg agtttcttaa ttttttcata

9061 ttgtttatta cctaccaact aaagttgttg ttatatattg cgttttacgt acgacaaata

9121 agttcgtatt cagaaatatt tgcgataaga gagaactcat ttgcgatgaa tctcattgta

9181 tttagctaag tgccttgata agtaagcgga acagcaggaa tatgacactc cttgggaaat

9241 acatgtaagc gtctgtgaat tagatatata tacacgcaac caaacggtcc atggttgatt

9301 taagcactgc ctgttgtcga acattgctat aagcaaaata aagaagcatt cattaatcta

9361 aaatttcttc aaagtgactt caatgatgat ctctaggcta tagtgaaagc tgaaagctta

9421 ttcgacaatg caagggaaag tgacgcacgt gtgtcgtatg ggaccgcgcg catttattct

9481 ctcagctaat tcccctaatc attactaatt gacggcacga tttctgcttc ttacttcctt

9541 ttactttgga gcttttcatc aataaaacca gtaccatggc cgtacgctca acggaaaagc

9601 attcaaaaaa acccgcgttc ctcgtgtgat ttgtgggtga gtggcgccat ctattagaga

9661 atagctgtac tacatctcgt ggacgaaggg gtcagagaag ttgaaagaga gcttgatcga

9721 ctgctatcca agctaggcga ggaagggaga tcgctagagc aaaagaaaaa aaataagcaa

9781 atatcttttt ttataacaaa tcgacgttag cgaaatatgt ttgaatcgat ttaacggtta

9841 gaattccctt tggttcgttc attatgcgat atctaattca attagagact aattcaatta

9901 gagctaattc aattaggatc caagcttatc gatttcgaac cctcgaccgc cggagtataa

9961 atagaggcgc ttcgtctacg gagcgacaat tcaattcaaa caagcaaagt gaacacgtcg

10021 ctaagcgaaa gctaagcaaa taaacaagcg cagctgaaca agctaaacaa tcggggtacc

10081 gctagagtcg acggtaccgc gggcccggga tccaccggtc gccaccatgg cctcctccga

10141 gaacgtcatc accgagttca tgcgcttcaa ggtgcgcatg gagggcaccg tgaacggcca

10201 cgagttcgag atcgagggcg agggcgaggg ccgcccctac gagggccaca acaccgtgaa

10261 gctgaaggtg accaagggcg gccccctgcc cttcgcctgg gacatcctgt ccccccagtt

10321 ccagtacggc tccaaggtgt acgtgaagca ccccgccgac atccccgact acaagaagct

10381 gtccttcccc gagggcttca agtgggagcg cgtgatgaac ttcgaggacg gcggcgtggc

10441 gaccgtgacc caggactcct ccctgcagga cggctgcttc atctacaagg tgaagttcat

10501 cggcgtgaac ttcccctccg acggccccgt gatgcagaag aagaccatgg gctgggaggc

10561 ctccaccgag cgcctgtacc cccgcgacgg cgtgctgaag ggcgagacgc acaaggccct

10621 gaagctgaag gacggcggcc actacctggt ggagttcaag tccatctaca tggccaagaa

10681 gcccgtgcag ctgcccggct actactacgt ggacgccaag ctggacatca cctcccacaa

10741 cgaggactac accatcgtgg agcagtacga gcgcaccgag ggccgccacc acctgttcct

10801 gagatctcga cccaagaaaa agcggaaggt ggaggacccg taagatccac cggatctaga

10861 taactgatca taatcagcca taccacattt gtagaggttt tacttgcttt aaaaaacctc

10921 ccacacctcc ccctgaacct gaaacataaa atgaatgcaa ttgttgttgt taacttgttt

10981 attgcagctt ataatggtta caaataaagc aatagcatca caaatttcac aaataaagca

11041 tttttttcac tgcattctag ttgtggtttg tccaaactca tcaatgtatc ttaacgccta

11101 gtgctgattc aaagtgtgtc tgcaggtatg ttcctataca ttacgcgtga aatatcgtca

11161 gggatggaag agatggcgaa ggttctcagc attggagcat ccacaagttg ttggtgatgc

11221 taacattgct tccgagcaat gtattgttgc cgagggtgtc gattagtaca gcaaagcgcg

11281 agaaacaagt ggctgctgtg gccggcggta cccataaacc agttgagata atttagacaa

11341 taaattgtaa taggcaattt gatgacgtcc cgcatgtacg gatcgtcaac gctgcacgct

11401 cgggaatagg gtgagaagtg ctgcacggtt actgaacagc gcaggtggtg attgtgttgt

11461 tttggtatca cgaaaacaag atagaaactg agaaaaaaga acctttttat cactttaccc

11521 gcaccgatac tggtctttcc tggatctttt cggggcgaag caaacaactc agcacgacat

11581 acaaattttg ggataatccc ttagtcagtg cctgtgtctt tctaatgaag attgacaatg

11641 gcggcggcgg cggcggtggt agcaaccaac gcactggcct ttgttttcat acttttcaat

11701 gaattcaatt agcttaacca acgaggttgt tgttgatgtt ctcacggaag tcaggagaag

11761 ctccggcggg cgtgtagggt aagtcgcagt tcggcagcat tctggcagtg tacatccagc

11821 acaccgagcg ttctagaacg ctgaatcgtt gaaacagagc aaatgactga agggctgtgt

11881 ctattgctgg gctgtacaga atgacgcatt tcgtgtggca atgtgttttc gaaagattga

11941 agcattctga tagcgccgtg tgtcaggaat gtaaatattt agatactgca gctaactaca

12001 aactgagaga gcttccagca agcaggtgta cctacgtggt gatcttttct agaaagaatg

12061 tagaattgac atgttgtgat gatttaccaa aaacaatttg agaagctaga gagtggattg

12121 ttctagccac cggaaccatc tggaatgttg aagttttctc gggaagaata gtagacaaaa

12181 agaaggaggg cttatcccct atagtgagtc gtattacatg gtcatagctg tttcctggca

12241 gctctggccc gtgtctcaaa atctctgatg ttacattgca caagataaaa atatatcatc

12301 atgaacaata aaactgtctg cttacataaa cagtaataca aggggtgtta tgagccatat

12361 tcaacgggaa acgtcgaggc cgcgattaaa ttccaacatg gatgctgatt tatatgggta

12421 taaatgggct cgcgataatg tcgggcaatc aggtgcgaca atctatcgct tgtatgggaa

12481 gcccgatgcg ccagagttgt ttctgaaaca tggcaaaggt agcgttgcca atgatgttac

12541 agatgagatg gtcagactaa actggctgac ggaatttatg cctcttccga ccatcaagca

12601 ttttatccgt actcctgatg atgcatggtt actcaccact gcgatccccg gaaaaacagc

12661 attccaggta ttagaagaat atcctgattc aggtgaaaat attgttgatg cgctggcagt

12721 gttcctgcgc cggttgcatt cgattcctgt ttgtaattgt ccttttaaca gcgatcgcgt

12781 atttcgtctc gctcaggcgc aatcacgaat gaataacggt ttggttgatg cgagtgattt

12841 tgatgacgag cgtaatggct ggcctgttga acaagtctgg aaagaaatgc ataaactttt

12901 gccattctca ccggattcag tcgtcactca tggtgatttc tcacttgata accttatttt

12961 tgacgagggg aaattaatag gttgtattga tgttggacga gtcggaatcg cagaccgata

13021 ccaggatctt gccatcctat ggaactgcct cggtgagttt tctccttcat tacagaaacg

13081 gctttttcaa aaatatggta ttgataatcc tgatatgaat aaattgcagt ttcatttgat

13141 gctcgatgag tttttctaat cagaattggt taattggttg taacactggc agagcattac

13201 gctgacttga cgggacggcg caagctcatg accaaaatcc cttaacgtga gttacgcgtc

13261 gttccactga gcgtcagacc ccgtagaaaa gatcaaagga tcttcttgag atcctttttt

13321 tctgcgcgta atctgctgct tgcaaacaaa aaaaccaccg ctaccagcgg tggtttgttt

13381 gccggatcaa gagctaccaa ctctttttcc gaaggtaact ggcttcagca gagcgcagat

13441 accaaatact gttcttctag tgtagccgta gttaggccac cacttcaaga actctgtagc

13501 accgcctaca tacctcgctc tgctaatcct gttaccagtg gctgctgcca gtggcgataa

13561 gtcgtgtctt accgggttgg actcaagacg atagttaccg gataaggcgc agcggtcggg

13621 ctgaacgggg ggttcgtgca cacagcccag cttggagcga acgacctaca ccgaactgag

13681 atacctacag cgtgagctat gagaaagcgc cacgcttccc gaagggagaa aggcggacag

13741 gtatccggta agcggcaggg tcggaacagg agagcgcacg agggagcttc cagggggaaa

13801 cgcctggtat ctttatagtc ctgtcgggtt tcgccacctc tgacttgagc gtcgattttt

13861 gtgatgctcg tcaggggggc ggagcctatg gaaaaacgcc agcaacgcgg cctttttacg

13921 gttcctggcc ttttgctggc cttttgctca catgtt

//

**Supplemental File 1D: *SagGD^zpg^***

LOCUS SagGDzpg 16588 bp DNA circular 14-DEC-2021

FEATURES Location/Qualifiers

misc_feature complement(603..727)

/note="attR4"

misc_feature 537..552

/note="M13F"

misc_feature complement(14849..14867)

/note="M13R"

misc_feature 843..2587

/note="5' homology arm saglin"

/note="saglin ORF"

misc_feature complement(2568..2587)

/note="5' flanking reverse primer"

misc_feature complement(2753..3073)

/note="U6 promoter"

misc_feature complement(2621..2732)

/note="gRNA template"

misc_feature 2621..3073

/note="p609"

misc_feature 2733..2752

/note="linker EM661/662"

misc_feature 2621..2649

/note="EM556"

misc_feature complement(3206..3526)

/note="U6 promoter"

misc_feature complement(3074..3185)

/note="gRNA template"

misc_feature 3074..3526

/note="p610"

misc_feature 3186..3205

/note="linker EM663/664"

misc_feature complement(3527..3634)

/note="gRNA template"

misc_feature 3527..3979

/note="p611"

misc_feature complement(3658..3979)

/note="U6 promoter"

misc_feature 3635..3658

/note="Linker EM665/666"

misc_feature 3980..3995

/note="EM723"

regulatory 6418..7491

/regulatory_class="promoter"

/note="Anopheles gambiae zero population growth"

gene 7498..11769

/gene="hCas9"

CDS 7498..11769

/gene="hCas9"

/codon_start=1

/transl_table=11

/product="hCas9"

/protein_id="AXV43861.1"

misc_feature complement(7458..7491)

/note="EM1384"

regulatory 11778..12815

/regulatory_class="terminator"

/note="Anopheles gambiae zero population growth"

misc_feature 12822..14762

/note="Saglin 3' homology arm"

misc_feature complement(4467..4574)

/label=gRNA template

/note="gRNA template"

misc_feature 4467..4483

/label=EM701

/note="EM701"

misc_feature complement(4441..4466)

/label=EM702

/note="EM702"

misc_feature complement(4392..4461)

/label=tRNA-gly

/note="tRNA-gly (missing terminal A, which is added when

cloning guide linker)"

misc_feature complement(4371..4395)

/label=EM1566

/note="EM1566"

misc_feature complement(4284..4391)

/label=gRNA template

/note="gRNA template"

misc_feature 4367..4391

/label=EM1567

/note="EM1567"

misc_feature 4284..4300

/label=EM703

/note="EM703"

misc_feature complement(4258..4283)

/label=EM704

/note="EM704"

misc_feature complement(4209..4278)

/label=tRNA-gly

/note="tRNA-gly (missing terminal A, which is added when

cloning guide linker)"

misc_feature complement(4188..4212)

/label=EM1568

/note="EM1568"

misc_feature complement(4101..4208)

/label=gRNA template

/note="gRNA template"

misc_feature 4184..4208

/label=EM1569

/note="EM1569"

misc_feature complement(4086..4110)

/label=EM850

/note="EM850"

misc_feature complement(4025..4095)

/label=tRNA-gly

/note="tRNA-gly"

misc_feature 4018..4041

/label=EM717

/note="EM717"

misc_feature 4018..4019

/note="EM1760"

misc_feature complement(4833..5157)

/label=U6 promoter (ag)

/note="U6 promoter (ag)"

primer_bind complement(4876..4894)

/label=EM1084

misc_feature complement(4807..4832)

/label=EM700

/note="EM700"

misc_feature complement(4758..4827)

/label=tRNA-gly

/note="tRNA-gly (missing terminal A, which is added when

cloning guide linker)"

misc_feature complement(4737..4761)

/label=EM1562

/note="EM1562"

misc_feature complement(4650..4757)

/label=gRNA template

/note="gRNA template"

misc_feature 4733..4757

/label=EM1563

/note="EM1563"

primer_bind 4740..4757

/label=EM1392

misc_feature 4650..4666

/label=EM705

/note="EM705"

misc_feature complement(4624..4649)

/label=EM924

/note="EM924"

misc_feature complement(4575..4644)

/label=tRNA-gly

/note="tRNA-gly (missing terminal A, which is added when

cloning guide linker)"

misc_feature complement(4554..4578)

/label=EM1564

/note="EM1564"

misc_feature 4550..4574

/label=EM1565

/note="EM1565"

misc_feature complement(5135..5157)

/label=EM895

/note="EM895"

primer_bind complement(5135..5157)

/label=EM895

misc_feature 5190..6413

/note="3xP3 DsRedNLS sv40 from 1154"

ORIGIN

1 CTTTCCTGCG TTATCCCCTG ATTCTGTGGA TAACCGTATT ACCGCCTTTG AGTGAGCTGA

61 TACCGCTCGC CGCAGCCGAA CGACCGAGCG CAGCGAGTCA GTGAGCGAGG AAGCGGAAGA

121 GCGCCCAATA CGCAAACCGC CTCTCCCCGC GCGTTGGCCG ATTCATTAAT GCAGCTGGCA

181 CGACAGGTTT CCCGACTGGA AAGCGGGCAG TGAGCGCAAC GCAATTAATA CGCGTACCGC

241 TAGCCAGGAA GAGTTTGTAG AAACGCAAAA AGGCCATCCG TCAGGATGGC CTTCTGCTTA

301 GTTTGATGCC TGGCAGTTTA TGGCGGGCGT CCTGCCCGCC ACCCTCCGGG CCGTTGCTTC

361 ACAACGTTCA AATCCGCTCC CGGCGGATTT GTCCTACTCA GGAGAGCGTT CACCGACAAA

421 CAACAGATAA AACGAAAGGC CCAGTCTTCC GACTGAGCCT TTCGTTTTAT TTGATGCCTG

481 GCAGTTCCCT ACTCTCGCGT TAACGCTAGC ATGGATGTTT TCCCAGTCAC GACGTTGTAA

541 AACGACGGCC AGTCTTAAGC TCGGGCCCCT ACAGGTCACT AATACCATCT AAGTAGTTGA

601 TTCATAGTGA CTGGATATGT TGTGTTTTAC AGTATTATGT AGTCTGTTTT TTATGCAAAA

661 TCTAATTTAA TATATTGATA TTTATATCAT TTTACGTTTC TCGTTCAACT TTTCTATACA

721 AAGTTGGTAC CGGGCCCCCC GCTAGCGTCG ACGGTATCGA TAAGCTTGAT cggatccTGC

781 GGGTGCCAGG GCGTGCCCTT GGGCTCCCCG GGCGCGTACT CCACCTCACC CATaggcctg

841 ataacaCACT GGCACGACGA TCATCCCTTT CTGCTCGAAA CGGTCGCGAA GGCGTACGAG

901 CCGCTCAAGG CCGCGCTGGA GTCGGAGCAG GATGCGCATC GAAGCCTTCG GCTGGTGCCC

961 GAGCTGCAGC GCAAGCAGCT CATCTACAGC ATCGAGGCGG GCCGGCTCGA GGACGCCCAC

1021 ATCCTGCACA TGACGCTCAA GGGCCAGTGG AAGCCGGACC AAATCGTGTC GGCCATCCAG

1081 GACGGCTACC ACGTCAACCC GACGATCATG GAGCATCTGC TCGAGTTCGT CCGCGCCATC

1141 CCGGTGCGGA AGGAGCGGGC CGCCTACTAC AAAGCGATCG GGCCGGTCAT TCGCAACTTC

1201 AAGCTGACCC TGACGTACGT GACGCTCCTG TTCGCGGGCG ATGCGACCGG CGTGTTTGAC

1261 ACGCGAAAGG ATCGCGACGA CTACATCTCG CAACCGCTCA CCGTGTTCGT GCACATGCTG

1321 CGCTGGCAGC TGGCGAACCT CGAGTTCGAG TTCCACCTGT CGCTGGCGGA GCGCTTCCCC

1381 CGCTACTACT CGCTGCACAT CGAGCAAATC TTCTTCTTTG CGCCCCCGCA CTGGCAGAAG

1441 GCGGAGAAGC GCCGGCTGTT CGAGATCGCG AGCCTGTTCA AGGCGAAGGG GCACCGGTTT

1501 GCGGCGATCG AGCAGCTGCT GAAGTGGGCG CACCAGTACG CACCGATGCG CGGTGGCAAA

1561 GCCCACCTGG AGGAAATACT GCCCACGCTG GCGCTCGAAA CGGAGAAGCT GCGGCGCACG

1621 GTCGAGCAGA CGGGCAAGAG CTCAGCGGAG CTGGTGCGGC TCAAGAAGCT GGAGGAAAAG

1681 TTCGTAAGCA AAAAGGATCG CTGGAACACG AACACCTACC GCACGTACCT GCACATGATC

1741 AAGACGAAGG GTAAGTTCGG CCAGCATAGG ATGGAAATGG AGCGCAAGGG TTAAATAGCG

1801 GGAATGGCAC ATGCGAGGCG ATGGATGAAA TACAAGTCGT TTGGTGAGCG GAGTCACCAA

1861 GCAAGCATAA CTTTCATGCG TACAATGTGT ATGATCACTG ATGACAAAGC GCTTGGGTGG

1921 GGCGCTTGCT GCTGAAGCGT GGCTGACAAA TGGGGGGGGG GCTTGAACAA ATATTTGCAC

1981 ACACGATAAT GCTCTTGTTT GCCATGCGCT GACGGAAGAC AAATCGCTGC AAGCGACGGC

2041 TCAAACCTGC TCAAACCAGG GAAGGTTTGC ATAAGGTTCA CTTATAAATG AGCGTTCGTG

2101 GCTACTCGGG CGTTCAAGTC ATCTCGAGCA GGAAGCACCG CAGCATGTCA CGTTTGCCCA

2161 CTGTACTGTT GCTCTTAGCG AGCGCCGCAG TCCTCGCGGC TGGCGGCCAG GAAGCAACCG

2221 AAGACCCGTT CGCGGACGAA ACCGACCAGT GCCAGATCAG CGTTAGTGCC GAGACGATGA

2281 AATCCCTCCA CGGCGGGTCG ATGCAGCCCG ACGGCACGTG CGACAACCTG TGGGAAAGCT

2341 TTCTATCTCA GTTTCACCAA GTCAGGGAAA ACCTGACCGC GTGCCAGGAG CGGGCAGCCG

2401 CCGGACCAGC GCCCGATCCT TCCAGCCAGT TCTGTCAGCA GCTGCTGGAC GATGCGCAGC

2461 GGCAGATGGA GCAGGAGCAT CGCCAGTACG CCGCCACCCT GGAGGAGCAG CTGCACGCAG

2521 CGCAGCAGGA AACCCAGCAG GAGCAGGAGA TGAAGAAGGC GCTGCAGAAG CAGCTCGACG

2581 CGCTCACGTA AGCGTCGACG GTATCGATAA GCTTGATCgg atccGATGAA AATAAGAAAA

2641 ACATTTGACA AAAAAAGCAC CGACTCGGTG CCACTTTTTC AAGTTGATAA CGGACTAGCC

2701 TTATTTTAAC TTGCTATTTC TAGCTCTAAA ACCCTGCTTG AAGTTACCGT TCAAGGACGA

2761 GGGGAAAAAA GGTTGTATAT ATACTTTGCG CTTTCAATCC TTGCTCTAGC GATGCATAAA

2821 GGATATTCAA GAAGGATTTT TTGCCTGAGT GTGACTGTTA TAAGGTTTTG ATCCTGCCGT

2881 AACTCACGCT AATGTGATGT TTCATAAACT TTTCAACATT TCTTGTATTG CTTCATCTCT

2941 TTTTTAATCA AAAGTTACTA TTCATTGTAA TACGTTGAAA TATCAAAAAA GAATAAACAG

3001 TTTTCCTATG ATTAATTCAA AAACAGAGTT TTCTAGAATA ACCAAGAGCC AAACCGCTTC

3061 AGTGTATATG TGGggaaGAT GAAAATAAGA AAAACATTTG ACAAAAAAAG CACCGACTCG

3121 GTGCCACTTT TTCAAGTTGA TAACGGACTA GCCTTATTTT AACTTGCTAT TTCTAGCTCT

3181 AAAACCAACG CGCTGTACAT CGATCAAGGA CGAGGGGAAA AAAGGTTGTA TATATACTTT

3241 GCGCTTTCAA TCCTTGCTCT AGCGATGCAT AAAGGATATT CAAGAAGGAT TTTTTGCCTG

3301 AGTGTGACTG TTATAAGGTT TTGATCCTGC CGTAACTCAC GCTAATGTGA TGTTTCATAA

3361 ACTTTTCAAC ATTTCTTGTA TTGCTTCATC TCTTTTTTAA TCAAAAGTTA CTATTCATTG

3421 TAATACGTTG AAATATCAAA AAAGAATAAA CAGTTTTCCT ATGATTAATT CAAAAACAGA

3481 GTTTTCTAGA ATAACCAAGA GCCAAACCGC TTCAGTGTAT ATGTGGagag GATGAAAATA

3541 AGAAAAACAT TTGACAAAAA AAGCACCGAC TCGGTGCCAC TTTTTCAAGT TGATAACGGA

3601 CTAGCCTTAT TTTAACTTGC TATTTCTAGC TCTAAAACCT CGTACTACAA CCTGATGCAA

3661 GGACGAGGGG AAAAAAGGTT GTATATATAC TTTGCGCTTT CAATCCTTGC TCTAGCGATG

3721 CATAAAGGAT ATTCAAGAAG GATTTTTTGC CTGAGTGTGA CTGTTATAAG GTTTTGATCC

3781 TGCCGTAACT CACGCTAATG TGATGTTTCA TAAACTTTTC AACATTTCTT GTATTGCTTC

3841 ATCTCTTTTT TAATCAAAAG TTACTATTCA TTGTAATACG TTGAAATATC AAAAAAGAAT

3901 AAACAGTTTT CCTATGATTA ATTCAAAAAC AGAGTTTTCT AGAATAACCA AGAGCCAAAC

3961 CGCTTCAGTG TATATGTGGa acaCACTGGC ACGACATCGT CCCCACCGCC AGCGAATaaa

4021 aaaatgcatc ggccgggaat cgaacccggg ccgcccgcgt ggcaggcgag cattctacca

4081 ctgaaccacc gatgcttcgt agcaccgact cggtgccact ttttcaagtt gataacggac

4141 tagccttatt tcaacttgct atgctgtttc cagcatagct ctgaaactcc ctgacgatat

4201 ttcacgctgc atcggccggg aatcgaaccc gggccgcccg cgtggcaggc gagcattcta

4261 ccactgaacc accgatgctt tccagcaccg actcggtgcc actttttcaa gttgataacg

4321 gactagcctt atttcaactt gctatgctgt ttccagcata gctctgaaac gcggacacac

4381 tttgaatcag tgcatcggcc gggaatcgaa cccgggccgc ccgcgtggca ggcgagcatt

4441 ctaccactga accaccgatg ctatggagca ccgactcggt gccacttttt caagttgata

4501 acggactagc cttatttcaa cttgctatgc tgtttccagc atagctctga aacggaagct

4561 ccagagcagc ctctgcatcg gccgggaatc gaacccgggc cgcccgcgtg gcaggcgagc

4621 attctaccac tgaaccaccg atgctctcta gcaccgactc ggtgccactt tttcaagttg

4681 ataacggact agccttattt caacttgcta tgctgtttcc agcatagctc tgaaacagga

4741 cccacatcgt tccgtgtgca tcggccggga atcgaacccg ggccgcccgc gtggcaggcg

4801 agcattctac cactgaacca ccgatgcttt acaaggacga ggggaaaaaa ggttgtatat

4861 atactttgcg ctttcaatcc ttgctctagc gatgcataaa ggatattcaa gaaggatttt

4921 ttgcctgagt gtgactgtta taaggttttg atcctgccgt aactcacgct aatgtgatgt

4981 ttcataaact tttcaacatt tcttgtattg cttcatctct tttttaatca aaagttacta

5041 ttcattgtaa tacgttgaaa tatcaaaaaa gaataaacag ttttcctatg attaattcaa

5101 aaacagagtt ttctagaata accaagagcc aaaccgcttc agtgtatatg tggataaACC

5161 CACATCGTTC CGTATCTGAG ACCGATATCT AATTCAATTA GAGACTAATT CAATTAGAGC

5221 TAATTCAATT AGGATCCAAG CTTATCGATT TCGAACCCTC GACCGCCGGA GTATAAATAG

5281 AGGCGCTTCG TCTACGGAGC GACAATTCAA TTCAAACAAG CAAAGTGAAC ACGTCGCTAA

5341 GCGAAAGCTA AGCAAATAAA CAAGCGCAGC TGAACAAGCT AAACAATCGG GGTACCGCTA

5401 GAGTCGACGG TACCGCGGGC CCGGGATCCA ccggtcgcca ccatggcctc ctccgagaac

5461 gtcatcaccg agttcatgcg cttcaaggtg cgcatggagg gcaccgtgaa cggccacgag

5521 ttcgagatcg agggcgaggg cgagggccgc ccctacgagg gccacaacac cgtgaagctg

5581 aaggtgacca agggcggccc cctgcccttc gcctgggaca tcctgtcccc ccagttccag

5641 tacggctcca aggtgtacgt gaagcacccc gccgacatcc ccgactacaa gaagctgtcc

5701 ttccccgagg gcttcaagtg ggagcgcgtg atgaacttcg aggacggcgg cgtggcgacc

5761 gtgacccagg actcctccct gcaggacggc tgcttcatct acaaggtgaa gttcatcggc

5821 gtgaacttcc cctccgacgg ccccgtgatg cagaagaaga ccatgggctg ggaggcctcc

5881 accgagcgcc tgtacccccg cgacggcgtg ctgaagggcg agacgcacaa ggccctgaag

5941 ctgaaggacg gcggccacta cctggtggag ttcaagtcca tctacatggc caagaagccc

6001 gtgcagctgc ccggctacta ctacgtggac gccaagctgg acatcacctc ccacaacgag

6061 gactacacca tcgtggagca gtacgagcgc accgagggcc gccaccacct gttcctgaga

6121 tctcgaccca agaaaaagcg gaaggtggag gacccgtaag atccaccgga tctagataac

6181 tgatcataat cagccatacc acatttgtag aggttttact tgctttaaaa aacctcccac

6241 acctccccct gaacctgaaa cataaaatga atgcaattgt tgttgttaac ttgtttattg

6301 cagcttataa tggttacaaa taaagcaata gcatcacaaa tttcacaaat aaagcatttt

6361 tttcactgca ttctagttgt ggtttgtcca aactcatcaa tgtatcttaT TATcggtcag

6421 cgctggcggt ggggacagct ccggctgtgg ctgttcttgc gagtcctctt cctgcggcac

6481 atccctctcg tcgaccagtt cagtttgctg agcgtaagcc tgctgctgtt cgtcctgcat

6541 catcgggacc atttgtatgg gccatccgcc accaccacca tcaccaccgc cgtccatttc

6601 taggggcata cccatcagca tctccgcggg cgccattggc ggtggtgcca aggtgccatt

6661 cgtttgttgc tgaaagcaaa agaaagcaaa ttagtgttgt ttctgctgca cacgataatt

6721 ttcgtttctt gccgctagac acaaacaaca ctgcatctgg agggagaaat ttgacgccta

6781 gctgtataac ttacctcaaa gttattgtcc atcgtggtat aatggaccta ccgagcccgg

6841 ttacactaca caaagcaaga ttatgcgaca aaatcacagc gaaaactagt aattttcatc

6901 tatcgaaagc ggccgagcag agagttgttt ggtattgcaa cttgacattc tgctgcggga

6961 taaaccgcga cgggctacca tggcgcacct gtcagatggc tgtcaaattt ggcccggttt

7021 gcgatatgga gtgggtgaaa ttatatccca ctcgctgatc gtgaaaatag acacctgaaa

7081 acaataattg ttgtgttaat tttacatttt gaagaacagc acaagttttg ctgacaatat

7141 ttaattacgt ttcgttatca acggcacgga aagattatct cgctgattat ccctctcgct

7201 ctctctgtct atcatgtcct ggtcgttctc gcgtcacccc ggataatcga gagacgccat

7261 ttttaatttg aactactaca ccgacaagca tgccgtgagc tctttcaagt tcttctgtcc

7321 gaccaaagaa acagagaata ccgcccggac agtgcccgga gtgatcgatc catagaaaat

7381 cgcccatcat gtgccactga ggcgaaccgg cgtagcttgt tccgaatttc caagtgcttc

7441 cccgtaacat ccgcatataa caaacagccc aacaacaaat acagcatcga gctcgagatg

7501 gactataagg accacgacgg agactacaag gatcatgata ttgattacaa agacgatgac

7561 gataagatgg ccccaaagaa gaagcggaag gtcggtatcc acggagtccc agcagccgac

7621 aagaagtaca gcatcggcct ggacatcggc accaactctg tgggctgggc cgtgatcacc

7681 gacgagtaca aggtgcccag caagaaattc aaggtgctgg gcaacaccga ccggcacagc

7741 atcaagaaga acctgatcgg agccctgctg ttcgacagcg gcgaaacagc cgaggccacc

7801 cggctgaaga gaaccgccag aagaagatac accagacgga agaaccggat ctgctatctg

7861 caagagatct tcagcaacga gatggccaag gtggacgaca gcttcttcca cagactggaa

7921 gagtccttcc tggtggaaga ggataagaag cacgagcggc accccatctt cggcaacatc

7981 gtggacgagg tggcctacca cgagaagtac cccaccatct accacctgag aaagaaactg

8041 gtggacagca ccgacaaggc cgacctgcgg ctgatctatc tggccctggc ccacatgatc

8101 aagttccggg gccacttcct gatcgagggc gacctgaacc ccgacaacag cgacgtggac

8161 aagctgttca tccagctggt gcagacctac aaccagctgt tcgaggaaaa ccccatcaac

8221 gccagcggcg tggacgccaa ggccatcctg tctgccagac tgagcaagag cagacggctg

8281 gaaaatctga tcgcccagct gcccggcgag aagaagaatg gcctgttcgg aaacctgatt

8341 gccctgagcc tgggcctgac ccccaacttc aagagcaact tcgacctggc cgaggatgcc

8401 aaactgcagc tgagcaagga cacctacgac gacgacctgg acaacctgct ggcccagatc

8461 ggcgaccagt acgccgacct gtttctggcc gccaagaacc tgtccgacgc catcctgctg

8521 agcgacatcc tgagagtgaa caccgagatc accaaggccc ccctgagcgc ctctatgatc

8581 aagagatacg acgagcacca ccaggacctg accctgctga aagctctcgt gcggcagcag

8641 ctgcctgaga agtacaaaga gattttcttc gaccagagca agaacggcta cgccggctac

8701 attgacggcg gagccagcca ggaagagttc tacaagttca tcaagcccat cctggaaaag

8761 atggacggca ccgaggaact gctcgtgaag ctgaacagag aggacctgct gcggaagcag

8821 cggaccttcg acaacggcag catcccccac cagatccacc tgggagagct gcacgccatt

8881 ctgcggcggc aggaagattt ttacccattc ctgaaggaca accgggaaaa gatcgagaag

8941 atcctgacct tccgcatccc ctactacgtg ggccctctgg ccaggggaaa cagcagattc

9001 gcctggatga ccagaaagag cgaggaaacc atcaccccct ggaacttcga ggaagtggtg

9061 gacaagggcg cttccgccca gagcttcatc gagcggatga ccaacttcga taagaacctg

9121 cccaacgaga aggtgctgcc caagcacagc ctgctgtacg agtacttcac cgtgtataac

9181 gagctgacca aagtgaaata cgtgaccgag ggaatgagaa agcccgcctt cctgagcggc

9241 gagcagaaaa aggccatcgt ggacctgctg ttcaagacca accggaaagt gaccgtgaag

9301 cagctgaaag aggactactt caagaaaatc gagtgcttcg actccgtgga aatctccggc

9361 gtggaagatc ggttcaacgc ctccctgggc acataccacg atctgctgaa aattatcaag

9421 gacaaggact tcctggacaa tgaggaaaac gaggacattc tggaagatat cgtgctgacc

9481 ctgacactgt ttgaggacag agagatgatc gaggaacggc tgaaaaccta tgcccacctg

9541 ttcgacgaca aagtgatgaa gcagctgaag cggcggagat acaccggctg gggcaggctg

9601 agccggaagc tgatcaacgg catccgggac aagcagtccg gcaagacaat cctggatttc

9661 ctgaagtccg acggcttcgc caacagaaac ttcatgcagc tgatccacga cgacagcctg

9721 acctttaaag aggacatcca gaaagcccag gtgtccggcc agggcgatag cctgcacgag

9781 cacattgcca atctggccgg cagccccgcc attaagaagg gcatcctgca gacagtgaag

9841 gtggtggacg agctcgtgaa agtgatgggc cggcacaagc ccgagaacat cgtgatcgaa

9901 atggccagag agaaccagac cacccagaag ggacagaaga acagccgcga gagaatgaag

9961 cggatcgaag agggcatcaa agagctgggc agccagatcc tgaaagaaca ccccgtggaa

10021 aacacccagc tgcagaacga gaagctgtac ctgtactacc tgcagaatgg gcgggatatg

10081 tacgtggacc aggaactgga catcaaccgg ctgtccgact acgatgtgga ccatatcgtg

10141 cctcagagct ttctgaagga cgactccatc gacaacaagg tgctgaccag aagcgacaag

10201 aaccggggca agagcgacaa cgtgccctcc gaagaggtcg tgaagaagat gaagaactac

10261 tggcggcagc tgctgaacgc caagctgatt acccagagaa agttcgacaa tctgaccaag

10321 gccgagagag gcggcctgag cgaactggat aaggccggct tcatcaagag acagctggtg

10381 gaaacccggc agatcacaaa gcacgtggca cagatcctgg actcccggat gaacactaag

10441 tacgacgaga atgacaagct gatccgggaa gtgaaagtga tcaccctgaa gtccaagctg

10501 gtgtccgatt tccggaagga tttccagttt tacaaagtgc gcgagatcaa caactaccac

10561 cacgcccacg acgcctacct gaacgccgtc gtgggaaccg ccctgatcaa aaagtaccct

10621 aagctggaaa gcgagttcgt gtacggcgac tacaaggtgt acgacgtgcg gaagatgatc

10681 gccaagagcg agcaggaaat cggcaaggct accgccaagt acttcttcta cagcaacatc

10741 atgaactttt tcaagaccga gattaccctg gccaacggcg agatccggaa gcggcctctg

10801 atcgagacaa acggcgaaac cggggagatc gtgtgggata agggccggga ttttgccacc

10861 gtgcggaaag tgctgagcat gccccaagtg aatatcgtga aaaagaccga ggtgcagaca

10921 ggcggcttca gcaaagagtc tatcctgccc aagaggaaca gcgataagct gatcgccaga

10981 aagaaggact gggaccctaa gaagtacggc ggcttcgaca gccccaccgt ggcctattct

11041 gtgctggtgg tggccaaagt ggaaaagggc aagtccaaga aactgaagag tgtgaaagag

11101 ctgctgggga tcaccatcat ggaaagaagc agcttcgaga agaatcccat cgactttctg

11161 gaagccaagg gctacaaaga agtgaaaaag gacctgatca tcaagctgcc taagtactcc

11221 ctgttcgagc tggaaaacgg ccggaagaga atgctggcct ctgccggcga actgcagaag

11281 ggaaacgaac tggccctgcc ctccaaatat gtgaacttcc tgtacctggc cagccactat

11341 gagaagctga agggctcccc cgaggataat gagcagaaac agctgtttgt ggaacagcac

11401 aagcactacc tggacgagat catcgagcag atcagcgagt tctccaagag agtgatcctg

11461 gccgacgcta atctggacaa agtgctgtcc gcctacaaca agcaccggga taagcccatc

11521 agagagcagg ccgagaatat catccacctg tttaccctga ccaatctggg agcccctgcc

11581 gccttcaagt actttgacac caccatcgac cggaagaggt acaccagcac caaagaggtg

11641 ctggacgcca ccctgatcca ccagagcatc accggcctgt acgagacacg gatcgacctg

11701 tctcagctgg gaggcgacaa aaggccggcg gccacgaaaa aggccggcca ggcaaaaaag

11761 aaaaagtaat taattaagag gacggcgaga agtaatcata tgtccgcatt ttgcgcaaac

11821 caggcgctta gacaatttgc gcgtaagcac attcgaaatg tgcaaagctg aaagcagtgg

11881 tttcgccagc ccgagttcag cgaaacggat tccttccaag tgtttgcatt cctggcggag

11941 tattcctccc aaaatgcact caccctgcgt gcagtgccaa atcgtgagtt tcctaatttt

12001 ttcatattgt ttattaccta ccaactaaag ttgttgttat atattgcgtt ttacgtacga

12061 caaataagtt cgtattcaga aatatttgcg ataagagaga actcatttgc gatgaatctc

12121 attgtattta gctaagtgcc ttgataagta agcggaacag caggaatatg acactccttg

12181 ggaaatacat gtaagcgtct gtgaattaga tatatataca cgcaaccaaa tggtccatgg

12241 ttgatttaag cactgcctgt tgtcgaacat tgctataagc aaaataaaga agcattcatt

12301 aatctaaaat ttcttcaaag tgacttcaat gatgatctct aggctatagt gaaagctgaa

12361 agcttattcg acaatgcaag ggaaagtgac gcacgtgtgt cgtatgggac cgcgcgcatt

12421 tattctctca gctaattccc ctaatcatta gtaattgacg gcacgatttc tgcttcttac

12481 ttccttttac tttggagctt ttcatcaata aaaccagtac catggccgta cgctcaacgg

12541 aaaagcattc acaaaaaccc gcgttcctcg tgtgatttgt gggtgagtgg cgccatctat

12601 tagagaatag ctgtactaca tctcgtggac gaaggggtca gagaagttga aagagagctt

12661 gatcgactgc tatccaagct aggcgaggaa gggagatcgc tagagcaaaa gaaaaaaaat

12721 aagcaaatat ctttttttat aacaaatcga cgttagcgaa atatgtttga atcgatttaa

12781 cggttagaat tccctttggt tcgttcatta tgcgaggcgc gacgcAGCTG CAAACCAGCC

12841 TCGCCACGAT CGAGCTGAAG CACTGGCGCA AGTTCGACCG GTTCGTACCG TACGCTAACG

12901 CGCTGCCCCA GCCAGCCCAG CGGCTGGAGG TGCTGCGTGT GCTGCTGAGC CAGATCGGCG

12961 ACCGGGAGAA GAAGACTTCC CACAAATATC TGGTAAAGGC GGCACGGCAG TTTGACATTT

13021 GCGAGCAGTT TATCGGCCGG GGCAAGGTGG ACCAGGCGGT CAAGAAGCAG CTGGAGGAAC

13081 TGCGCGGGAA GTTTGCAACG TTTGCGAAGG GCAAGAACTA CCAGCACTAT CTGAGCGAGT

13141 CGCGCAAGTC GTCCGGCTAG GCGTCGGCGA CGGTCTAAAA GTGATTCAAA GCCCCGTAGG

13201 CGAGGAACCC CCGTACGTGT CGTGTAGCGC AAACCCTTCA CCATCATAGC ACAGTGAAAT

13261 AAAGCAAGAA TAAAAATTAA AAAAAAAAAC AATTGTAATC GAAATATTGA GCAACAGCAA

13321 AGCAAAGCCT TTCCTTTGGA TCATCCGCAA TCGGCACGTT GCGAGTTGGG CGGCAGCGTT

13381 GCTTGTGCAA TCGCTGGACC CATGCTGGAC AAACAGATGA GCACACTGGC CTTCCCAGCC

13441 AGCTAATATT GACACTTCAT TCGAGCTGGA CCATTAATCA CGGCGTCTGT TTGCCTTTGG

13501 GCCAAGTATA ACTAGCACCA CGCACCGAAC AGACGGGCTC TAGTTTTGTT TCTCTTGCTG

13561 TGAGCGTCAG TTAATTAAAA AAACAAACCT ATGGATCACA GCAGAGTGTG GTGCTGTCTG

13621 CTTAGCTTCA TCGCAATGGC CGTGCTGTGT AGCGCATCCA GTGCAGCATG CACGCTCCAG

13681 GTCCCCGAAA CCATGATAAC GCGCCTGCTG CAAGCGGATC AGCCCGCCGG CGGCTGCGCA

13741 GCTGCTTGGG ATAGTCTACT GTCTCGGCTG GAGCAAATGC GCCACAATCT TACGGGCTGC

13801 AGCGAGCGCG AAACGACGAA CCCGGCACCA CCCGATGATA CCCGCACCAA CGCCCCCTGT

13861 CAGCTGCTGC TGAACGAGCT GCAACGGAAA GGGAATCAAA CCCTGCTCCA GCTCGACAAA

13921 GAAATGGCCA GCAAAGCACT GTACGAGCGC ATCGAGCTGG CCAAGGTGGT GCAGGACCAG

13981 GACCGCATCC AACAAGCGAT CGAAACGATG CGCACCGAGC AGCGAAACCA CCGGCAGCAG

14041 CTGCTACTCC TCTACCTCGA TGCGGCGGAC CTACGACGAG CCCTGCACCA GTATAAGCTG

14101 CTCGTCGCGC AGGGCGATCG CCACCTTCCC CAGCAGATAG TGAAGTTCGT GTACGCCGCC

14161 CCACGGCACG AGAACCGCCG GCTCGAGAAC CTGCTCGACC TGGTGCGGCA GCTGCCGGCG

14221 CGCCAGGACC AGCGCACCCT GTACCAGCTG CTGCAGCCGG AAATTATGAA GCGCCCCACG

14281 CAGAACCAGA GCACCCTCGC CATGCTGACA GCGCTCGAGA TGGGCCAGGT CGTGGAGGGG

14341 AACGGCGAGC TGAAAAAGCA GCAGGACGCG ATGTACCAGC TGGTGCTGAA GCGCTGGATG

14401 TTTCTGTGCC TCGCCGGCCA GTATCGCGAG ATAGTGCAGT TTGCCACGAA GCACCCGCGC

14461 CTGTTCGAGC AGATCGGGGC GAAGATCGCC ACGATTCATC CGAAGTACTG GTGGAAGTTT

14521 AGCTTCACCC AGTTCGTGAC GTACCCGAAC CTGTTGCCGC TGCCCGAGCA GCGGCTGGAA

14581 GCGTTCCGCA CCATCATGAA GCAGCTGAAG CAGCGCAATG GGAAGTTTTT CGCCGACCAT

14641 CTGGCGAAGC TGGCACCCCA GATCGAGCGC TGCGAGCAGT TTCTGCGGCA GCAGAAGAAG

14701 GAGCTGGAGT GGAAGGACGA GCTGGTGAAG CTGAAGGGAC AGTTTGCCGA CTTTGACCgc

14761 ttatcATGGG TGAGGTGGAG TACGCGCCCG GGGAGCCCAA GGGCACGCCC TGGCACCCGC

14821 AgcttATCCC CTATAGTGAG TCGTATTACA TGGTCATAGC TGTTTCCTGG CAGCTCTGGC

14881 CCGTGTCTCA AAATCTCTGA TGTTACATTG CACAAGATAA AAATATATCA TCATGAACAA

14941 TAAAACTGTC TGCTTACATA AACAGTAATA CAAGGGGTGT TATGAGCCAT ATTCAACGGG

15001 AAACGTCGAG GCCGCGATTA AATTCCAACA TGGATGCTGA TTTATATGGG TATAAATGGG

15061 CTCGCGATAA TGTCGGGCAA TCAGGTGCGA CAATCTATCG CTTGTATGGG AAGCCCGATG

15121 CGCCAGAGTT GTTTCTGAAA CATGGCAAAG GTAGCGTTGC CAATGATGTT ACAGATGAGA

15181 TGGTCAGACT AAACTGGCTG ACGGAATTTA TGCCTCTTCC GACCATCAAG CATTTTATCC

15241 GTACTCCTGA TGATGCATGG TTACTCACCA CTGCGATCCC CGGAAAAACA GCATTCCAGG

15301 TATTAGAAGA ATATCCTGAT TCAGGTGAAA ATATTGTTGA TGCGCTGGCA GTGTTCCTGC

15361 GCCGGTTGCA TTCGATTCCT GTTTGTAATT GTCCTTTTAA CAGCGATCGC GTATTTCGTC

15421 TCGCTCAGGC GCAATCACGA ATGAATAACG GTTTGGTTGA TGCGAGTGAT TTTGATGACG

15481 AGCGTAATGG CTGGCCTGTT GAACAAGTCT GGAAAGAAAT GCATAAACTT TTGCCATTCT

15541 CACCGGATTC AGTCGTCACT CATGGTGATT TCTCACTTGA TAACCTTATT TTTGACGAGG

15601 GGAAATTAAT AGGTTGTATT GATGTTGGAC GAGTCGGAAT CGCAGACCGA TACCAGGATC

15661 TTGCCATCCT ATGGAACTGC CTCGGTGAGT TTTCTCCTTC ATTACAGAAA CGGCTTTTTC

15721 AAAAATATGG TATTGATAAT CCTGATATGA ATAAATTGCA GTTTCATTTG ATGCTCGATG

15781 AGTTTTTCTA ATCAGAATTG GTTAATTGGT TGTAACACTG GCAGAGCATT ACGCTGACTT

15841 GACGGGACGG CGCAAGCTCA TGACCAAAAT CCCTTAACGT GAGTTACGCG TCGTTCCACT

15901 GAGCGTCAGA CCCCGTAGAA AAGATCAAAG GATCTTCTTG AGATCCTTTT TTTCTGCGCG

15961 TAATCTGCTG CTTGCAAACA AAAAAACCAC CGCTACCAGC GGTGGTTTGT TTGCCGGATC

16021 AAGAGCTACC AACTCTTTTT CCGAAGGTAA CTGGCTTCAG CAGAGCGCAG ATACCAAATA

16081 CTGTTCTTCT AGTGTAGCCG TAGTTAGGCC ACCACTTCAA GAACTCTGTA GCACCGCCTA

16141 CATACCTCGC TCTGCTAATC CTGTTACCAG TGGCTGCTGC CAGTGGCGAT AAGTCGTGTC

16201 TTACCGGGTT GGACTCAAGA CGATAGTTAC CGGATAAGGC GCAGCGGTCG GGCTGAACGG

16261 GGGGTTCGTG CACACAGCCC AGCTTGGAGC GAACGACCTA CACCGAACTG AGATACCTAC

16321 AGCGTGAGCT ATGAGAAAGC GCCACGCTTC CCGAAGGGAG AAAGGCGGAC AGGTATCCGG

16381 TAAGCGGCAG GGTCGGAACA GGAGAGCGCA CGAGGGAGCT TCCAGGGGGA AACGCCTGGT

16441 ATCTTTATAG TCCTGTCGGG TTTCGCCACC TCTGACTTGA GCGTCGATTT TTGTGATGCT

16501 CGTCAGGGGG GCGGAGCCTA TGGAAAAACG CCAGCAACGC GGCCTTTTTA CGGTTCCTGG

16561 CCTTTTGCTG GCCTTTTGCT CACATGTT

//

**Supplemental File 1E: *SagGD^vasa^***

LOCUS SagGDvasa 17175 bp ds-DNA circular

DEFINITION synthetic circular DNA

ACCESSION .

VERSION .

KEYWORDS .

SOURCE synthetic DNA construct

ORGANISM synthetic DNA construct

REFERENCE 1 (bases 1 to 17175)

AUTHORS .

TITLE Direct Submission

JOURNAL Exported Wednesday, Dec 14, 2022 from SnapGene 5.2.5

https://www.snapgene.com

FEATURES Location/Qualifiers

misc_feature 537..552

/label=M13F

/note="M13F"

misc_feature 778..833

/label=attB 1

/note="attB 1"

misc_feature 781..818

/label=minimal attb

/note="minimal attb"

misc_feature 806..833

/label=EM1025

/note="EM1025"

misc_feature 843..2587

/label=5' homology arm saglin

/note="5' homology arm saglin"

misc_feature 2088..2587

/label=saglin ORF

/note="saglin ORF"

misc_feature 2621..3073

/label=p609

/note="p609"

misc_feature complement(2621..2732)

/label=gRNA template

/note="gRNA template"

misc_feature 2733..2752

/label="linker EM661/662"

/note="Saglin gRNA 3"

misc_feature complement(2753..3073)

/label=U6 promoter

/note="U6 promoter"

misc_feature 3074..3526

/label=p610

/note="p610"

misc_feature complement(3074..3185)

/label=gRNA template

/note="gRNA template"

misc_feature 3186..3205

/label="linker EM663/664"

/note="Saglin gRNA2"

misc_feature complement(3206..3526)

/label=U6 promoter

/note="U6 promoter"

misc_feature 3527..3979

/label=p611

/note="p611"

misc_feature complement(3527..3634)

/label=gRNA template

/note="gRNA template"

misc_feature 3635..3658

/label="Linker EM665/666"

/note="Saglin gRNA1"

misc_feature complement(3658..3979)

/label=U6 promoter

/note="U6 promoter"

misc_feature 3980..3995

/label=EM723

/note="EM723"

misc_feature 4014..4049

/label=Ag U6 terminator

/note="from Ag U6"

misc_feature 4014..4042

/label=EM556

/note="EM556"

misc_feature complement(4047..4110)

/label=tracer

/note="tracer"

misc_feature complement(4050..4121)

/label=pX330 chimeric guide RNA scaffold

/note="pX330 chimeric guide RNA scaffold"

misc_feature complement(4115..4121)

/label=CRISPR end

/note="CRISPR end"

misc_feature complement(4122..4145)

/label=Lp sgRNA1 EM1096

/note="Lp gRNA3"

misc_feature complement(4146..4466)

/label=AGAP013557 U6 prom

/note="AGAP013557 U6 prom"

misc_feature complement(4169..4176)

/label=-30 TATA like

/note="-30 TATA like"

misc_feature complement(4198..4210)

/label=-65 conserved

/note="-65 conserved"

misc_feature 4471..4502

/label=from Ag U6

/note="from Ag U6"

misc_feature complement(4500..4563)

/label=tracer

/note="tracer"

misc_feature complement(4503..4574)

/label=pX330 chimeric guide RNA scaffold

/note="pX330 chimeric guide RNA scaffold"

misc_feature complement(4568..4574)

/label=CRISPR end

/note="CRISPR end"

misc_feature 4575..4598

/label=Lp sgRNA2 EM1098

/note="sgRNA2 EM1098"

misc_feature complement(4599..4919)

/label=AGAP013557 U6 prom

/note="AGAP013557 U6 prom"

misc_feature complement(4622..4629)

/label=-30 TATA like

/note="-30 TATA like"

misc_feature complement(4651..4663)

/label=-65 conserved

/note="-65 conserved"

misc_feature 4924..4955

/label=Ag U6 terminator

/note="from Ag U6"

misc_feature complement(4953..5016)

/label=tracer

/note="tracer"

misc_feature complement(4956..5027)

/label=pX330 chimeric guide RNA scaffold

/note="pX330 chimeric guide RNA scaffold"

misc_feature complement(5021..5027)

/label=CRISPR end

/note="CRISPR end"

misc_feature 5028..5051

/label=Lp sgRNA3 EM1100

/note="sgRNA3 EM1100"

misc_feature complement(5052..5372)

/label=AGAP013557 U6 prom

/note="AGAP013557 U6 prom"

misc_feature complement(5075..5082)

/label=-30 TATA like

/note="-30 TATA like"

misc_feature complement(5104..5116)

/label=-65 conserved

/note="-65 conserved"

misc_feature 5396..6619

/label=3xP3 DsRedNLS sv40

/note="3xP3 DsRedNLS sv40"

misc_feature 6623..8856

/label=vasa promoter

/note="vasa promoter"

misc_feature 8872..8940

/label=3xFLAG

/note="3xFLAG"

misc_feature 8941..8991

/label=NLS

/note="NLS"

misc_feature 8992..13092

/label=Cas9

/note="Cas9"

misc_feature 13093..13140

/label=NLS

/note="NLS"

misc_feature 13150..13376

/label=SV40 term

/note="SV40 term"

misc_feature 13409..15349

/label=Saglin 3' homology arm

/note="Saglin 3' homology arm"

misc_feature complement(15353..15408)

/label=attB2

/note="attB2"

misc_feature complement(15436..15454)

/label=M13R

/note="M13R"

misc_feature complement(15480..15498)

/label=pDONR-RP

/note="pDONR-RP"

source 1..17175

/dnas_title=“knock-in plasmid”

ORIGIN

1 ctttcctgcg ttatcccctg attctgtgga taaccgtatt accgcctttg agtgagctga

61 taccgctcgc cgcagccgaa cgaccgagcg cagcgagtca gtgagcgagg aagcggaaga

121 gcgcccaata cgcaaaccgc ctctccccgc gcgttggccg attcattaat gcagctggca

181 cgacaggttt cccgactgga aagcgggcag tgagcgcaac gcaattaata cgcgtaccgc

241 tagccaggaa gagtttgtag aaacgcaaaa aggccatccg tcaggatggc cttctgctta

301 gtttgatgcc tggcagttta tggcgggcgt cctgcccgcc accctccggg ccgttgcttc

361 acaacgttca aatccgctcc cggcggattt gtcctactca ggagagcgtt caccgacaaa

421 caacagataa aacgaaaggc ccagtcttcc gactgagcct ttcgttttat ttgatgcctg

481 gcagttccct actctcgcgt taacgctagc atggatgttt tcccagtcac gacgttgtaa

541 aacgacggcc agtcttaagc tcgggcccct acaggtcact aataccatct aagtagttga

601 ttcatagtga ctggatatgt tgtgttttac agtattatgt agtctgtttt ttatgcaaaa

661 tctaatttaa tatattgata tttatatcat tttacgtttc tcgttcaact tttctataca

721 aagttggtac cgggcccccc gctagcgtcg acggtatcga taagcttgat cggatcctgc

781 gggtgccagg gcgtgccctt gggctccccg ggcgcgtact ccacctcacc cataggcctg

841 ataacacact ggcacgacga tcatcccttt ctgctcgaaa cggtcgcgaa ggcgtacgag

901 ccgctcaagg ccgcgctgga gtcggagcag gatgcgcatc gaagccttcg gctggtgccc

961 gagctgcagc gcaagcagct catctacagc atcgaggcgg gccggctcga ggacgcccac

1021 atcctgcaca tgacgctcaa gggccagtgg aagccggacc aaatcgtgtc ggccatccag

1081 gacggctacc acgtcaaccc gacgatcatg gagcatctgc tcgagttcgt ccgcgccatc

1141 ccggtgcgga aggagcgggc cgcctactac aaagcgatcg ggccggtcat tcgcaacttc

1201 aagctgaccc tgacgtacgt gacgctcctg ttcgcgggcg atgcgaccgg cgtgtttgac

1261 acgcgaaagg atcgcgacga ctacatctcg caaccgctca ccgtgttcgt gcacatgctg

1321 cgctggcagc tggcgaacct cgagttcgag ttccacctgt cgctggcgga gcgcttcccc

1381 cgctactact cgctgcacat cgagcaaatc ttcttctttg cgcccccgca ctggcagaag

1441 gcggagaagc gccggctgtt cgagatcgcg agcctgttca aggcgaaggg gcaccggttt

1501 gcggcgatcg agcagctgct gaagtgggcg caccagtacg caccgatgcg cggtggcaaa

1561 gcccacctgg aggaaatact gcccacgctg gcgctcgaaa cggagaagct gcggcgcacg

1621 gtcgagcaga cgggcaagag ctcagcggag ctggtgcggc tcaagaagct ggaggaaaag

1681 ttcgtaagca aaaaggatcg ctggaacacg aacacctacc gcacgtacct gcacatgatc

1741 aagacgaagg gtaagttcgg ccagcatagg atggaaatgg agcgcaaggg ttaaatagcg

1801 ggaatggcac atgcgaggcg atggatgaaa tacaagtcgt ttggtgagcg gagtcaccaa

1861 gcaagcataa ctttcatgcg tacaatgtgt atgatcactg atgacaaagc gcttgggtgg

1921 ggcgcttgct gctgaagcgt ggctgacaaa tggggggggg gcttgaacaa atatttgcac

1981 acacgataat gctcttgttt gccatgcgct gacggaagac aaatcgctgc aagcgacggc

2041 tcaaacctgc tcaaaccagg gaaggtttgc ataaggttca cttataaatg agcgttcgtg

2101 gctactcggg cgttcaagtc atctcgagca ggaagcaccg cagcatgtca cgtttgccca

2161 ctgtactgtt gctcttagcg agcgccgcag tcctcgcggc tggcggccag gaagcaaccg

2221 aagacccgtt cgcggacgaa accgaccagt gccagatcag cgttagtgcc gagacgatga

2281 aatccctcca cggcgggtcg atgcagcccg acggcacgtg cgacaacctg tgggaaagct

2341 ttctatctca gtttcaccaa gtcagggaaa acctgaccgc gtgccaggag cgggcagccg

2401 ccggaccagc gcccgatcct tccagccagt tctgtcagca gctgctggac gatgcgcagc

2461 ggcagatgga gcaggagcat cgccagtacg ccgccaccct ggaggagcag ctgcacgcag

2521 cgcagcagga aacccagcag gagcaggaga tgaagaaggc gctgcagaag cagctcgacg

2581 cgctcacgta agcgtcgacg gtatcgataa gcttgatcgg atccgatgaa aataagaaaa

2641 acatttgaca aaaaaagcac cgactcggtg ccactttttc aagttgataa cggactagcc

2701 ttattttaac ttgctatttc tagctctaaa accctgcttg aagttaccgt tcaaggacga

2761 ggggaaaaaa ggttgtatat atactttgcg ctttcaatcc ttgctctagc gatgcataaa

2821 ggatattcaa gaaggatttt ttgcctgagt gtgactgtta taaggttttg atcctgccgt

2881 aactcacgct aatgtgatgt ttcataaact tttcaacatt tcttgtattg cttcatctct

2941 tttttaatca aaagttacta ttcattgtaa tacgttgaaa tatcaaaaaa gaataaacag

3001 ttttcctatg attaattcaa aaacagagtt ttctagaata accaagagcc aaaccgcttc

3061 agtgtatatg tggggaagat gaaaataaga aaaacatttg acaaaaaaag caccgactcg

3121 gtgccacttt ttcaagttga taacggacta gccttatttt aacttgctat ttctagctct

3181 aaaaccaacg cgctgtacat cgatcaagga cgaggggaaa aaaggttgta tatatacttt

3241 gcgctttcaa tccttgctct agcgatgcat aaaggatatt caagaaggat tttttgcctg

3301 agtgtgactg ttataaggtt ttgatcctgc cgtaactcac gctaatgtga tgtttcataa

3361 acttttcaac atttcttgta ttgcttcatc tcttttttaa tcaaaagtta ctattcattg

3421 taatacgttg aaatatcaaa aaagaataaa cagttttcct atgattaatt caaaaacaga

3481 gttttctaga ataaccaaga gccaaaccgc ttcagtgtat atgtggagag gatgaaaata

3541 agaaaaacat ttgacaaaaa aagcaccgac tcggtgccac tttttcaagt tgataacgga

3601 ctagccttat tttaacttgc tatttctagc tctaaaacct cgtactacaa cctgatgcaa

3661 ggacgagggg aaaaaaggtt gtatatatac tttgcgcttt caatccttgc tctagcgatg

3721 cataaaggat attcaagaag gattttttgc ctgagtgtga ctgttataag gttttgatcc

3781 tgccgtaact cacgctaatg tgatgtttca taaacttttc aacatttctt gtattgcttc

3841 atctcttttt taatcaaaag ttactattca ttgtaatacg ttgaaatatc aaaaaagaat

3901 aaacagtttt cctatgatta attcaaaaac agagttttct agaataacca agagccaaac

3961 cgcttcagtg tatatgtgga acacactggc acgacatcga taagcttgat cggatccgat

4021 gaaaataaga aaaacatttg acaaaaaaag caccgactcg gtgccacttt ttcaagttga

4081 taacggacta gccttatttt aacttgctat ttctagctct aaaacggtgt cgctagtgct

4141 gattcaagga cgaggggaaa aaaggttgta tatatacttt gcgctttcaa tccttgctct

4201 agcgatgcat aaaggatatt caagaaggat tttttgcctg agtgtgactg ttataaggtt

4261 ttgatcctgc cgtaactcac gctaatgtga tgtttcataa acttttcaac atttcttgta

4321 ttgcttcatc tcttttttaa tcaaaagtta ctattcattg taatacgttg aaatatcaaa

4381 aaagaataaa cagttttcct atgattaatt caaaaacaga gttttctaga ataaccaaga

4441 gccaaaccgc ttcagtgtat atgtggggaa gatgaaaata agaaaaacat ttgacaaaaa

4501 aagcaccgac tcggtgccac tttttcaagt tgataacgga ctagccttat tttaacttgc

4561 tatttctagc tctaaaactc cctgacgata tttcacgcaa ggacgagggg aaaaaaggtt

4621 gtatatatac tttgcgcttt caatccttgc tctagcgatg cataaaggat attcaagaag

4681 gattttttgc ctgagtgtga ctgttataag gttttgatcc tgccgtaact cacgctaatg

4741 tgatgtttca taaacttttc aacatttctt gtattgcttc atctcttttt taatcaaaag

4801 ttactattca ttgtaatacg ttgaaatatc aaaaaagaat aaacagtttt cctatgatta

4861 attcaaaaac agagttttct agaataacca agagccaaac cgcttcagtg tatatgtgga

4921 gaggatgaaa ataagaaaaa catttgacaa aaaaagcacc gactcggtgc cactttttca

4981 agttgataac ggactagcct tattttaact tgctatttct agctctaaaa cccgaggacc

5041 cacatcgttc caaggacgag gggaaaaaag gttgtatata tactttgcgc tttcaatcct

5101 tgctctagcg atgcataaag gatattcaag aaggattttt tgcctgagtg tgactgttat

5161 aaggttttga tcctgccgta actcacgcta atgtgatgtt tcataaactt ttcaacattt

5221 cttgtattgc ttcatctctt ttttaatcaa aagttactat tcattgtaat acgttgaaat

5281 atcaaaaaag aataaacagt tttcctatga ttaattcaaa aacagagttt tctagaataa

5341 ccaagagcca aaccgcttca gtgtatatgt ggataaaccc acatcgttcc gtatctaatt

5401 caattagaga ctaattcaat tagagctaat tcaattagga tccaagctta tcgatttcga

5461 accctcgacc gccggagtat aaatagaggc gcttcgtcta cggagcgaca attcaattca

5521 aacaagcaaa gtgaacacgt cgctaagcga aagctaagca aataaacaag cgcagctgaa

5581 caagctaaac aatcggggta ccgctagagt cgacggtacc gcgggcccgg gatccaccgg

5641 tcgccaccat ggcctcctcc gagaacgtca tcaccgagtt catgcgcttc aaggtgcgca

5701 tggagggcac cgtgaacggc cacgagttcg agatcgaggg cgagggcgag ggccgcccct

5761 acgagggcca caacaccgtg aagctgaagg tgaccaaggg cggccccctg cccttcgcct

5821 gggacatcct gtccccccag ttccagtacg gctccaaggt gtacgtgaag caccccgccg

5881 acatccccga ctacaagaag ctgtccttcc ccgagggctt caagtgggag cgcgtgatga

5941 acttcgagga cggcggcgtg gcgaccgtga cccaggactc ctccctgcag gacggctgct

6001 tcatctacaa ggtgaagttc atcggcgtga acttcccctc cgacggcccc gtgatgcaga

6061 agaagaccat gggctgggag gcctccaccg agcgcctgta cccccgcgac ggcgtgctga

6121 agggcgagac gcacaaggcc ctgaagctga aggacggcgg ccactacctg gtggagttca

6181 agtccatcta catggccaag aagcccgtgc agctgcccgg ctactactac gtggacgcca

6241 agctggacat cacctcccac aacgaggact acaccatcgt ggagcagtac gagcgcaccg

6301 agggccgcca ccacctgttc ctgagatctc gacccaagaa aaagcggaag gtggaggacc

6361 cgtaagatcc accggatcta gataactgat cataatcagc cataccacat ttgtagaggt

6421 tttacttgct ttaaaaaacc tcccacacct ccccctgaac ctgaaacata aaatgaatgc

6481 aattgttgtt gttaacttgt ttattgcagc ttataatggt tacaaataaa gcaatagcat

6541 cacaaatttc acaaataaag catttttttc actgcattct agttgtggtt tgtccaaact

6601 catcaatgta tcttattatc cgatcccgat gtagaacgcg agcaaattct tttccttcca

6661 tgacagcagc agctacagtg ggaagccgaa cgtcagacgt gtttgacatg ccgaactggg

6721 cgggaaaatt acagcgtgcg ctttgttttc aagcaaatca caactcgctg caaacaaaac

6781 cgttgagaaa ttgattgttt tataatttgt attgtatttt atttgttata ataaactaaa

6841 aagacatact ttttgcatat tttatacata aaaacataca tgcagcatta taaaacacat

6901 ataaaccctc cctgtagagt cccgtatcga aatcttccat cctagttgca cagtacgacg

6961 gacgagtagg ccgtgtccgt gcaaattcca gcttttagca gtcttttgct cggagcactc

7021 gcggcgagtc ggaggtttct gctgaggtgc ttagcgctaa attagccaat tgcttttgca

7081 agtgaaataa ccagccgaat agtacttcaa aactcaggta agtgaactag ttttatagaa

7141 caaatgtttg tttgttagaa gttagtgaag tgtttgtgaa aaaaatctct catttcggca

7201 aaactaacgt aactgatttc aaattgaatt attgttttgt gatgttatat tatttcatcc

7261 agttgattag tattttctta gttatgttca aaatacagtt aaattaaatt tcatttcatt

7321 tactcataaa ataatctctt ggcttattta atttttctcg aattcgcttg tattgttcag

7381 tagcacgcgc cattcgccct ttgtttcatt ttgtacctgc tcccactaac acactggcag

7441 tgcgaaacaa aagccttcgc acgcgttgct ggtattagag tgtgtgcgtg tgtgtgttga

7501 gcgctctgtc aaaatcggct gttgccgccg gtaccgaaat tgcctgttcg cacgctgttc

7561 gtaaacattc cgtggtgtgt atcgtgtgtt gtgcatgttg cgcgcctccc cccttttgat

7621 agcaggctgc cgtggctgcc gtggtgtgtg gcgcagttga gtttttggat taattttcta

7681 aggaaatggc acgagaagag cggtggcagt gtgttggttt gctctgtccc ttcctttctg

7741 tgtgaagtgt tcttacagca cagcacgtat ccaccaccgc acacagagca ggcaaggaag

7801 tggaagtgaa caagtgtgct gcgcatgcat gtgtgtgggg ggcattttag ctgagatcgt

7861 cgttatttga gaagcggtat aggggccagt cggtgtcgac gtacggaagc ggtttagttt

7921 taatccaagc gtatcccgtc gtggagtggt tgtgtggctc tgtgtgctct catatcagtt

7981 ccagagtgag gttagtagaa tcacagtcct tggccttttt cgttacaaga tatccagaag

8041 gatggcgtta tttccacagc ttaccatggt gctcttgttt gctcgaatca ggggagaaaa

8101 acagtttcgt gtttcatgaa ccgcagttgg cactggagcg gattcaaaag tcttcgatat

8161 gcaatagata agagagtcgt tggggcatag ttgggaagcc tttccgagat gtggagtttc

8221 cgagaggaga aatggtgctt tcgtgcacgt tccgggacag cgggccccgc gaagagcatc

8281 tcgttgtcgt tcatccggca ataattgatg cgaaaagcgc gcgcgccact ggcttagcgc

8341 agtgtacaca gtgatattca cctacacaca cagaggcaca cgccttcaca cgcgcgcgtg

8401 cttcaaaggc tacttcggtg gcggtgtgtg aggtcgcttg caatggacaa tgaaaatttc

8461 gctggaaaat accatcgtct ctttaggttg caatgggtgc gggtagagcg gtggtcgtcg

8521 atattggtgg tgtagtgtgt gtgtgtgtgt gtgtgtgtgt gtgtgtgtgt gtgtgtgtgt

8581 gtgtgtgtgt gtgtgtgtgt gtgtgtgtgt gtgtgtgtgt gtgtgtgtgt gcaacggcaa

8641 ttattttttg taatatttcg accatctttc tttctctctc tccacgtgct gctgctgttg

8701 ctgctgctgc tgcattgcat gttccactat tcctctcggt ttgtgcctgc ggacgccatt

8761 gctagtcgaa agagagtcgc cgttagtcgc gcttcgagca acggacacgt tttttggttg

8821 aaaccaacag cttttttcat cttcgggaga cacacagatc ccggtgccac catggactat

8881 aaggaccacg acggagacta caaggatcat gatattgatt acaaagacga tgacgataag

8941 atggccccaa agaagaagcg gaaggtcggt atccacggag tcccagcagc cgacaagaag

9001 tacagcatcg gcctggacat cggcaccaac tctgtgggct gggccgtgat caccgacgag

9061 tacaaggtgc ccagcaagaa attcaaggtg ctgggcaaca ccgaccggca cagcatcaag

9121 aagaacctga tcggagccct gctgttcgac agcggcgaaa cagccgaggc cacccggctg

9181 aagagaaccg ccagaagaag atacaccaga cggaagaacc ggatctgcta tctgcaagag

9241 atcttcagca acgagatggc caaggtggac gacagcttct tccacagact ggaagagtcc

9301 ttcctggtgg aagaggataa gaagcacgag cggcacccca tcttcggcaa catcgtggac

9361 gaggtggcct accacgagaa gtaccccacc atctaccacc tgagaaagaa actggtggac

9421 agcaccgaca aggccgacct gcggctgatc tatctggccc tggcccacat gatcaagttc

9481 cggggccact tcctgatcga gggcgacctg aaccccgaca acagcgacgt ggacaagctg

9541 ttcatccagc tggtgcagac ctacaaccag ctgttcgagg aaaaccccat caacgccagc

9601 ggcgtggacg ccaaggccat cctgtctgcc agactgagca agagcagacg gctggaaaat

9661 ctgatcgccc agctgcccgg cgagaagaag aatggcctgt tcggaaacct gattgccctg

9721 agcctgggcc tgacccccaa cttcaagagc aacttcgacc tggccgagga tgccaaactg

9781 cagctgagca aggacaccta cgacgacgac ctggacaacc tgctggccca gatcggcgac

9841 cagtacgccg acctgtttct ggccgccaag aacctgtccg acgccatcct gctgagcgac

9901 atcctgagag tgaacaccga gatcaccaag gcccccctga gcgcctctat gatcaagaga

9961 tacgacgagc accaccagga cctgaccctg ctgaaagctc tcgtgcggca gcagctgcct

10021 gagaagtaca aagagatttt cttcgaccag agcaagaacg gctacgccgg ctacattgac

10081 ggcggagcca gccaggaaga gttctacaag ttcatcaagc ccatcctgga aaagatggac

10141 ggcaccgagg aactgctcgt gaagctgaac agagaggacc tgctgcggaa gcagcggacc

10201 ttcgacaacg gcagcatccc ccaccagatc cacctgggag agctgcacgc cattctgcgg

10261 cggcaggaag atttttaccc attcctgaag gacaaccggg aaaagatcga gaagatcctg

10321 accttccgca tcccctacta cgtgggccct ctggccaggg gaaacagcag attcgcctgg

10381 atgaccagaa agagcgagga aaccatcacc ccctggaact tcgaggaagt ggtggacaag

10441 ggcgcttccg cccagagctt catcgagcgg atgaccaact tcgataagaa cctgcccaac

10501 gagaaggtgc tgcccaagca cagcctgctg tacgagtact tcaccgtgta taacgagctg

10561 accaaagtga aatacgtgac cgagggaatg agaaagcccg ccttcctgag cggcgagcag

10621 aaaaaggcca tcgtggacct gctgttcaag accaaccgga aagtgaccgt gaagcagctg

10681 aaagaggact acttcaagaa aatcgagtgc ttcgactccg tggaaatctc cggcgtggaa

10741 gatcggttca acgcctccct gggcacatac cacgatctgc tgaaaattat caaggacaag

10801 gacttcctgg acaatgagga aaacgaggac attctggaag atatcgtgct gaccctgaca

10861 ctgtttgagg acagagagat gatcgaggaa cggctgaaaa cctatgccca cctgttcgac

10921 gacaaagtga tgaagcagct gaagcggcgg agatacaccg gctggggcag gctgagccgg

10981 aagctgatca acggcatccg ggacaagcag tccggcaaga caatcctgga tttcctgaag

11041 tccgacggct tcgccaacag aaacttcatg cagctgatcc acgacgacag cctgaccttt

11101 aaagaggaca tccagaaagc ccaggtgtcc ggccagggcg atagcctgca cgagcacatt

11161 gccaatctgg ccggcagccc cgccattaag aagggcatcc tgcagacagt gaaggtggtg

11221 gacgagctcg tgaaagtgat gggccggcac aagcccgaga acatcgtgat cgaaatggcc

11281 agagagaacc agaccaccca gaagggacag aagaacagcc gcgagagaat gaagcggatc

11341 gaagagggca tcaaagagct gggcagccag atcctgaaag aacaccccgt ggaaaacacc

11401 cagctgcaga acgagaagct gtacctgtac tacctgcaga atgggcggga tatgtacgtg

11461 gaccaggaac tggacatcaa ccggctgtcc gactacgatg tggaccatat cgtgcctcag

11521 agctttctga aggacgactc catcgacaac aaggtgctga ccagaagcga caagaaccgg

11581 ggcaagagcg acaacgtgcc ctccgaagag gtcgtgaaga agatgaagaa ctactggcgg

11641 cagctgctga acgccaagct gattacccag agaaagttcg acaatctgac caaggccgag

11701 agaggcggcc tgagcgaact ggataaggcc ggcttcatca agagacagct ggtggaaacc

11761 cggcagatca caaagcacgt ggcacagatc ctggactccc ggatgaacac taagtacgac

11821 gagaatgaca agctgatccg ggaagtgaaa gtgatcaccc tgaagtccaa gctggtgtcc

11881 gatttccgga aggatttcca gttttacaaa gtgcgcgaga tcaacaacta ccaccacgcc

11941 cacgacgcct acctgaacgc cgtcgtggga accgccctga tcaaaaagta ccctaagctg

12001 gaaagcgagt tcgtgtacgg cgactacaag gtgtacgacg tgcggaagat gatcgccaag

12061 agcgagcagg aaatcggcaa ggctaccgcc aagtacttct tctacagcaa catcatgaac

12121 tttttcaaga ccgagattac cctggccaac ggcgagatcc ggaagcggcc tctgatcgag

12181 acaaacggcg aaaccgggga gatcgtgtgg gataagggcc gggattttgc caccgtgcgg

12241 aaagtgctga gcatgcccca agtgaatatc gtgaaaaaga ccgaggtgca gacaggcggc

12301 ttcagcaaag agtctatcct gcccaagagg aacagcgata agctgatcgc cagaaagaag

12361 gactgggacc ctaagaagta cggcggcttc gacagcccca ccgtggccta ttctgtgctg

12421 gtggtggcca aagtggaaaa gggcaagtcc aagaaactga agagtgtgaa agagctgctg

12481 gggatcacca tcatggaaag aagcagcttc gagaagaatc ccatcgactt tctggaagcc

12541 aagggctaca aagaagtgaa aaaggacctg atcatcaagc tgcctaagta ctccctgttc

12601 gagctggaaa acggccggaa gagaatgctg gcctctgccg gcgaactgca gaagggaaac

12661 gaactggccc tgccctccaa atatgtgaac ttcctgtacc tggccagcca ctatgagaag

12721 ctgaagggct cccccgagga taatgagcag aaacagctgt ttgtggaaca gcacaagcac

12781 tacctggacg agatcatcga gcagatcagc gagttctcca agagagtgat cctggccgac

12841 gctaatctgg acaaagtgct gtccgcctac aacaagcacc gggataagcc catcagagag

12901 caggccgaga atatcatcca cctgtttacc ctgaccaatc tgggagcccc tgccgccttc

12961 aagtactttg acaccaccat cgaccggaag aggtacacca gcaccaaaga ggtgctggac

13021 gccaccctga tccaccagag catcaccggc ctgtacgaga cacggatcga cctgtctcag

13081 ctgggaggcg acaaaaggcc ggcggccacg aaaaaggccg gccaggcaaa aaagaaaaag

13141 taagaattct agacataatc agccatacca catttgtaga ggttttactt gctttaaaaa

13201 acctcccaca cctccccctg aacctgaaac ataaaatgaa tgcaattgtt gttgttaact

13261 tgtttattgc agcttataat ggttacaaat aaagcaatag catcacaaat ttcacaaata

13321 aagcattttt cttcactgca ttctagttgt ggtttgtcca aactcatcaa tgtatctcga

13381 cgatgtaggt cacagtctcg aagccgcgac gcagctgcaa accagcctcg ccacgatcga

13441 gctgaagcac tggcgcaagt tcgaccggtt cgtaccgtac gctaacgcgc tgccccagcc

13501 agcccagcgg ctggaggtgc tgcgtgtgct gctgagccag atcggcgacc gggagaagaa

13561 gacttcccac aaatatctgg taaaggcggc acggcagttt gacatttgcg agcagtttat

13621 cggccggggc aaggtggacc aggcggtcaa gaagcagctg gaggaactgc gcgggaagtt

13681 tgcaacgttt gcgaagggca agaactacca gcactatctg agcgagtcgc gcaagtcgtc

13741 cggctaggcg tcggcgacgg tctaaaagtg attcaaagcc ccgtaggcga ggaacccccg

13801 tacgtgtcgt gtagcgcaaa cccttcacca tcatagcaca gtgaaataaa gcaagaataa

13861 aaattaaaaa aaaaaacaat tgtaatcgaa atattgagca acagcaaagc aaagcctttc

13921 ctttggatca tccgcaatcg gcacgttgcg agttgggcgg cagcgttgct tgtgcaatcg

13981 ctggacccat gctggacaaa cagatgagca cactggcctt cccagccagc taatattgac

14041 acttcattcg agctggacca ttaatcacgg cgtctgtttg cctttgggcc aagtataact

14101 agcaccacgc accgaacaga cgggctctag ttttgtttct cttgctgtga gcgtcagtta

14161 attaaaaaaa caaacctatg gatcacagca gagtgtggtg ctgtctgctt agcttcatcg

14221 caatggccgt gctgtgtagc gcatccagtg cagcatgcac gctccaggtc cccgaaacca

14281 tgataacgcg cctgctgcaa gcggatcagc ccgccggcgg ctgcgcagct gcttgggata

14341 gtctactgtc tcggctggag caaatgcgcc acaatcttac gggctgcagc gagcgcgaaa

14401 cgacgaaccc ggcaccaccc gatgataccc gcaccaacgc cccctgtcag ctgctgctga

14461 acgagctgca acggaaaggg aatcaaaccc tgctccagct cgacaaagaa atggccagca

14521 aagcactgta cgagcgcatc gagctggcca aggtggtgca ggaccaggac cgcatccaac

14581 aagcgatcga aacgatgcgc accgagcagc gaaaccaccg gcagcagctg ctactcctct

14641 acctcgatgc ggcggaccta cgacgagccc tgcaccagta taagctgctc gtcgcgcagg

14701 gcgatcgcca ccttccccag cagatagtga agttcgtgta cgccgcccca cggcacgaga

14761 accgccggct cgagaacctg ctcgacctgg tgcggcagct gccggcgcgc caggaccagc

14821 gcaccctgta ccagctgctg cagccggaaa ttatgaagcg ccccacgcag aaccagagca

14881 ccctcgccat gctgacagcg ctcgagatgg gccaggtcgt ggaggggaac ggcgagctga

14941 aaaagcagca ggacgcgatg taccagctgg tgctgaagcg ctggatgttt ctgtgcctcg

15001 ccggccagta tcgcgagata gtgcagtttg ccacgaagca cccgcgcctg ttcgagcaga

15061 tcggggcgaa gatcgccacg attcatccga agtactggtg gaagtttagc ttcacccagt

15121 tcgtgacgta cccgaacctg ttgccgctgc ccgagcagcg gctggaagcg ttccgcacca

15181 tcatgaagca gctgaagcag cgcaatggga agtttttcgc cgaccatctg gcgaagctgg

15241 caccccagat cgagcgctgc gagcagtttc tgcggcagca gaagaaggag ctggagtgga

15301 aggacgagct ggtgaagctg aagggacagt ttgccgactt tgaccgctta tcatgggtga

15361 ggtggagtac gcgcccgggg agcccaaggg cacgccctgg cacccgcagc ttatccccta

15421 tagtgagtcg tattacatgg tcatagctgt ttcctggcag ctctggcccg tgtctcaaaa

15481 tctctgatgt tacattgcac aagataaaaa tatatcatca tgaacaataa aactgtctgc

15541 ttacataaac agtaatacaa ggggtgttat gagccatatt caacgggaaa cgtcgaggcc

15601 gcgattaaat tccaacatgg atgctgattt atatgggtat aaatgggctc gcgataatgt

15661 cgggcaatca ggtgcgacaa tctatcgctt gtatgggaag cccgatgcgc cagagttgtt

15721 tctgaaacat ggcaaaggta gcgttgccaa tgatgttaca gatgagatgg tcagactaaa

15781 ctggctgacg gaatttatgc ctcttccgac catcaagcat tttatccgta ctcctgatga

15841 tgcatggtta ctcaccactg cgatccccgg aaaaacagca ttccaggtat tagaagaata

15901 tcctgattca ggtgaaaata ttgttgatgc gctggcagtg ttcctgcgcc ggttgcattc

15961 gattcctgtt tgtaattgtc cttttaacag cgatcgcgta tttcgtctcg ctcaggcgca

16021 atcacgaatg aataacggtt tggttgatgc gagtgatttt gatgacgagc gtaatggctg

16081 gcctgttgaa caagtctgga aagaaatgca taaacttttg ccattctcac cggattcagt

16141 cgtcactcat ggtgatttct cacttgataa ccttattttt gacgagggga aattaatagg

16201 ttgtattgat gttggacgag tcggaatcgc agaccgatac caggatcttg ccatcctatg

16261 gaactgcctc ggtgagtttt ctccttcatt acagaaacgg ctttttcaaa aatatggtat

16321 tgataatcct gatatgaata aattgcagtt tcatttgatg ctcgatgagt ttttctaatc

16381 agaattggtt aattggttgt aacactggca gagcattacg ctgacttgac gggacggcgc

16441 aagctcatga ccaaaatccc ttaacgtgag ttacgcgtcg ttccactgag cgtcagaccc

16501 cgtagaaaag atcaaaggat cttcttgaga tccttttttt ctgcgcgtaa tctgctgctt

16561 gcaaacaaaa aaaccaccgc taccagcggt ggtttgtttg ccggatcaag agctaccaac

16621 tctttttccg aaggtaactg gcttcagcag agcgcagata ccaaatactg ttcttctagt

16681 gtagccgtag ttaggccacc acttcaagaa ctctgtagca ccgcctacat acctcgctct

16741 gctaatcctg ttaccagtgg ctgctgccag tggcgataag tcgtgtctta ccgggttgga

16801 ctcaagacga tagttaccgg ataaggcgca gcggtcgggc tgaacggggg gttcgtgcac

16861 acagcccagc ttggagcgaa cgacctacac cgaactgaga tacctacagc gtgagctatg

16921 agaaagcgcc acgcttcccg aagggagaaa ggcggacagg tatccggtaa gcggcagggt

16981 cggaacagga gagcgcacga gggagcttcc agggggaaac gcctggtatc tttatagtcc

17041 tgtcgggttt cgccacctct gacttgagcg tcgatttttg tgatgctcgt caggggggcg

17101 gagcctatgg aaaaacgcca gcaacgcggc ctttttacgg ttcctggcct tttgctggcc

17161 ttttgctcac atgtt

//
