# Supplementary material for: A population modification gene drive targeting both Saglin and Lipophorin impairs Plasmodium transmission in Anopheles mosquitoes
Source: eLife. 2023 Dec 5;12:e93142. doi: 10.7554/eLife.93142 (PMC10786457; doi:10.7554/eLife.93142)
Supplement: Supplementary file 2. — A parental cage was assembled containing only heterozygous transgenic mosquitoes (G0, transgene frequency = 50%). Neonate larvae of subsequent generations except G5, 6, 8, 9 (N indicates the number of larvae analysed) were analyzed using COPAS flow cytometry. Gates were drawn on COPAS diagrams around clouds of larvae corresponding to homozygous, heterozygous and negative individuals according to the intensity of GFP fluorescence, and the corresponding percentage of objects in each gate was recorded. Percentages were corrected to exclude objects not corresponding to larvae. Transgene frequency per 100 chromosomes dropped from 50% in G0 to 11.35% in G17, an average loss of 2.3% per generation. [file elife-93142-supp2.docx]

| Generation | 1 | 2 | 3 | 4 | 7 | 10 | 11 | 12 | 16 | 17 |
| --- | --- | --- | --- | --- | --- | --- | --- | --- | --- | --- |
| N= | 3653 | 1714 | 1222 | 1975 | 2273 | 6852 | 9153 | 2810 | 522 | 4335 |
| % Homozygotes | 24,5 | 19,0 | 22,4 | 20,4 | 14,1 | 7,4 | 7,8 | 5,4 | 0,7 | 1,1 |
| % Heterozygotes | 49,1 | 53,9 | 46,4 | 46,2 | 47,8 | 42,9 | 39,3 | 39,5 | 21,2 | 20,5 |
| % Negatives | 26,4 | 27,1 | 31,2 | 33,4 | 38,1 | 49,7 | 53,0 | 55,1 | 78,1 | 78,3 |
| Tsg frequency | 0,49 | 0,46 | 0,46 | 0,44 | 0,38 | 0,29 | 0,27 | 0,25 | 0,11 | 0,11 |

**Supplementary File 2: Tracking the evolution dynamics of *Lp::Sc2A10* transgene frequency**. A parental cage was assembled containing only heterozygous transgenic mosquitoes (G0, transgene frequency =50%). Neonate larvae of subsequent generations except G5, 6, 8, 9 (N indicates the number of larvae analysed) were analyzed using COPAS flow cytometry. Gates were drawn on COPAS diagrams around clouds of larvae corresponding to homozygous, heterozygous and negative individuals according to the intensity of GFP fluorescence, and the corresponding percentage of objects in each gate was recorded. Percentages were corrected to exclude objects not corresponding to larvae. Transgene frequency per 100 chromosomes dropped from 50% in G0 to 11.35% in G17, an average loss of 2.3% per generation.
