# Supplementary material for: A population modification gene drive targeting both Saglin and Lipophorin impairs Plasmodium transmission in Anopheles mosquitoes
Source: eLife. 2023 Dec 5;12:e93142. doi: 10.7554/eLife.93142 (PMC10786457; doi:10.7554/eLife.93142)
Supplement: Supplementary file 3. — Indicated identical numbers of virgin transgenic and WT females were mixed in cages with WT males. After blood feeding, neonate larvae produced by each cage were analyzed by flow cytometry (COPAS) and the numbers of GFP fluorescent and negative larvae were counted using WinMDI software on COPAS files. Identical fertility of the two categories of females would produce 50% of GFP positive progeny. a, b after the replicate number indicate the first and second egg batch, respectively, from the same mosquitoes. Replicate 1 was composed of smaller mosquitoes, due to higher density larval rearing. All other replicates were performed with mosquitoes of standard size. Replicate 4b was performed with older mosquitoes, with >50% of females already dead. [file elife-93142-supp3.docx]

| Replicate | Number of tsg ♀ + WT ♀ in cage | Number of WT ♂ | Number of GFP^+^ progeny | Number of GFP^-^ progeny |
| --- | --- | --- | --- | --- |
| 1a | 66 + 66 | 100 | 426 (23.5 %) | 1385 |
| 1b | 66 + 66 | 100 | 378 (26.8%) | 1031 |
| 2a | 53 + 53 | 94 | 240 (10.4 %) | 2060 |
| 2b | 53 + 53 | 94 | 621 (29.6%) | 1471 |
| 3a | 140+140 | 200 | 4170 (39.7%) | 6323 |
| 3b | 140+140 | 200 | 910 (45.6%) | 1084 |
| 4a | 71+71 | 132 | 2378 (39.1 %) | 3693 |
| 4b | 71+71 | 132 | 62 (8.9 %) | 700 |

**Supplementary File 3: fertility tests comparing the number of progeny produced by homozygous *Lp::Sc2A10* female versus WT female mosquitoes.** Indicated identical numbers of virgin transgenic and WT females were mixed in cages with WT males. After blood feeding, neonate larvae produced by each cage were analyzed by flow cytometry (COPAS) and the numbers of GFP fluorescent and negative larvae were counted using WinMDI software on COPAS files. Identical fertility of the two categories of females would produce 50% of GFP positive progeny. a, b after the replicate number indicate the first and second egg batch, respectively, from the same mosquitoes. Replicate 1 was composed of smaller mosquitoes, due to higher density larval rearing. All other replicates were performed with mosquitoes of standard size. Replicate 4b was performed with older mosquitoes, with >50% of females already dead.
