# Supplementary material for: A population modification gene drive targeting both Saglin and Lipophorin impairs Plasmodium transmission in Anopheles mosquitoes
Source: eLife. 2023 Dec 5;12:e93142. doi: 10.7554/eLife.93142 (PMC10786457; doi:10.7554/eLife.93142)

Inheritance dynamics of the two transgenes *SagGDvasa* (DsRed) and *Lp::Sc2A10* (GFP) in 8 mosquito populations, tracked by COPAS flow cytometry

Typical COPAS analysis :

Objects separated according to size and opacity

Objects from first gate separated according to fluorescence

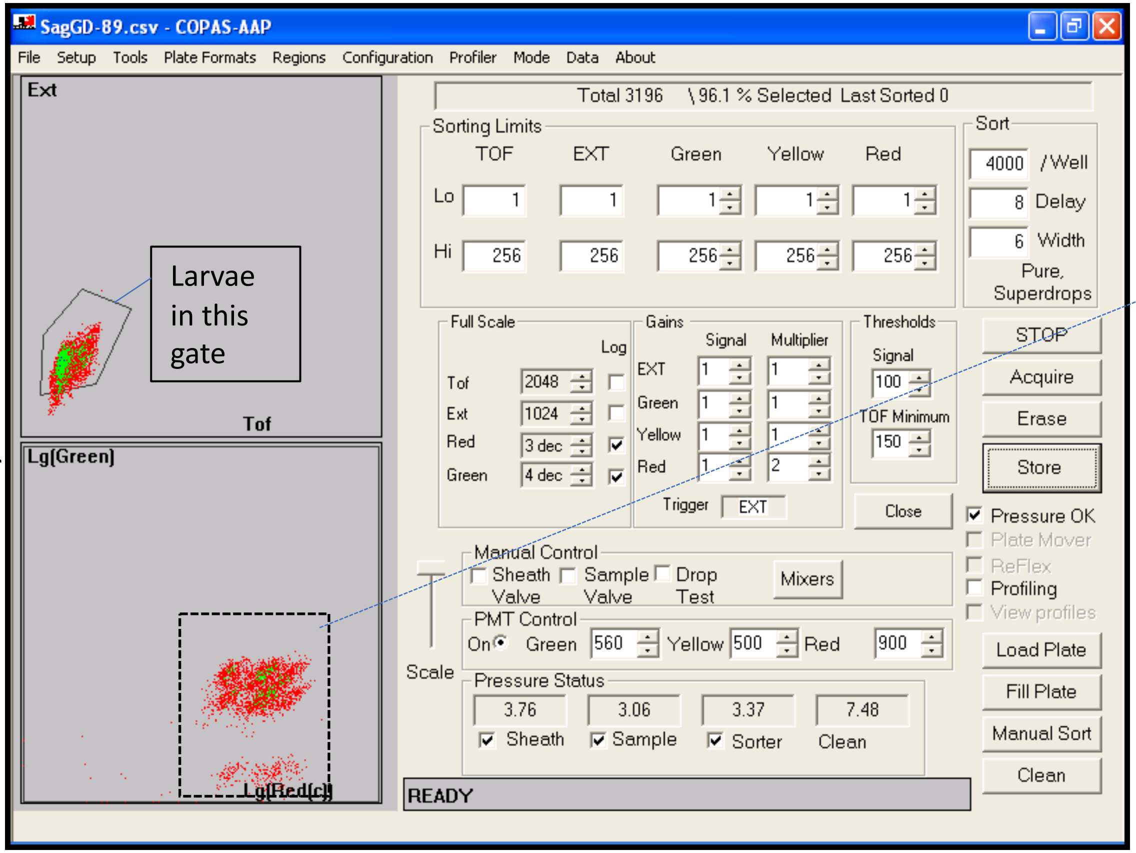

DsRed intensity

Extract screenshots of region with larvae

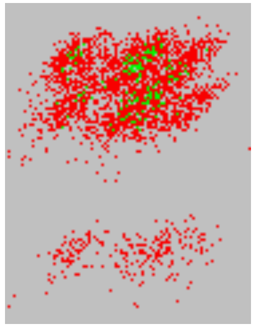

Interpretation of fluorescence :

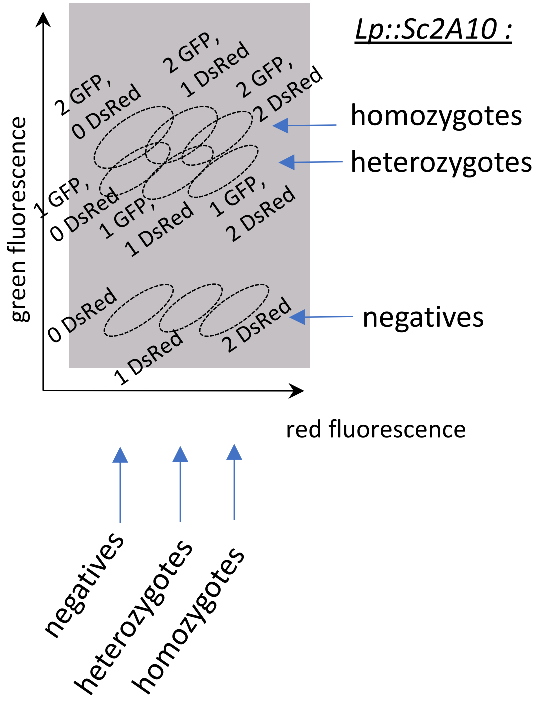

Population 1 : G0 = ♂ $\frac{SagGD^{vasa}}{Y}$; $\frac{Lp::Sc2A10}{+}$ x ♀ WT

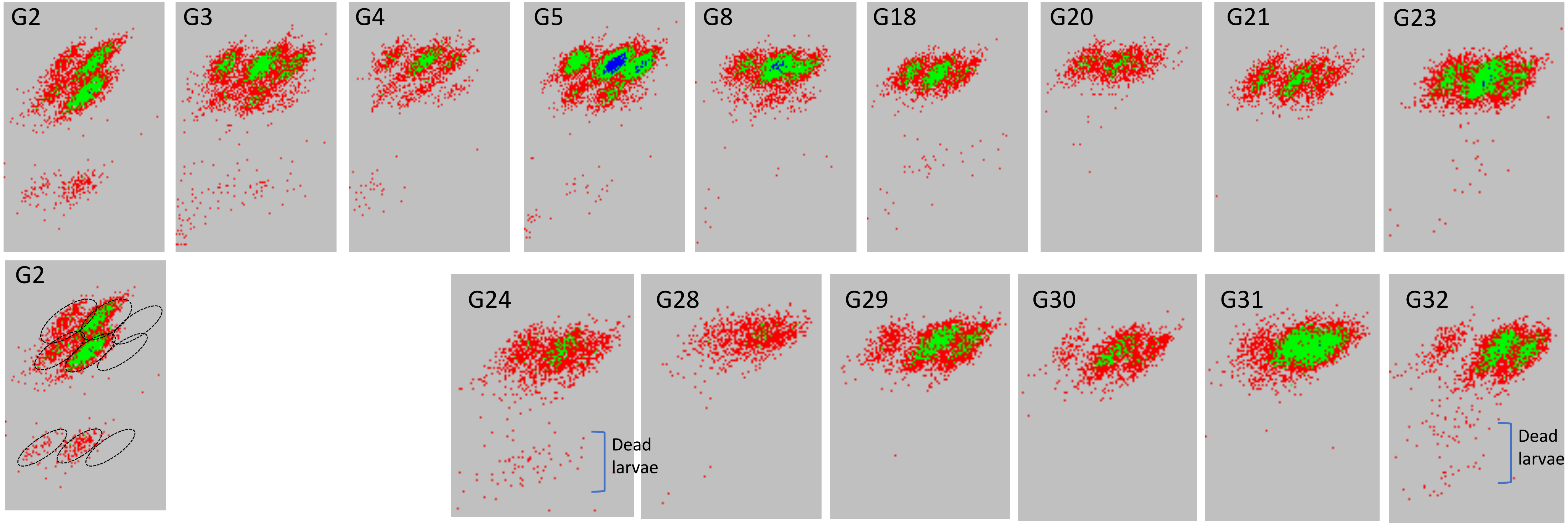

Population 2 : G0 = ♂ $\frac{SagGD^{vasa}}{Y}$; $\frac{Lp::Sc2A10}{+}$ x ♀ WT

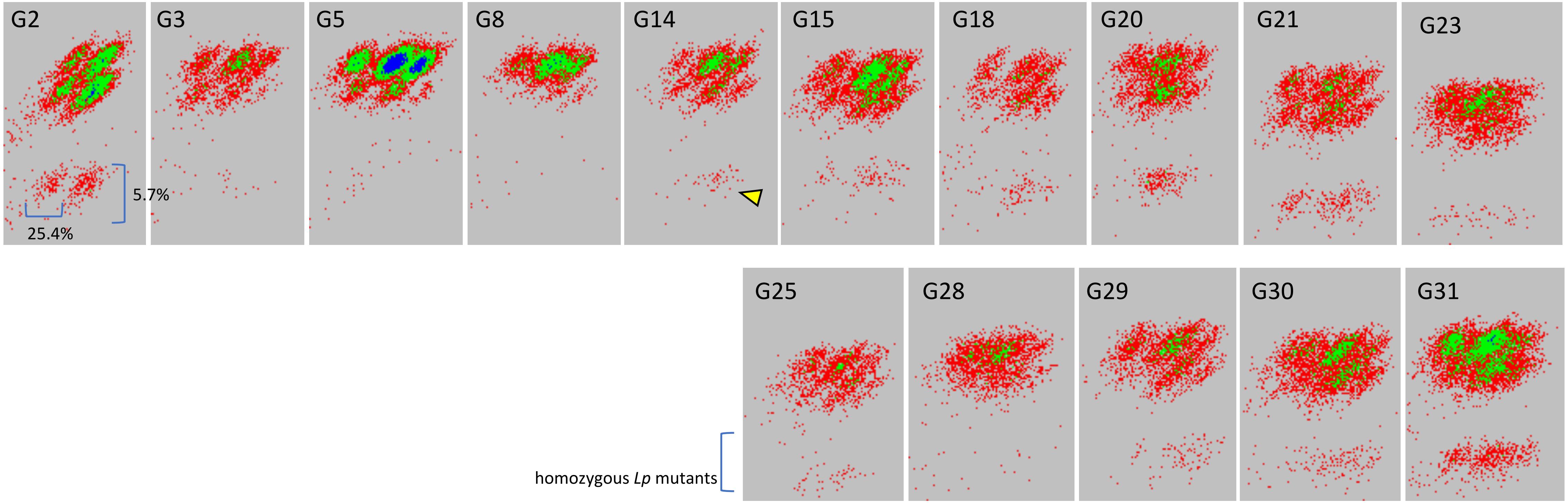

Population 3 : G0 = ~ 1:3 mix of G10 individuals from Population 1 : WT

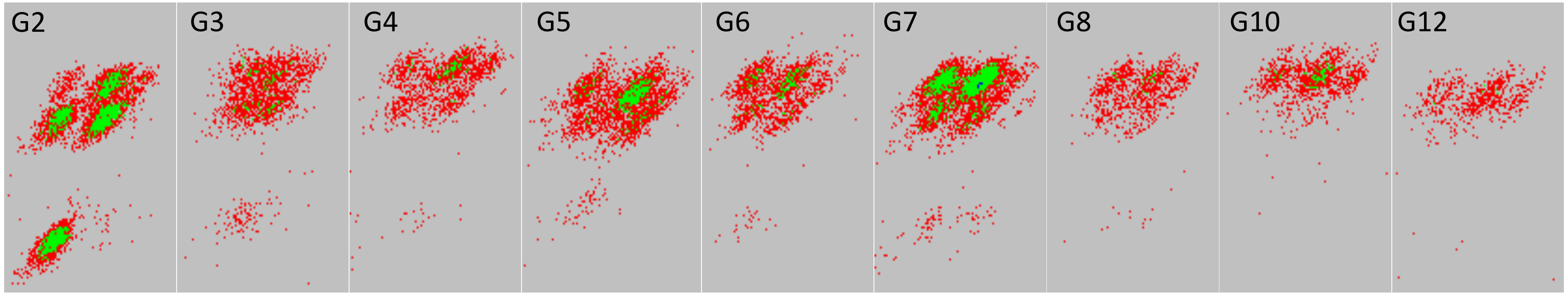

Population 4 : G0 = ~ 1:2 mix G10 individuals from Population 1 : WT

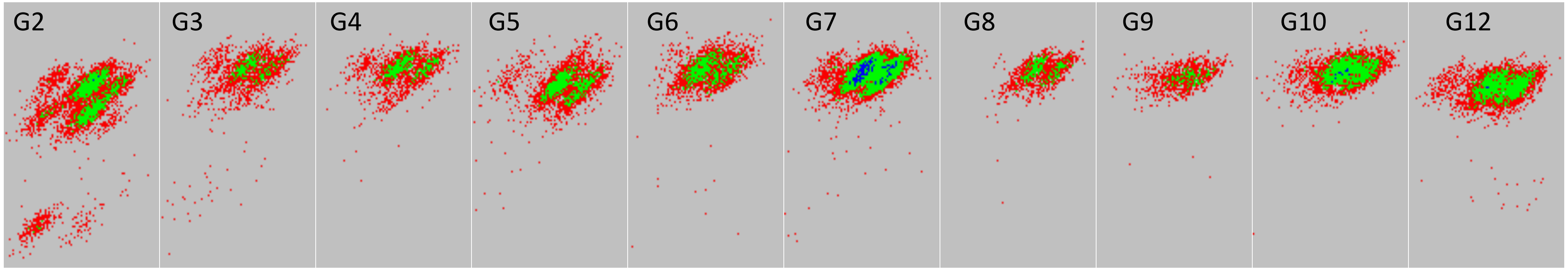

Population 5 : G0 = ♂ $\frac{SagGD^{vasa}}{Y}$; $\frac{Lp::Sc2A10}{Lp::Sc2A10}$ x ♀ WT

(from G16 of Population 1)

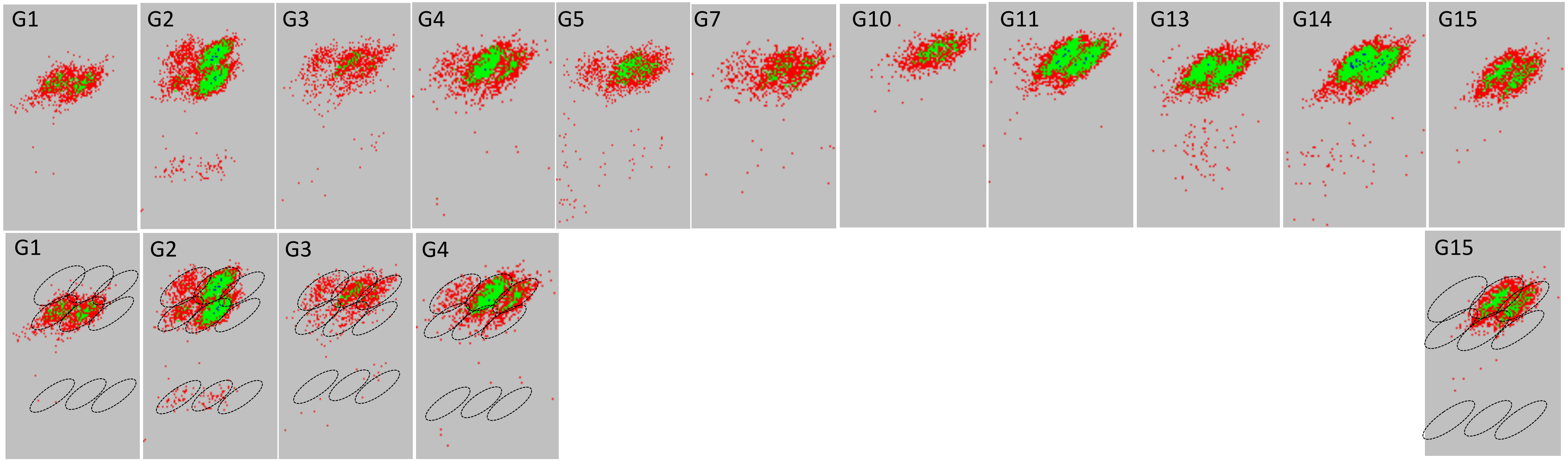

Population 6 : G0 = ♀ $\frac{SagGD^{vasa}}{SagGD^{vasa}}$; $\frac{Lp::Sc2A10}{Lp::Sc2A10}$ x ♂ WT

(from G16 of Population 1)

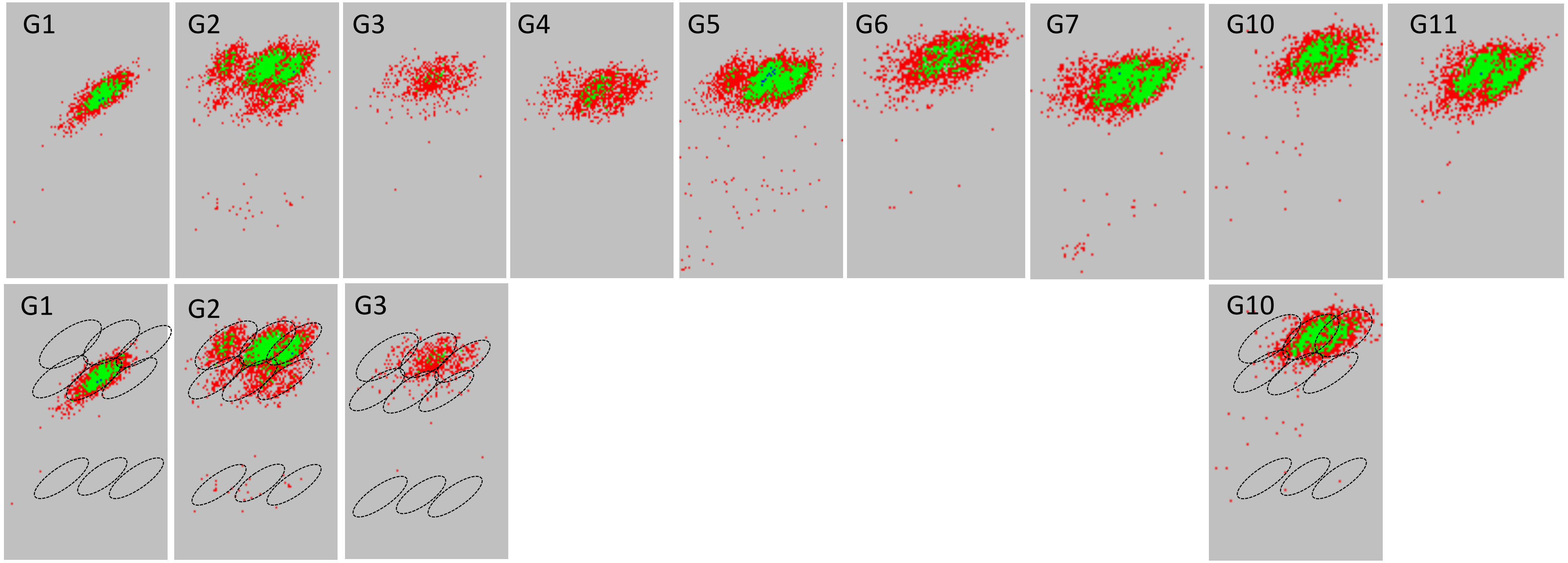

Population 7 : G0 = ♂ $\frac{SagGD^{vasa}}{Y}$; $\frac{Lp::Sc2A10}{+}$ x ♀ WT

(from G1 of Population 5)

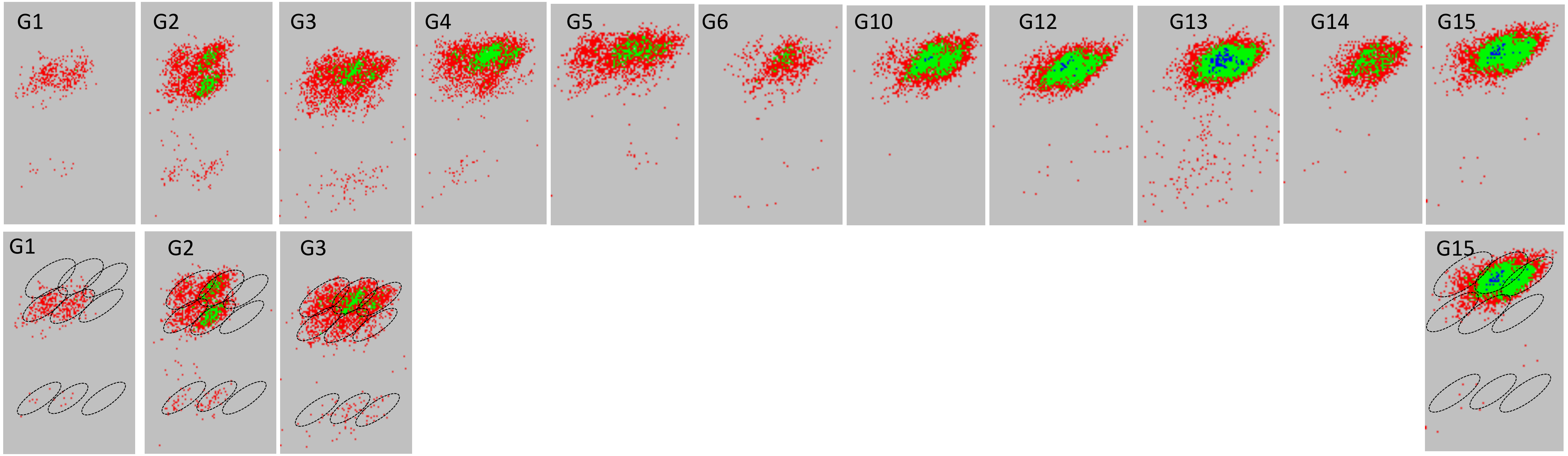

Population 8 : G0 = ♀ $\frac{SagGD^{vasa}}{+}$; $\frac{Lp::Sc2A10}{+}$ x ♂ WT

(from G1 of Population 5)

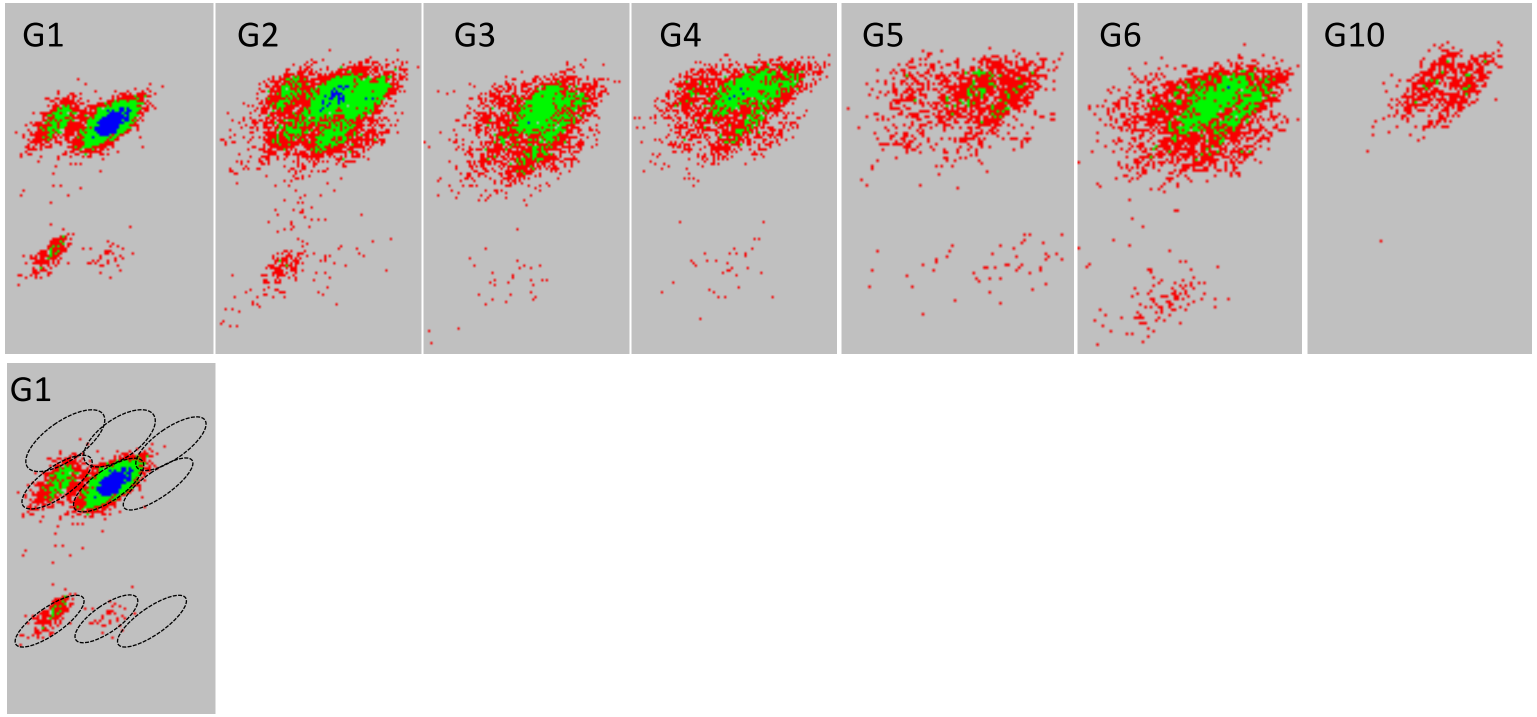

Supplement: Supplementary file 5. — Each strip of panels on successive pages corresponds to an independent caged mosquito population. Each panel is a COPAS analysis snapshot of neonate larvae at the indicated generation, obtained as shown on the first page. Successive panels provide a global view of each transgene’s evolution on samples of 1000–4000 neonate larvae at each generation, with fluorescence intensity correlating with transgene copy number (GFP marks the Lp::Sc2A10 transgene on the Y axis, DsRed marks Sag-GDvasa on the X axis). Up to 9 partially overlapping distinct larval populations corresponding to the 9 possible genotypes (0, 1, or 2 copies of each fluorescence, sketched on page 1 and on selected panels duplicated below the original panel) can be resolved on COPAS diagrams. Dots indicated in population 1 panels correspond to debris (dead larvae, egg shells) and are difficult to distinguish from non-fluorescent larvae unless sorting them for verification under the microscope. Yellow arrow in population 2, G14 indicates the appearance of GFP negative larvae that represent a Lp homing refractory mutant (see text). Tracking Populations 1 and 2 started shortly after initial transgenesis, by crossing G2 [Sag-GDvasa / Y; Lp::Sc2A10 / +] males to WT females (considered the G0 cross for temporal tracking of genotypes). Population 3 was assembled by mixing 20 transgenics from generation 12 of population 2 with 190 WT. Population 4 was assembled by mixing 60 transgenics from generation 12 of population 2 with 189 WT. In these new G0 mixes reproductive success of transgenics proved higher than that of the WT (due to differences in the quality of the parental mosquitoes), as G1 pupae consisted of 189 GFP transgenics for 358 negatives = 34.5% instead of expected 10% (population 3) or 346 GFP transgenics for 304 negatives = 53.2% instead of expected 24.1% (population 4). In all populations, note the initial rapid convergence of genotypes towards Lp::Sc2A10 (GFP) homozygosity, the rapid initial disappea [file elife-93142-supp5.pdf]
