# Supplementary material for: A population modification gene drive targeting both Saglin and Lipophorin impairs Plasmodium transmission in Anopheles mosquitoes
Source: eLife. 2023 Dec 5;12:e93142. doi: 10.7554/eLife.93142 (PMC10786457; doi:10.7554/eLife.93142)
Supplement: MDAR checklist [file elife-93142-mdarchecklist1.pdf]

Westbrook Centre
Milton Road
Cambridge CB4 1YG, UK

P 01223 855340
W elifesciences.org
T @elife

eLife

Materials Design Analysis Reporting (MDAR) Checklist for Authors

The MDAR framework establishes a minimum set of requirements in transparent reporting mainly applicable to studies in the life sciences.

eLife asks authors to **provide detailed information within their article** to facilitate the interpretation and replication of their work. Authors can also upload supporting materials to comply with relevant reporting guidelines for health-related research (see EQUATOR Network), life science research (see the BioSharing Information Resource), or animal research (see the ARRIVE Guidelines and the STRANGE Framework; for details, see *eLife*'s Journal Policies). Where applicable, authors should refer to any relevant reporting standards materials in this form.

For all that apply, please note **where in the article** the information is provided. Please note that we also collect information about data availability and ethics in the submission form.

Materials:

Newly created materials	Indicate where provided: section/figure legend	N/A
- Transgenic mosquito line Lp::2A10<br>- Transgenic mosquito line [SagGDvasa; Lp::2A10]<br>- Transgenic mosquito line pDSAY-vas2-Cas9<br>- Transgenic mosquito line pDSAP-vas2-Cas9<br>- Transgenesis plasmids	**Materials and Methods**<br>*These mosquito lines are maintained in our insectary and are available upon request.*<br><br>*Full plasmid sequences are provided in Supplemental File 1 and plasmids are available upon request.*	

**Antibodies**	**Indicate where provided: section/figure legend**	**N/A**
- Monoclonal antibody against ApoLipophorinII 2H5<br>- Anopheles-saliva reactive Human Serum<br>- Saglin Rabbit polyclonal antibody	2H5 (Rono et al., 2010) and human serum produced in our laboratory,<br>available upon request.<br>Saglin antibody from Eric Calvo, NIH-NIAID, Rockville, USA	

**DNA and RNA sequences**	**Indicate where provided: section/figure legend**	**N/A**
PCR primers .	All primer sequences are provided in Materials and Methods	

**Cell materials**	**Indicate where provided: section/figure legend**	**N/A**
Cell lines: Provide species information, strain. Provide accession number in repository OR supplier name, catalog number, clone number, OR RRID.		N/A
Primary cultures: Provide species, strain, sex of origin, genetic modification status.		N/A

**Experimental animals**	**Indicate where provided: section/figure legend**	**N/A**
CD1 mice for Plasmodium infection and bite-back assays	Materials & Methods	
Animal observed in or captured from the field: Provide species, sex, and age where possible.		N/A

Plants and microbes	Indicate where provided: section/figure legend	N/A
Plants: provide species and strain, ecotype and cultivar where relevant, unique accession number if available, and source (including location for collected wild specimens).		N/A
Microbes: provide species and strain, unique accession number if available, and source.		N/A

Human research participants	Indicate where provided: section/figure legend) or state if these demographics were not collected	N/A
If collected and within the bounds of privacy constraints report on age, sex, gender and ethnicity for all study participants.		N/A

Design:

Study protocol	Indicate where provided: section/figure legend	N/A
If the study protocol has been pre-registered, provide DOI. For clinical trials, provide the trial registration number OR cite DOI.		N/A

Laboratory protocol	Indicate where provided: section/figure legend	N/A
Provide DOI OR other citation details if detailed step-by-step protocols are available.		N/A

Experimental study design (statistics details) *		
**For in vivo studies: State whether and how the following have been done**	**Indicate where provided: section/figure legend. If it could have been done, but was not, write "not done"**	**N/A**
Sample size determination	Not done	
Randomisation		**N/A**
Blinding		N/A

Inclusion/exclusion criteria		N/A

**Sample definition and in-laboratory replication**	**Indicate where provided: section/figure legend**	**N/A**
State number of times the experiment was replicated in the laboratory.	Bite-back experiments were performed at least 16 times, with variations in the mosquito genotypes and number of mice as indicated in Supplemental Table 2.<br><br>Mosquito cage experiments to follow gene drive dynamics were performed on 8 populations as described in Results, Figure 5 and Supplemental File 2.	
Define whether data describe technical or biological replicates.	Data describe biological replicates (individual mice, distinct mosquito populations).	

**Ethics**	**Indicate where provided: section/submission form**	**N/A**
Studies involving human participants: State details of authority granting ethics approval (IRB or equivalent committee(s), provide reference number for approval.		N/A
Studies involving experimental animals: State details of authority granting ethics approval (IRB or equivalent committee(s), provide reference number for approval.	Work on mice was evaluated by the CREMEAS Ethics committee and authorized by Ministère de l'Enseignement Supérieur et de la Recherche (MESRI) under reference APAFIS #20562-2019050313288887v3. Work with genetically modified mosquitoes was evaluated by Haut Conseil des Biotechnologies and authorized by MESRI (agréments d'utilisation d'OGM en milieu confiné #3243 and #3912).	

Studies involving specimen and field samples: State if relevant permits obtained, provide details of authority approving study; if none were required, explain why.		N/A

**Dual Use Research of Concern (DURC)**	**Indicate where provided: section/submission form**	**N/A**
If study is subject to dual use research of concern regulations, state the authority granting approval and reference number for the regulatory approval.		N/A

Analysis:

**Attrition**	**Indicate where provided: section/figure legend**	**N/A**
Describe whether exclusion criteria were pre-established. Report if sample or data points were omitted from analysis. If yes, report if this was due to attrition or intentional exclusion and provide justification.		N/A

**Statistics**	**Indicate where provided: section/figure legend**	**N/A**
Describe statistical tests used and justify choice of tests.	Percentage of infected mice were comparted using Fisher's exact test with a two-tailed p-value (see Legend of Figure 2).	

**Data availability**	**Indicate where provided: section/submission form**	**N/A**
For newly created and reused datasets, the manuscript includes a data availability statement that provides details for access (or notes restrictions on access).	All data associated with this study are present in the paper or the Supplementary Materials.	
When newly created datasets are publicly available, provide accession number in repository OR DOI and licensing details where available.		N/A
If reused data is publicly available provide accession number in repository OR DOI, OR URL, OR citation.		N/A

Code availability	Indicate where provided: section/figure legend	N/A
For any computer code/software/mathematical algorithms essential for replicating the main findings of the study, whether newly generated or re-used, the manuscript includes a data availability statement that provides details for access or notes restrictions.	PCR amplicon sequence analyses were performed as described in Materials & Methods in section “Amplicon sequencing”	
Where newly generated code is publicly available, provide accession number in repository, OR DOI OR URL and licensing details where available. State any restrictions on code availability or accessibility.		N/A
If reused code is publicly available provide accession number in repository OR DOI OR URL, OR citation.		N/A

Reporting:

The MDAR framework recommends adoption of discipline-specific guidelines, established and endorsed through community initiatives.

Adherence to community standards	Indicate where provided: section/figure legend	N/A
State if relevant guidelines (e.g., ICMJE, MIBBI, ARRIVE, STRANGE) have been followed, and whether a checklist (e.g., CONSORT, PRISMA, ARRIVE) is provided with the manuscript.		N/A
